# Predicting Hospitalization and Related Outcomes in Advanced Chronic Kidney Disease: A Systematic Review, External Validation, and Development Study

**DOI:** 10.1016/j.xkme.2025.101016

**Published:** 2025-04-25

**Authors:** Roemer J. Janse, Jet Milders, Joris I. Rotmans, Fergus J. Caskey, Marie Evans, Claudia Torino, Maciej Szymczak, Christiane Drechsler, Christoph Wanner, Maria Pippias, Antonio Vilasi, Vianda S. Stel, Nicholas C. Chesnaye, Kitty J. Jager, Friedo W. Dekker, Merel van Diepen, EQUAL study investigators, Andreas Schneider, Anke Torp, Beate Iwig, Boris Perras, Christian Marx, Christiane Drechsler, Christof Blaser, Christoph Wanner, Claudia Emde, Detlef Krieter, Dunja Fuchs, Ellen Irmler, Eva Platen, Hans Schmidt-Gürtler, Hendrik Schlee, Holger Naujoks, Ines Schlee, Sabine Cäsar, Joachim Beige, Jochen Röthele, Justyna Mazur, Kai Hahn, Katja Blouin, Katrin Neumeier, Kirsten Anding-Rost, Lothar Schramm, Monika Hopf, Nadja Wuttke, Nikolaus Frischmuth, Pawlos Ichtiaris, Petra Kirste, Petra Schulz, Sabine Aign, Sandra Biribauer, Sherin Manan, Silke Röser, Stefan Heidenreich, Stephanie Palm, Susanne Schwedler, Sylke Delrieux, Sylvia Renker, Sylvia Schättel, Theresa Stephan, Thomas Schmiedeke, Thomas Weinreich, Til Leimbach, Torsten Stövesand, Udo Bahner, Wolfgang Seeger, Adamasco Cupisti, Adelia Sagliocca, Alberto Ferraro, Alessandra Mele, Alessandro Naticchia, Alex Còsaro, Andrea Ranghino, Andrea Stucchi, Angelo Pignataro, Antonella De Blasio, Antonello Pani, Aris Tsalouichos, Bellasi Antonio, Biagio Raffaele Di Iorio, Butti Alessandra, Cataldo Abaterusso, Chiara Somma, Claudia D’alessandro, Claudia Torino, Claudia Zullo, Claudio Pozzi, Daniela Bergamo, Daniele Ciurlino, Daria Motta, Domenico Russo, Enrico Favaro, Federica Vigotti, Ferruccio Ansali, Ferruccio Conte, Francesca Cianciotta, Francesca Giacchino, Francesco Cappellaio, Francesco Pizzarelli, Gaetano Greco, Gaetana Porto, Giada Bigatti, Giancarlo Marinangeli, Gianfranca Cabiddu, Giordano Fumagalli, Giorgia Caloro, Giorgina Piccoli, Giovanbattista Capasso, Giovanni Gambaro, Giuliana Tognarelli, Giuseppe Bonforte, Giuseppe Conte, Giuseppe Toscano, Goffredo Del Rosso, Irene Capizzi, Ivano Baragetti, Lamberto Oldrizzi, Loreto Gesualdo, Luigi Biancone, Manuela Magnano, Marco Ricardi, Maria Di Bari, Maria Laudato, Maria Luisa Sirico, Martina Ferraresi, Michele Provenzano, Moreno Malaguti, Nicola Palmieri, Paola Murrone, Pietro Cirillo, Pietro Dattolo, Pina Acampora, Rita Nigro, Roberto Boero, Roberto Scarpioni, Rosa Sicoli, Rosella Malandra, Silvana Savoldi, Silvio Bertoli, Silvio Borrelli, Stefania Maxia, Stefano Maffei, Stefano Mangano, Teresa Cicchetti, Tiziana Rappa, Valentina Palazzo, Walter De Simone, Anita Schrander, Bastiaan van Dam, Carl Siegert, Carlo Gaillard, Charles Beerenhout, Cornelis Verburgh, Cynthia Janmaat, Ellen Hoogeveen, Ewout Hoorn, Friedo Dekker, Johannes Boots, Henk Boom, Jan-Willem Eijgenraam, Jeroen Kooman, Joris Rotmans, Kitty Jager, Liffert Vogt, Maarten Raasveld, Marc Vervloet, Marjolijn van Buren, Merel van Diepen, Nicholas Chesnaye, Paul Leurs, Pauline Voskamp, Peter Blankestijn, Sadie van Esch, Siska Boorsma, Stefan Berger, Constantijn Konings, Zeynep Aydin, Aleksandra Musiała, Anna Szymczak, Ewelina Olczyk, Hanna Augustyniak-Bartosik, Ilona Miśkowiec-Wiśniewska, Jacek Manitius, Joanna Pondel, Kamila Jędrzejak, Katarzyna Nowańska, Łukasz Nowak, Maciej Szymczak, Magdalena Durlik, Ś zyszkowska Dorota, Teresa Nieszporek, Zbigniew Heleniak, Andreas Jonsson, Anna-Lena Blom, Björn Rogland, Carin Wallquist, Denes Vargas, Emöke Dimény, Fredrik Sundelin, Fredrik Uhlin, Gunilla Welander, Isabel Bascaran Hernandez, Knut-Christian Gröntoft, Maria Stendahl, Maria Svensson, Marie Evans, Olof Heimburger, Pavlos Kashioulis, Stefan Melander, Tora Almquist, Ulrika Jensen, Alistair Woodman, Anna McKeever, Asad Ullah, Barbara McLaren, Camille Harron, Carla Barrett, Charlotte O’Toole, Christina Summersgill, Colin Geddes, Deborah Glowski, Deborah McGlynn, Dympna Sands, Fergus Caskey, Geena Roy, Gillian Hirst, Hayley King, Helen McNally, Houda Masri-Senghor, Hugh Murtagh, Hugh Rayner, Jane Turner, Joanne Wilcox, Jocelyn Berdeprado, Jonathan Wong, Joyce Banda, Kirsteen Jones, Lesley Haydock, Lily Wilkinson, Margaret Carmody, Maria Weetman, Martin Joinson, Mary Dutton, Michael Matthews, Neal Morgan, Nina Bleakley, Paul Cockwell, Paul Roderick, Phil Mason, Philip Kalra, Rincy Sajith, Sally Chapman, Santee Navjee, Sarah Crosbie, Sharon Brown, Sheila Tickle, Suresh Mathavakkannan, Ying Kuan

**Affiliations:** 1 Department of Clinical Epidemiology, Leiden University Medical Center, Leiden, the Netherlands; 2 Department of Internal Medicine, Leiden University Medical Center, Leiden, the Netherlands; 3 Population Health Sciences, Bristol Medical School, University of Bristol, United Kingdom; 4 Renal Unit, Department of Clinical Intervention and Technology (CLINTEC), Karolinska Institutet and Karolinska University Hospital, Stockholm, Sweden; 5 IFC-CNR, Clinical Epidemiology and Pathophysiology of Renal Diseases and Hypertension, Reggio Calabria, Italy; 6 Department of Nephrology and Transplantation Medicine, Wroclaw Medical University, Wroclaw, Poland; 7 Division of Nephrology, University Hospital of Würzburg, Würzburg, Germany; 8 Department of Clinical Research and Epidemiology, Comprehensive Heart Failure Center, University Hospital of Würzburg, Würzburg, Germany; 9 North Bristol NHS Trust, Renal Unit, Bristol, United Kingdom; 10 ERA Registry, Amsterdam UMC location University of Amsterdam, Medical Informatics, Amsterdam, the Netherlands; 11 Amsterdam Public Health Research Institute, Quality of Care, Amsterdam, the Netherlands

**Keywords:** Algorithm, chronic kidney disease, dialysis, hospital admission, hospitalization, length of stay, prediction, readmission, risk score

## Abstract

**Rationale & Objective:**

Hospitalization is common in patients with advanced chronic kidney disease (CKD). Predicting hospitalization and related outcomes would be beneficial for hospitals and patients. Therefore, we aimed to (1) give an overview of current prediction models for hospitalization, length of stay, and readmission in patients with advanced CKD; (2) externally validate these models; and (3) develop a new model if no valid models were identified.

**Study Design:**

Systematic review, development, and external validation study.

**Setting & Participants:**

We were interested in prediction models of hospitalization, length of stay, or readmission for patients with advanced CKD. Our available development and validation data consisted of hemodialysis, peritoneal dialysis, and advanced CKD patients not receiving dialysis from a Dutch dialysis and European advanced CKD cohort.

**Selection Criteria for Studies:**

We systematically searched PubMed. Studies had to intentionally develop, validate, or update a prediction model in adults with CKD.

**Analytical Approach:**

We used the PROBAST for risk of bias assessment. Identified models were externally validated on model discrimination (C-statistic) and calibration (calibration plot, slope, and calibration-in-the-large). We developed a Fine-Gray model for hospitalization within 1 year in patients initiating hemodialysis, accounting for the competing risk of death.

**Results:**

We identified 45 models in 8 studies. The majority were of low quality with a high risk of bias. Due to underreporting and population-specific predictors, we could only validate 3 models. These were poorly calibrated and had poor discrimination. Using multiple modeling strategies, an adequate new model could not be developed.

**Limitations:**

The outcome hospitalization might be too heterogeneous, and we did not have all relevant predictors available.

**Conclusions:**

Hospitalizations are important but difficult to predict for patients with advanced CKD. An improved prediction model should be developed, for example, using a more specific outcome (eg, cardiovascular hospitalizations) and more predictors (eg, patient-reported outcome measures).

## Plain-Language Summary

Hospitalizations often occur in patients with advanced chronic kidney disease. By predicting hospitalization and related outcomes, patients can better prepare for the future and cope with their disease. Therefore, we searched existing literature for existing methods to predict hospitalizations and related outcomes. Although many algorithms exist, they are often not available for use or are not reliable. We then developed our own algorithm to predict hospitalization in the coming year. However, it also did not predict reliably. In this study, we summarize what failed in existing prediction algorithms, what we learned from predicting hospitalization ourselves, and how to proceed to allow reliable predictions of hospitalizations.

Chronic kidney disease (CKD) affects >10% of people worldwide and is a leading cause of years of life lost and death.^1^ Moreover, CKD may lead to other adverse outcomes, such as cardiovascular events and hospitalizations, which increasingly occur with decreasing kidney function.^2^ Moreover, patients with CKD often have a longer length of stay (LOS) in the hospital and are more often readmitted to the hospital: hospitalized patients with CKD have 20% higher risk of readmission, dialysis patients have 76% higher risk, and patients with a kidney transplant have 85% higher risk than hospitalized individuals without CKD.^3^

As increased risk of hospitalization has a strong impact on health care planning,^4^ being able to anticipate hospitalizations may assist hospitals and health care providers in adequate health care planning. Moreover, CKD patients are highly affected by hospitalizations, with hospitalizations possibly being a traumatic experience and reducing quality of life.^5^, ^6^, ^7^ In the Standardized Outcomes in NephroloGy (SONG) initiative,^8^ patients indeed indicated that they wanted more information about their risk of hospitalization.^9^^,^^10^

Knowing what to expect (eg, hospitalization risk or expected LOS) could support patients in preparing for their future and coping with their disease. Prediction models that focus on predicting outcomes relevant to hospitalizations, such as the risk of hospitalization, predicted LOS, or probability of being readmitted, can provide such risk estimates. Readmission models may further aid in prescribing post-discharge medication and shared decision making regarding follow-up visits. The majority of prediction models in CKD have focused on outcomes traditionally of interest to the health care provider, such as disease progression,^11^, ^12^, ^13^ mortality,^14^, ^15^, ^16^ and cardiovascular events.^17^, ^18^, ^19^ Moreover, prediction models for the general population might not perform as well in a disease-specific population.^20^ However, a recent Kidney Disease: Improving Global Outcomes report urged attention toward patient-relevant outcomes such as hospitalizations.^21^ An overview of current prediction models for hospitalization is needed to determine whether this need for prediction models is being met and to identify any remaining gaps.

Therefore, we aimed (1) to identify, give a systematic overview of, and appraise currently available prediction models for the risk of hospitalization, LOS, and readmission in patients with CKD; (2) to externally validate any identified models in the target populations of hemodialysis (HD) patients, peritoneal dialysis (PD) patients, and advanced CKD not receiving dialysis (aCKD-ND) patients; and (3) to develop a new model in HD patients for the 1-year risk of hospitalization if no valid models were identified.

## Methods

The prespecified protocol is available at https://osf.io/e5phq/. Deviations including their rationale are available (Supplemental methods – Protocol deviations). We followed the Preferred Reporting Items for Systematic Reviews and Meta-Analyses statement^22^ and the Transparent Reporting of a multivariable prediction model for Individual Prognosis or Diagnosis guidelines^23^ (Supplemental materials – Checklists). This study was approved by the scientific committee of the Department of Clinical Epidemiology at the Leiden University Medical Center (A184). This study adheres to the Declaration of Helsinki.

### Systematic Review

We searched PubMed for studies published in English that aimed to predict the risk of hospitalization, hospital readmission, or LOS in patients with CKD. The search string was developed together with an experienced librarian (Supplemental methods – Search string). The search was performed on June 4, 2024. All identified studies were independently screened on title and abstract by 2 researchers (RJJ and JM), after which the remaining studies were independently screened for inclusion based on their full text by 2 researchers (RJJ and JM). In case of disagreements between reviewers, MvD served as arbitrator. References of the included studies were cross-referenced for further eligible studies. The inclusion criteria for studies were as follows: (1) the study must intentionally develop, externally validate, or update a multivariable prognostic prediction model; (2) the study population must consist of adults (aged ≥18 years) with CKD (estimated glomerular filtration rate [eGFR] <60 mL/min/1.73 m^2^) or adults undergoing maintenance dialysis; (3) the prediction model must predict hospital admission with a prediction horizon of 5 years or less, or LOS at the time of hospital admission; and (4) the data was sourced from a longitudinal cohort.

Data were extracted by 1 researcher (RJJ) following the Checklist for critical Appraisal and data extraction for systematic Reviews of prediction Modelling Studies.^24^ Additionally, risk of bias and applicability were assessed for all models using the Prediction model Risk of Bias Assessment Tool (PROBAST).^25^

### Data Sources

We used 2 data sources in this study. The first was the Netherlands Cooperative Study on the Adequacy of Dialysis (NECOSAD), which is a cohort study consisting of 2,045 incident dialysis patients included in 38 out of 49 dialysis centers in the Netherlands between 1997 and 2006.^26^ Follow-up was performed every 6 months for a maximum of 10 years.

The second was the European Quality (EQUAL) study, an international prospective cohort study including 1,738 Dutch, United Kingdom, German, Italian, Polish, and Swedish patients aged ≥65 years with an eGFR <20 mL/min/1.73 m^2^.^27^ Follow-up occurred every 6 months for up to 4 years. An additional follow-up was performed 3 months after dialysis initiation or when eGFR <10 mL/min/1.73 m^2^.

Both data sources contained information on individuals’ demographics (age, sex, educational level, etc.), physical examination (body mass index, blood pressure, etc.), medical history (comorbid conditions, summary scores), laboratory tests (eg, 24-hour urine, electrolytes, serum creatinine), medication use (medication list, frequency), health care access (hospital admissions and discharge, admission diagnoses, etc.), and disease-specific information (primary kidney disease, first nephrologist visit, etc.).

For both data sources, the medical ethical committees of participating centers approved data collection, and all patients provided written informed consent before inclusion.

### External Validation

For all models, if information needed for external validation (eg, intercept and regression coefficients) was unavailable, authors were contacted via email to request any remaining information needed. On no response, 1 reminder was sent. For models with only regression coefficients available (ie, intercept was not reported), we re-estimated the intercept in the validation data (Supplemental methods – Re-estimating intercepts).^28^ We validated the models in 3 target populations using our available data sources: HD patients (NECOSAD), PD patients (NECOSAD), and aCKD-ND patients (EQUAL). aCKD-ND patients that started receiving dialysis were censored at dialysis initiation.

Models were validated using calibration-in-the-large (the difference of the mean observed outcome and the mean predicted outcome; 0 is perfect), the calibration slope (the slope of the calibration line; 1 is perfect), and calibration plots. Calibration plots contained deciles of predicted versus observed risk and a locally estimated scatterplot smoother. Models with a binary outcome were additionally validated on their discrimination using the C-statistic (ranging from 0 to 1, with 0.5 being as good as flipping a coin and 1 being perfect discrimination). Validation of time-to-event models was performed while taking into account the competing risk of death.^29^^,^^30^ In calibration plots, censoring events and competing risks were plotted using jackknife pseudo-values (Supplemental methods **– Pseudo-values**).^31^ Discrimination was assessed with Wolbers’ adaptation for Harrell’s C-statistic.^32^

### Model Development

We prespecified that if no valid models were identified, we would develop a new model for the risk of hospitalization within 1 year after the first 3 months of dialysis. Hospitalizations for vascular access were excluded. Predictors were selected based on literature, availability, and clinical relevance as determined by a nephrologist (JIR). Taking into account the competing risk of death, the model was developed using a Fine-Gray model in the NECOSAD HD population. Individuals were censored at kidney transplantation, loss to follow-up, or at 1 year after baseline. Outcome assessment was independent of predictors. We evaluated apparent performance and performed targeted external validation in PD (NECOSAD) and aCKD-ND (EQUAL) patients as described in *External validation*. In aCKD-ND patients, the risk was estimated at a first eGFR measurement of 20 mL/min/1.73 m^2^. Moreover, we re-estimated the model in the target external validation populations if they did not perform well.

Based on model performance, we tried additional modeling strategies post hoc using logistic and Cox proportional hazards models to improve model performance. Additionally, one post hoc sensitivity analysis was performed to predict a hospitalization of ≥3 days. We believe that hospitalizations with longer LOS are a different type of hospitalization (eg, more severe, less for diagnostic purposes) and that predictors might influence this outcome differently than for shorter hospitalizations. Moreover, hospitalizations with longer LOS are more impactful for patients.

### Statistical Analysis

Continuous variables are presented as mean (standard deviation) or median [interquartile range] depending on their distribution. Categorical variables are presented as number (percentage). Missing data were imputed using multiple imputation with 20 imputations and 20 iterations (Supplemental methods – Missing data). For the developed model, collinearity between predictors was assessed using Spearman’s rank correlation coefficient. The linearity of continuous predictors was visually assessed using restricted cubic splines. The proportional hazards assumption was evaluated by plotting the regression coefficient of each variable as a function of time. Calibration slopes were calculated using a regression model equivalent to the development regression model (eg, Poisson regression for a Poisson model). Confidence intervals for C-statistics were calculated using 500 bootstraps. All statistical analyses were performed in R version 4.4.0 (R Foundation for Statistical Computing).

## Results

### Identified Models

**Figure 1:**
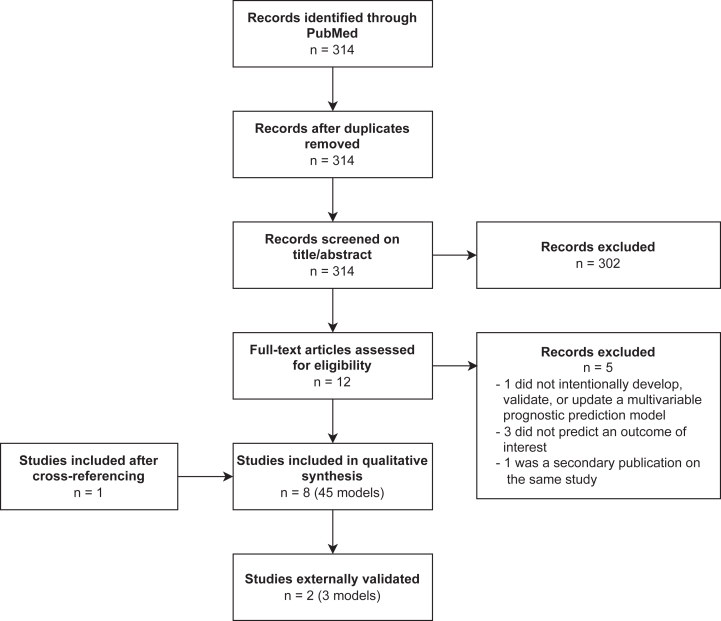
Flowchart showing inclusion of articles in the systematic review.

We identified 8 studies (Fig 1). Most studies developed multiple models (Table S1), of which 1 (eg, the best performing model) was deemed the primary model (Table 1).^33^, ^34^, ^35^, ^36^, ^37^, ^38^, ^39^, ^40^ All models were developed either with patients from the United States or from China, and 37 of the 45 models were developed in only 3 studies.^37^, ^38^, ^39^ Two models predicted hospitalization rate in incident PD patients,^33^ 2 models predicted 30-day readmission in maintenance HD patients,^35^ 27 models predicted 30-day readmission in CKD patients in general,^39^ and 1 model predicted 30-day readmission in CKD stage 3-5 patients not receiving dialysis who were discharged after a heart failure hospitalization^34^ (Table S2). One model predicted the risk of ≥1 hospitalization, and 1 model predicted the risk of ≥6 hospitalizations, both within 1 year in prevalent HD patients.^36^ The 90-day risk of hospitalization in CKD stage 3-5 patients not receiving dialysis was also predicted by 1 model.^40^ Prolonged LOS in PD patients was predicted by ten models.^37^^,^^38^

**Table 1:** Overview of General Characteristics of the Main Models in the Identified Studies

Study	Model	Country	Inclusion	Design	Population	Outcome	Follow-up Time	Prediction Horizon	Assistive or Decisive^a^
Fried et al^33^ (2003)	CCI	United States	January 1, 1990-January 2, 2000	Not-for-purpose cohort^b^	Incident maintenance PD	Hospitalization rate	0.5-10.5 y	—	Assistive
Fried et al^33^ (2003)	Davies	United States	January 1, 1990-January 1, 2000	Not-for-purpose cohort	Incident maintenance PD	Hospitalization rate	6 mo	—	Assistive
Perkins et al^34^ (2013)	—	United States	July 1, 2004-February 2, 2010	Not-for-purpose cohort	CKD patients stage 3-5 not receiving KRT discharged after HF hospitalization	Readmission within 30 d	1-69 mo	30 d	Assistive
Flythe et al^35^ (2016)	Admission	United States	May 1, 2008-June 27, 2013	Not-for-purpose cohort	Discharged maintenance HD	Readmission within 30 d	30 d	30 d	Assistive
Flythe et al^35^ (2016)	Discharge	United States	May 1, 2008-June 27, 2013	Not-for-purpose cohort	Discharged maintenance HD	Readmission within 30 d	30 d	30 d	Assistive
Wong et al^36^ (2020)	1 hospitalization	United States	January 1-14, 2012	Not-for-purpose cohort	Prevalent HD patients (>1 y of treatment)	Hospitalization	1 y	1 y	Assistive
Wong et al^36^ (2020)	6 hospitalizations	United States	January 1-14, 2012	Not-for-purpose cohort	Prevalent HD patients (>1 y of treatment)	≥6 hospitalizations	1 y	1 y	Assistive
Wu et al^37^ (2020)	Score	China	2013-2015	Not-for-purpose cohort	PD patients	Prolonged length of stay	—	To discharge	Assistive
Kong et al^38^ (2021)	Stacking	China	2013-2015	Not-for-purpose cohort	Admitted PD patients	Prolonged length of stay	—	To discharge	Assistive
Zhou et al^39^ (2022)	wFMFP with 5 wFMs	China	2008-2017	Not-for-purpose cohort	CKD patients	Readmission within 30 d	30 d	30 d	Assistive
Karpinski et al^40^ (2023)	—	United States	2017-2018	Not-for-purpose cohort	CKD patients stage 3-5 not receiving dialysis	Hospitalization	90 d	90 d	Assistive

Abbreviations: CCI, Charlson comorbidity index; CKD, chronic kidney disease; HD, hemodialysis; HF, heart failure; KRT, kidney replacement therapy; PD, peritoneal dialysis; wFM, weighted factorization machine; wFMFP, weighted factorization machine with fuzzy partition.

a A decisive model directs treatment based on a prespecified (and often protocolized) cutoff, whereas an informative model means to only provide information to the health care provider and/or patient.

b Not-for-purpose cohort indicates that data were used that was not specifically collected for developing the prediction model.

The models for prolonged LOS and 90-day hospitalization risk were predominantly developed using techniques generally considered machine learning, such as classification and regression trees and *K*-nearest neighbor (Table 2, Table S3).^33^, ^34^, ^35^, ^36^, ^37^, ^38^, ^39^, ^40^ Readmission models in general CKD patients were mainly developed using a weighted factorization machine. Other models were mainly developed using regression techniques (Poisson, logistic, generalized additive). Most models did not report on the model assumptions or predictor selection (Table S4). Reporting of model performance was limited to apparent performance or internal validation using bootstrapping or cross-validation. Although most models reported a C-statistic (if applicable), calibration plots were often missing. Instead, other performance measures were often used (Table S5). One model was presented as a risk score (ie, no coefficients were reported, but points per variable that on summation indicated a certain risk group).

**Table 2:** Modeling Characteristics of the Main Models in the Identified Studies

Study	Model	N	Events	Modeling Method	Candidate Predictors^a^	Predictor Selection Method	Penalization Method	Validation Method	CITL	Slope^b^	C-Statistic (95% CI)
Fried et al^33^ (2003)	CCI	415	—	Poisson regression	3	Lowest AIC from all possible models	None	None	*NR*	*NR*	*NA*
Fried et al^33^ (2003)	Davies	415	—	Poisson regression	4	Lowest AIC from all possible models	None	None	*NR*	*NR*	*NA*
Perkins et al^34^ (2013)	—	607	116	LR	23+	Univariable association of *P* value <0.1, then highest C-statistic and Hosmer-Lemeshow test per variable domain, then adding variables in a final model, then removing variables that did not affect performance	None	1,000 bootstraps	*NR*	*NR*	0.79 (0.75-0.84)
Flythe et al^35^ (2016)	Admission	349	112	LR	29	Univariable LR with *P* value <0.20, then backward selection with *P* value <0.10 and no missing data	Sensitivity analysis with LASSO	1,000 bootstraps	*NR*	*NR*	0.68 (0.60-0.77)
Flythe et al^35^ (2016)	Discharge	349	112	LR	52	Univariable LR with *P* value <0.20, then backward selection with *P* value <0.10 and no missing data	Sensitivity analysis with LASSO	1,000 bootstraps	*NR*	*NR*	0.79 (0.73-0.85)
Wong et al^36^ (2020)	1 hospitalization	13,892	4,017	GAM	31	Sequentially according to AIC	None	10× random split	*NR*	*NR*	0.70 (0.62-0.79)
Wong et al^36^ (2020)	6 hospitalizations	13,892	131	GAM	31	Sequentially according to AIC	None	10× random split	*NR*	*NR*	0.77 (0.70-0.84)
Wu et al^37^ (2020)	Score	22,859	5,754	LR	34	Predictors with *P* <0.05 in LR model	None	Random split (80% development, 20% validation)	*NR*	*NR*	*NR*
Kong et al^38^ (2021)	Stacking	23,992	7,270	Stacked prediction models (LR, KNN, SVM, RF)	15+	No selection	None	5-fold cross-validation	*NR*	*NR*	0.76 (0.75-0.77)
Zhou et al^39^ (2022)	wFMFP with 5 wFMs	418,305	392,863	wFMFP with 5 wFMs	10+	No selection	None	None	*NR*	*NR*	*NR*
Karpinski et al^40^ (2023)	—	40,000	4,174	GBDT	399	Model-inherent	None	Random split (80% development, 20% validation)	*NR*	*NR*	0.73

Abbreviations: AIC, Akaike information criterion; CCI, Charlson comorbidity index; CITL, calibration-in-the-large; GAM, generalized additive model; GBDT, gradient-boosted decision tree; KNN, *K*-nearest neighbor; LASSO, least absolute shrinkage and selection operator; LR, logistic regression; NA, not applicable; NR, not reported; RF, random forest; SVM, support vector machine; wFM, weighted factorization machine; wFMFP, weighted factorization machine with fuzzy partition.

a A plus sign indicates that only the lower bound was known. For example, 23+ means at least 23 candidate predictors, but possibly more.

b Calibration slope.

Of the 45 models, 7 reported coefficients needed to calculate the linear predictor. No models reported intercepts needed to transform the linear predictor into individual risks. After contacting the corresponding authors via email, 3 authors responded. For 4 models (2 studies), the required data were no longer available, while for 2 models (1 study), the models had been replaced by a set of new models that were not published and not available for external validation.

### Quality of Models

When appraising model quality, multiple limitations were identified (Table 3).^42^, ^43^, ^44^ The main limitations were the inadequate handling of missing data and excluding individuals based on future information (Table S6). Additionally, some models were developed in small samples. Continuous predictors were often dichotomized or used without any report on whether the linearity of the predictors was checked. These problems are reflected by the risk of bias results from the PROBAST (Table 4).^33^, ^34^, ^35^, ^36^, ^37^, ^38^, ^39^, ^40^ The PROBAST showed high risk of bias in the Participants domain, mainly due to exclusion of individuals based on future information, and in the Analysis domain, mainly due to inadequate handling of missing data, continuous variables, lack of performance assessment, and lack of adjustment for overfitting (Table S7). Applicability was adequate in all models as we had a broad review question.

**Table 3:** Overview of Shortcomings in Previous Prediction Models

Shortcoming	Explanation	Reference
Eligibility criteria based on future	Future information is not available at the point in time when the prediction model is intended to be used in clinical practice. This makes it unclear whether a patient matches the target population of the prediction model (for which the model’s performance was assessed) and thus whether the model can be reliably used for that patient.	Moons et al^42^
Inadequate handling of missing data, individuals with missing data often being excluded	If the reason for missing data is informative (eg, sicker individuals less often have laboratory data available and are more likely to be hospitalized), the population in which the model was developed will differ from the intended population. If a health care provider encounters a patient in clinical practice that has missing data, they can collect these missing data. However, this individual was not part of the population on which the model was developed. Therefore, it is uncertain whether the model will reliably perform in this individual as well.	Royston et al^43^
Data-driven model building	Data-driven model building increases the risk of overfitting, resulting in prediction models with lower performance in new patients.	Royston et al^43^
Categorization of continuous variables	Categorization leads to a loss of information and, in the case of categorization into ≥3 categories, requires estimation of additional coefficients, further increasing risk of overfitting.	Royston et al^43^
Not checking linearity for continuous variables	Regression coefficients often assume linearity between the predictor and the outcome. If a variable does not have a linear association with the outcome (eg, body mass index, which is likely to have a U-shaped association), the regression coefficient is an average of the effects and does not represent the true effect.	Royston et al^43^
Calibration not shown	Notwithstanding the importance of discriminative ability, it is important for a prediction model to give predictions that represent the true risks in the population. Without determining model calibration, this cannot be assessed.	Ramspek et al^44^
Underreporting of models	Individual risks cannot be calculated without the intercept and regression coefficients or software containing the prediction model. This impedes further validation efforts and actual implementation.	Ramspek et al^44^

**Table 4:** Risk of Bias and Applicability According to the PROBAST in Concise Format of the Main Models in the Identified Studies

Study	Model	ROB				Concerns Regarding Applicability				Overall	
		Participants	Predictors	Outcome	Analysis	Participants	Predictors	Outcome	Analysis	ROB	Applicability
Fried et al^33^ (2003)	CCI	Low	Low	Low	High	Low	Low	Low	Low	High	Low
Fried et al^33^ (2003)	Davies	Low	Low	Low	High	Low	Low	Low	Low	High	Low
Perkins et al^34^ (2013)	—	High	Low	Low	High	Low	Low	Low	Low	High	Low
Flythe et al^35^ (2016)	Admission	High	Low	Low	High	Low	Low	Low	Low	High	Low
Flythe et al^35^ (2016)	Discharge	Low	Low	Low	High	Low	Low	Low	Low	High	Low
Wong et al^36^ (2020)	1 hospitalization	High	Low	Low	High	Low	Low	Low	Low	High	Low
Wong et al^36^ (2020)	6 hospitalizations	High	Low	Low	High	Low	Low	Low	Low	High	Low
Wu et al^37^ (2020)	Score	High	Low	Low	High	Low	Low	Low	Low	High	Low
Kong et al^38^ (2021)	Stacking	High	Low	Low	High	Low	Low	Low	Low	High	Low
Zhou et al^39^ (2022)	wFMFP with 5 wFMs	Low	Low	Low	Unclear	Low	Low	Low	Low	Unclear	Low
Karpinski et al^40^ (2023)	—	Low	Low	Low	Low	Low	Low	Low	Low	Low	Low

Abbreviations: CCI, Charlson comorbidity index; ROB, risk of bias; wFM, weighted factorization machine; wFMFP, weighted factorization machine with fuzzy partition.

### External Validation

External validation of the models was strongly impeded by the underreporting of model information, predictors specific to select populations (eg, Chinese insurance type, affiliation with center where research was performed), and predictors that were not measured in our data sources (eg, nadir intradialytic systolic blood pressure). A list of all used predictors is available in Table S8. All external validations we were able to perform showed poor to low discrimination and calibration (Table S9, Figs S1-S3). Data comparisons and available full prediction models with re-estimated intercepts are available in Tables S10-S13.

### Model Development

**Figure 2:**
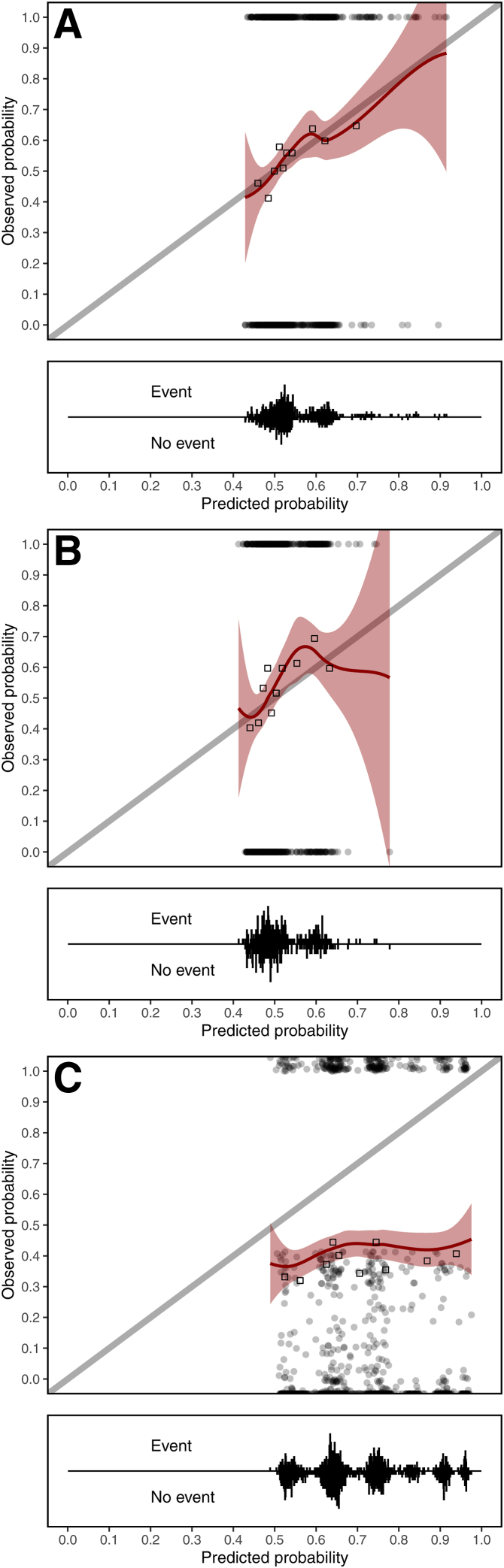
Calibration plots showing the predicted versus the observed probabilities of being hospitalized within 1 year. Plots are shown for the model in the development data (A), external validation data targeted at peritoneal dialysis patients (B), and external validation data targeted at patients with advanced chronic kidney disease who are not receiving dialysis (C).

We developed a model for the risk of being hospitalized within 1 year after starting dialysis in 1,020 incident HD patients (Table S14, Fig S4). The predictors age, cardiovascular disease, diabetes mellitus, malignancy, eGFR (before dialysis initiation), and body mass index were selected based on literature, availability, and clinical relevance (Table S15). In the development data, there were 557 hospitalizations, 51 deaths, and 412 censoring events. The modeling assumptions were sufficiently met (Table S16, Figs S5, S6). The apparent performance of the model showed decent calibration but unsatisfactory discrimination (Table 5, Fig 2A). When validating our model in 620 PD patients with 336 hospitalizations, performance was similar to that in the HD population, albeit slightly worse (Table 5, Fig 2B). In 1,718 aCKD-ND patients with 654 hospitalizations, the model was unfit to predict hospitalization risk (Table 5, Fig 2C).

**Table 5:** Performance Measures of the Developed Model in the Development Data, in the Validation Data, and in the Validation Data On Re-estimating in the Validation Data

Validation	N	Events	CITL	Calibration Slope	C-Statistic (95% CI)
Original model					
Fine-Gray					
Apparent	1,020	557	0.001	1.000	0.55 (0.53-0.58)
PD	620	336	0.027	0.929	0.55 (0.52-0.58)
aCKD-ND	1,718	654	−0.288	0.160	0.52 (0.50-0.54)
Cox PH					
Apparent	1,020	557	0.000	0.998	0.56 (0.53-0.58)
PD	620	336	0.024	0.890	0.55 (0.52-0.58)
aCKD-ND	1,718	654	−0.303	0.159	0.52 (0.50-0.54)
Logistic regression					
Apparent	1,020	557	0.000	1.018	0.58 (0.54-0.61)
PD	620	336	0.027	1.103	0.59 (0.54-0.64)
aCKD-ND	1,718	654	−0.330	0.107	0.52 (0.49-0.54)
Re-estimation of original model					
Fine-Gray					
PD	620	336	0.002	1.014	0.56 (0.53-0.59)
aCKD-ND	1,718	654	0.070	0.926	0.57 (0.55-0.60)
Sensitivity analysis: predicting hospitalization of ≥3 d					
Fine-Gray					
Apparent	1,020	209	0.000	1.000	0.58 (0.55-0.62)
PD	620	154	0.064	0.846	0.59 (0.54-0.63)
aCKD-ND	1,718	437	0.018	0.353	0.55 (0.52-0.57)
Cox PH					
Apparent	1,020	209	−0.001	1.002	0.59 (0.55-0.62)
PD	620	154	0.064	0.847	0.59 (0.54-0.63)
aCKD-ND	1,718	437	0.002	0.359	0.55 (0.52-0.57)
Logistic regression					
Apparent	1,020	209	0.000	1.000	0.59 (0.55-0.63)
PD	620	154	0.064	0.885	0.60 (0.55-0.66)
aCKD-ND	1,718	437	−0.004	0.276	0.54 (0.51-0.57)

Abbreviations: aCKD-ND, advanced chronic kidney disease not receiving dialysis; CI, confidence interval; CITL, calibration-in-the-large; PD, peritoneal dialysis.

For the post hoc Cox proportional hazards modeling and logistic regression modeling strategies, assumptions were also met (Figs S7-S9). The model performance was slightly better but remained unsatisfactory in apparent and external validations (Table 5, Figs S10-S11).

We also re-estimated the Fine-Gray model in the external validation datasets (Supplemental results – Re-estimating models). Model performance was improved compared to the external validations of the original model in these data, with decent calibration, but discrimination remained low (Table 5, Fig S12).

In the sensitivity analysis in which we changed the outcome to be predicted to hospitalization for ≥3 days, the model performed better across internal and external validations (Table 5), and models were better calibrated (Figs S13-S15). However, the C-statistic remained low for all models.

For completeness, the full model information for all developed models is in Table S17. The formulas for calculating individual risks are available in Supplemental results – Calculating individual risks.

## Discussion

In this study, we identified, appraised, and externally validated existing prediction models for hospitalization, LOS, and readmission in HD, PD, and aCKD-ND patients. We identified 45 models that had a multitude of shortcomings related to the data used, model development, and model assessment and presentation. Therefore, we developed a new model to predict hospitalization within 1 year after initiating HD. However, even when using sufficiently large data sets, clinically relevant predictors, and suitable methods, the model still performed unsatisfactorily. Subsequently, we made clinically and methodologically informed decisions to improve the model (eg, different modeling strategy), which had a marginal impact on model performance.

Our study provides an overview of the current status of risk prediction of hospitalization for patients with advanced CKD. Patients have requested more information about their risk of hospitalization,^9^^,^^10^ and predicting patient-relevant outcomes such as hospitalizations has also been stimulated internationally.^21^ Prediction models for hospitalization outcomes have the potential to be an important tool in personalized medicine and shared decision making. By providing patients with a personal indication of their risk of hospitalization, they will have a better idea of what to expect of the future, which can foster a feeling of control. This may be especially helpful for incident dialysis patients as they enter a new stage of their disease that is still largely unknown to them. Additionally, if a patient knows that they have a high risk of hospitalization, they may be able to cope better with the hospitalization if it occurs, as it is less unexpected.

No well-performing, validated, and methodologically sound models currently exist for predicting hospitalizations in patients with advanced CKD, but the present study allowed us to identify areas for improvement for future prediction models for hospitalization.

One area of improvement for future prediction models is the outcome definition. Predicting any reason for hospitalization likely results in excessive outcome heterogeneity. There are many reasons for hospitalization, including elective hospitalizations and short periods of hospitalization for diagnostic purposes. Predictors might differ in their predictive ability for different types of hospitalizations, leading to their coefficients not being able to simultaneously predict all these different types well. We tried to partially counteract this by excluding hospitalizations for vascular access, which are often planned. Additionally, we performed a sensitivity analysis predicting hospitalizations ≥3 days in length, which excludes the majority of hospitalizations for observation purposes. Although this slightly improved predictions, model performance remained unsatisfactory. Future prediction models for hospitalizations might reduce the heterogeneity in the outcome by only aiming to predict a subtype of hospitalizations, such as specifically predicting cardiovascular hospitalizations.

Another way of improving predictive performance is through predictor selection. We only included clinical predictors, such as cardiovascular disease. However, there is considerable residual risk likely attributable to more patient-centered factors. For example, patient frailty is likely to have a high influence on the risk of hospitalization and may therefore be highly informative. Although frailty is difficult to capture, patient-reported outcome measures do capture part of patient-specific wellbeing, such as health-related quality of life and symptom burden. By incorporating patient-reported outcome measures, performance might be improved. Additionally, hospitalizations are partially influenced by time-varying factors, such as intercurrent illnesses. Taking these into account in a dynamic approach to predicting hospitalizations might improve predictive performance.

Specifically for aCKD-ND, predictive performance might also be improved by a development process (eg, predictor selection, outcome specification) tailored to the aCKD-ND population. Our model was developed for patients receiving HD and validated and re-estimated in individuals with aCKD-ND. Although re-estimation showed slight improvement in performance, the aCKD-ND population might differ too much in terms of baseline risk and relevant predictors from a dialysis population to achieve adequate predictions using a model developed for patients on HD. A prediction model developed specifically for aCKD-ND might have better predictive performance.

The heterogeneity between different health care systems and countries is noteworthy when predicting hospitalizations. We accounted for this by allowing re-estimation of the developed prediction models to finetune the models to the health care system in which they were to be used. However, as our models did not perform adequately enough, re-estimation of prediction models for hospitalization will require further attention once a good model is developed.

Considering these areas of improvement, data that contains relevant information is still required. Although our data sources had large sample sizes and granular data, we were missing some predictors we thought to be relevant (eg, previous hospitalizations). Other data sources containing this information are available, such as the Chronic Renal Insufficiency Cohort Study,^41^ which contains data on kidney measures, hospital admissions, and quality of life. Using our results in combination with data such as the Chronic Renal Insufficiency Cohort Study will provide a good starting point for further improvement of hospitalization prediction in patients with CKD.

Our study has several strengths. We used a broad review question to identify prediction models for hospitalization, LOS, or readmission in our population. By extracting granular data from all models, we were able to get a broad overview of the current prediction models and their shortcomings. We then developed our model according to the current best practices with suitable methods. Additionally, we used data with a high number of events and were able to perform targeted external validations in 2 relevant populations. Finally, although our model underperformed, we were able to identify several lacunae that might improve the performance of future models if resolved. Nonetheless, our study also carries some limitations. We could not determine the reason for all hospitalizations, limiting us to predicting hospitalizations in general instead of a specific subset of hospitalizations. In addition, strategies for hospitalizations might have changed over time, leading to older data being less suited to modern-day clinical practice. However, our strategy of re-estimating underperforming models may alleviate this. Moreover, we did not have access to all relevant predictors, which might have improved predictions. Finally, cohort studies might not include individuals with CKD that are identified at a later stage in the disease process. These patients might have a higher risk of hospital admission, meaning that the model would work less well in all patients combined.

In conclusion, the risk of hospitalization for patients with advanced CKD is an important but complex outcome to predict. Current models are insufficient to predict hospitalizations. Therefore, we urge researchers to develop and validate better models, taking into account current shortcomings and our suggestions for improvement, to provide patients with adequate predictions of their risk of hospitalization.

## Supplementary Materials

Supplementary File (PDF) Supplemental methods; Figures S1-S15; Tables S1-S17; Supplemental Results, Checklists, Supplemental references.
