# Supplementary material for: Predicting Hospitalization and Related Outcomes in Advanced Chronic Kidney Disease: A Systematic Review, External Validation, and Development Study
Source: Kidney Med. 2025 Apr 25;7(7):101016. doi: 10.1016/j.xkme.2025.101016 (PMC12221738; doi:10.1016/j.xkme.2025.101016)
Supplement: Supplementary File (PDF) — Supplemental methods; Figures S1-S15; Tables S1-S17; Supplemental Results, Checklists, Supplemental references. [file mmc1.pdf]

Predicting hospitalisation and related outcomes in advanced chronic kidney disease: a systematic review, external validation, and development study

Supplemental materials

Roemer J. Janse, Jet Milders, Joris I. Rotmans, Fergus J. Caskey, Marie Evans, Claudia Torino, Maciej Szymczak, Christiane Drechsler, Christoph Wanner, Maria Pippias, Antonio Vilasi, Vianda S. Stel, Nicholas C. Chesnaye, Kitty J. Jager, Friedo W. Dekker, Merel van Diepen, and the EQUAL study investigators

Table of contents

Supplemental methods ____ 4

Protocol deviations ____ 4

Search string ____ 4

Re-estimating intercepts ____ 4

Pseudo-values ____ 5

Missing data ____ 6

Supplemental figures ____ 8

Figure S1. Calibration plots for the Charlson comorbidity index model from Fried *et al.*, validated in haemodialysis patients (**A**), peritoneal dialysis patients (**B**), and advanced chronic kidney disease not on dialysis patients (**C**). Circles indicate individual predicted rates and observed rates, while squares represent deciles of predicted rates and the mean observed rate in the decile. ____ 8

Figure S2. Calibration plots for the Davies model from Fried *et al.*, validated in haemodialysis patients (**A**) and peritoneal dialysis patients (**B**). Circles indicate individual predicted rates and observed rates, while squares represent deciles of predicted rates and the mean observed rate in the decile. ____ 9

Figure S3. Calibration plots for the admission model from Flythe *et al.*, validated in haemodialysis patients (**A**), peritoneal dialysis patients (**B**), and advanced chronic kidney disease not on dialysis patients (**C**). Circles indicate individual predicted rates and observed rates, while squares represent deciles of predicted rates and the mean observed rate in the decile. ____ 10

Figure S4. Flowchart of patient inclusion from NECOSAD for the development of the prediction model. ____ 11

Figure S5. Linearity assumption for the Fine-Gray model assessed with restricted cubic splines based on quantiles for the continuous predictors in the developed model: age (**A**), estimated glomerular filtration rate (**B**), and body mass index (**C**). ____ 12

Figure S6. Proportional hazards assumption for the Fine-Gray model for all variables in the developed model: age (**A**), cardiovascular disease (**B**), diabetes mellitus (**C**), malignancy (**D**), estimated glomerular filtration rate (**E**), and body mass index (**F**). ____ 13

Figure S7. Linearity assumption for the Cox proportional hazards model assessed with restricted cubic splines based on quantiles for the continuous predictors in the developed model: age (**A**), estimated glomerular filtration rate (**B**), and body mass index (**C**). ____ 14

Figure S8. Proportional hazards assumption for the Cox proportional hazards model for all variables in the developed model: age (**A**), cardiovascular disease (**B**), diabetes mellitus (**C**), malignancy (**D**), estimated glomerular filtration rate (**E**), and body mass index (**F**). ____ 15

Figure S9. Linearity assumption for the logistic regression model assessed with restricted cubic splines based on quantiles for the continuous predictors in the developed model: age (**A**), estimated glomerular filtration rate (**B**), and body mass index (**C**). ____ 16

Figure S10. Calibration plots showing the predicted vs. the observed probabilities of being hospitalised within one year for the Cox proportional hazards model internally validated in haemodialysis patients (**A**), externally validated targeting peritoneal dialysis patients (**B**), and externally validated targeting advanced chronic kidney disease not on dialysis patients (**C**). ____ 17

Figure S11. Calibration plots showing the predicted vs. the observed probabilities of being hospitalised within one year for the logistic regression model internally validated in haemodialysis patients (**A**), externally validated

targeting peritoneal dialysis patients (**B**), and externally validated targeting advanced chronic kidney disease not on dialysis patients (**C**). 18

Figure S12. Calibration plots showing the predicted vs. the observed probabilities of being hospitalised within one year for the model in the external validation data targeted at peritoneal dialysis patients (**A**) and the external validation data targeted at advanced chronic kidney disease not on dialysis patients (**B**) after the model was re-estimated in the validation data. 19

Figure S13. Calibration plots showing the predicted vs. the observed probabilities of being hospitalised for at least three days within one year for the Fine-Gray model internally validated in haemodialysis patients (**A**), externally validated targeting peritoneal dialysis patients (**B**), and externally validated targeting advanced chronic kidney disease not on dialysis patients (**C**). 20

Figure S14. Calibration plots showing the predicted vs. the observed probabilities of being hospitalised for at least three days within one year for the Cox proportional hazards model internally validated in haemodialysis patients (**A**), externally validated targeting peritoneal dialysis patients (**B**), and externally validated targeting advanced chronic kidney disease not on dialysis patients (**C**). 21

Figure S15. Calibration plots showing the predicted vs. the observed probabilities of being hospitalised for at least three days within one year for the logistic regression model internally validated in haemodialysis patients (**A**), externally validated targeting peritoneal dialysis patients (**B**), and externally validated targeting advanced chronic kidney disease not on dialysis patients (**C**). 22

Supplemental tables 23

Table S1. Overview of general characteristics of the models in the identified studies that were not the primary focus of the study. 23

Table S2. Information on the outcome of all identified models. 25

Table S3. Overview of general characteristics of the models in the identified studies that were not the primary focus of the study. 27

Table S4. Additional information on all identified models. 29

Table S5. Additional model performance measures reported for all identified models. 33

Table S6. Subjective interpretation of all identified models. 35

Table S7. Risk of bias and applicability according to the PROBAST (complete). 38

Table S8. Overview of predictors included in the identified prediction models. 40

Table S9. Performance metrics for models validated in haemodialysis patients (NECOSAD), peritoneal dialysis patients (NECOSAD) and advanced chronic kidney disease not on dialysis patients (EQUAL). 44

Table S10. Comparison of baseline characteristics between the development population of the Charlson comorbidity index model of Fried *et al.* and the external validation populations. 44

Table S11. Comparison of baseline characteristics between the development population of the Davies model of Fried *et al.* and the external validation populations. 45

Table S12. Comparison of baseline characteristics between the development population of the admission model of Flythe *et al.* and the external validation populations. 45

Table S13. Overview of the reported prediction formulas of identified studies, including intercepts re-estimated in our data. 46

Table S14. Baseline characteristics of individuals in the development and external validation data. 48

Table S15. Overview of predictors included in the developed model for the risk of hospitalisation within one year after initiating dialysis. 48

Table S16. Correlation between predictors as assessed with Spearman's rank correlation coefficient to assess collinearity in the developed model. 49

Table S17. Regression coefficients for all developed prediction models. To get individual predicted risks, see the explanation in Calculating individual risks. 49

Supplemental results ____ 50
Re-estimating models ____ 50
Calculating individual risks ____ 51
Checklists ____ 53
Preferred Reporting Items for Systematic Reviews and Meta-Analyses (PRISMA) ____ 53
Transparent reporting of a multivariable prediction model for individual prognosis or diagnosis (TRIPOD) statement ____ 55
Checklist for critical Appraisal and data extraction for systematic Reviews of prediction Modelling Studies (CHARMS) ____ 57
Supplemental references ____ 58

Supplemental methods

Protocol deviations

Deviation	Rationale
We did not validate continuous outcome with the R^2 as goodness of fit measure.	After consulting different literature sources, the R^2 seemed like a suboptimal measure that could not be readily calculated.
We did not perform the sensitivity analysis comparing different proxies for binary predictors.	Because many predictors were already unavailable, this sensitivity analysis was determined to not be relevant.
We tried two additional modelling strategies that were not pre-specified.	Because the developed model did not perform well, we experiment with different approaches to give the best recommendations for future research.

Search string

("kidney failure, chronic"[MeSH Major Topic] OR "chronic kidney"[Title] OR "chronic renal"[Title] OR "end stage renal"[Title] OR "endstage renal"[Title] OR "end stage kidney"[Title] OR "endstage kidney"[Title] OR "CKD"[Title/Abstract] OR "ESRD"[Title/Abstract] OR "ESKD"[Title/Abstract] OR "chronic nephropathy"[Title] OR "kidney chronic"[Title] OR "chronic renal insufficiency"[Title] **OR** ("Renal Dialysis"[MeSH Major Topic] OR "renal dialys*"[Title] OR "hemodialys*"[Title] OR "haemodialys*"[Title] OR "Hemodiafiltration"[Title] OR "Haemodiafiltration"[Title] OR "Continuous Ambulatory Peritoneal Dialysis"[Title] OR "CAPD"[Title/Abstract] OR (("renal"[Title] OR "kidney"[Title] OR "peritoneal"[Title]) AND ("Dialysis"[MeSH Terms] OR "dialys*"[Title])))) **AND** ("predictive model"[Title/Abstract] OR "predictive models"[Title/Abstract] OR "predictive model*"[Title/Abstract] OR "prediction model"[Title/Abstract] OR "prediction models"[Title/Abstract] OR "prediction model*"[Title/Abstract] OR "prediction rule"[Title/Abstract] OR "prediction rules"[Title/Abstract] OR "prediction tool"[Title/Abstract] OR "prediction tools"[Title/Abstract] OR "predictive rule"[Title/Abstract] OR "predictive rules"[Title/Abstract] OR "prognostic model"[Title/Abstract] OR "prognostic models"[Title/Abstract] OR "prognostic model*"[Title/Abstract] OR "risk score"[Title/Abstract] OR "risk scores"[Title/Abstract] OR "risk model"[Title/Abstract] OR "risk models"[Title/Abstract] OR "algorithms"[MeSH Major Topic] OR "algorithm"[Title/Abstract] OR "algorithms"[Title/Abstract] OR "scoring system"[Title/Abstract] OR "scoring systems"[Title/Abstract] OR "risk equation"[Title/Abstract] OR "risk equations"[Title/Abstract] OR "predict*"[Title] OR "predictive instrument"[Title/Abstract] OR "predictive instruments"[Title/Abstract] OR "risk index"[Title/Abstract] OR "risk indices"[Title/Abstract] OR "prognostic index"[Title/Abstract] OR "prognostic indices"[Title/Abstract] OR "prognostic score"[Title/Abstract] OR "prognostic scores"[Title/Abstract] OR "risk prediction"[Title/Abstract] OR "risk predictions"[Title/Abstract] OR "risk calculator"[Title/Abstract] OR "risk calculators"[Title/Abstract]) **AND** ("hospitalization"[MeSH] OR "patient readmission"[MeSH Major Topic] OR "length of stay"[Title/Abstract] OR "LOS"[Title/Abstract] OR "PLOS"[Title/Abstract])

Re-estimating intercepts

When prediction models developed using regression techniques had regression coefficients available but no intercept (β_0), we re-estimated the intercept by using an offset. We calculated each individual's linear predictor based on the available regression coefficients: $LP_i = \hat{\beta}_1 X_{1i} + \hat{\beta}_2 X_{2i} + \cdots + \hat{\beta}_k X_{ki}$. We then fit the same model as the original model used for the prediction model, using only the linear predictor with an offset, meaning the coefficient for the linear predictor is 1. For instance, for a logistic regression, this would then be $\log\left(\frac{p}{1-p}\right) = \beta_0 + LP$. We estimated this model for each imputation and take the mean of $\hat{\beta}_0$ as the intercept to validate the prediction model. In R, we did this using `offset()` from `{stats}`:

```
a <- mean(do.call("c", lapply(1:20, \(x){glm(outcome ~ offset(lp), family = "binomial",
    data = filter(dat_val, .imp == x))[["coefficients"]][["(Intercept)"]]})))
```

As the intercept in these models represents the outcome prevalence in the population, prediction models with a re-estimated intercept will likely have a calibration-in-the-large ($\frac{observed\ prevalence}{predicted\ prevalence}$) closer to 1 in the validation data.

Pseudo-values

To be able to plot a smoother and plot individual predictions in the calibration plot in the presence of censoring and competing events, we calculated jack-knife pseudo-values per individual i as $\tilde{Y}_i(t) = n\hat{F}_n(t) - (n-1)\hat{F}_n^{(i)}(t)$ where n is the number of individuals, $\hat{F}_n(t)$ is the Aalen-Johansen estimate at time t for hospitalisation (1), and $\hat{F}_n^{(i)}(t)$ is the Aalen-Johansen estimate at time t for hospitalisation excluding individual i (2) Instead of plotting the observed outcome, we then plotted the jack-knife values as observed outcome. We did this as follows:

Get Aalen-Johansen estimate for the outcome in the whole population:

```
library(dplyr)      # Data manipulation
library(magrittr)   # Better pipelines
library(survival)   # Survival analyses

# Get A-J estimate for outcome in total population
aj_tot <- survfit(Surv(tte, event, type = "mstate") ~ 1, data = df) %>%
   # Change fit list to dataframe
   tidy() %>%
   # Keep only events
   filter(state == "hospitalization") %>%
   # Remove everything after a year
   filter(time <= 365.25) %>%
   # Keep only last observation
   slice_tail(n = 1L) %>%
   # Keep estimate
   extract2("estimate")
```

Get Aalen-Johansen estimate for the outcome in a population excluding each individual once:

```
# Get A-J estimates for excluding each individual with jackknife
aj_in <- do.call("c", lapply(dat_tmp[["studynr"]], \(x){
   # Create new data
   dat_aj_tmp <- filter(df, studynr != x)

   # Calculate A-J estimate
   aj <- survfit(Surv(tte, event, type = "mstate") ~ 1, data = dat_aj_tmp) %>%
      # Change fit list to dataframe
      tidy() %>%
      # Keep only events
      dplyr::filter(state == "hospitalization") %>%
      # Remove everything after a year
      filter(time <= 365.25) %>%
      # Keep only last observation
      slice_tail(n = 1L) %>%
      # Keep estimate
      extract2("estimate")

   # Return estimate
   return(aj)
}))
```

Get jack-knife estimate of each individual based on the difference between the Aalen-Johansen estimate of the total population and the Aalen-Johansen estimate of the population without that individual:

```
# Get number of individuals
n <- nrow(dat_tmp)

# Get jackknife A-J estimate per individual
dat_tmp <- dat_tmp %>%
   # Calculate new variables
   mutate(# Total A-J estimate
          aj_t = aj_tot,
          # Jackknife A-J estimate
          aj_i = aj_in,
          # Pseudovalue for the outcome
          aj_o = (n * aj_t) - ((n - 1) * aj_i))

# Change observed to y from pseudovalues
y <- dat_tmp[["aj_o"]]
```

Missing data

Variables with missing data were imputed using multiple imputation with chained equations with 20 imputations and 20 iterations using `{mice}` in R. We performed multiple imputations six times: once each for data in NECOSAD on hospitalisation outcomes in HD, data in NECOSAD on hospitalisation outcomes in PD, data in NECOSAD on length of stay (LOS)/readmission outcomes in HD, data in NECOSAD on LOS/readmission outcomes in PD, data in EQUAL on hospitalisation outcomes, and data in EQUAL on LOS/readmission outcomes. Each dataset was imputed with variables in the table below (unless indicated with a dash [-]), which shows the percentage of missingness per variable per dataset. Additional to these variables, administrative variables that were complete for everyone, such as unique patient identifiers, visit numbers, and outcomes of interest, were used.

	Hospitalisation			Length of stay/readmission		
**Variable**	**NECOSAD HD**	**NECOSAD PD**	**EQUAL**	**NECOSAD HD**	**NECOSAD PD**	**EQUAL**
24-hour urine albumin	-	-	78.0%	-	-	76.2%
24-hour urine creatinine	34.5%	23.9%	66.4%	31.7%	21.6%	67.3%
24-hour urine protein	36.9%	28.2%	81.3%	33.9%	25.1%	86.0%
24-hour urine sodium	-	-	71.0%	-	-	72.1%
24-hour urine urea	44.5%	35.0%	70.0%	49.2%	36.8%	71.8%
24-hour urine volume	-	-	-	-	-	-
Access type	61.8%	-	-	78.6%	-	-
Acquired immunodeficiency syndrome	-	-	1.9%	-	-	2.0%
Admissions in the past half year	-	-	-	0.0%	0.0%	18.3%
Admissions in the past year	-	-	-	0.0%	0.0%	18.3%
Age	0.0%	0.0%	0.0%	0.0%	0.0%	0.4%
Albumin	23.4%	35.0%	23.0%	29.7%	38.5%	18.6%
Angina pectoris	0.1%	0.1%	3.5%	0.2%	0.3%	3.8%
Antithrombotics	-	-	0.0%	-	-	1.1%
At least one comorbidity	0.0%	0.0%	1.9%	0.0%	0.0%	2.0%
Atrial fibrillation	-	-	2.9%	-	-	2.8%
Beta blockers	0.0%	0.0%	0.0%	0.0%	0.0%	1.1%
Bicarbonate	30.0%	34.1%	39.7%	26.9%	27.7%	41.2%
Body Mass Index	15.1%	6.6%	6.9%	2.6%	2.0%	7.0%
Calcium	-	-	15.7%	-	-	13.2%
Calcium channel blockers	0.0%	0.0%	0.0%	0.0%	0.0%	1.1%
Cardiovascular disease	0.1%	0.1%	3.7%	-	-	-
Cerebrovascular disease	-	-	-	0.2%	0.3%	3.7%
Charlson comorbidity score	0.0%	0.0%	1.9%	0.0%	0.0%	2.0%
Cholesterol	-	-	43.6%	-	-	41.3%
Chronic lung disease	0.1%	0.1%	1.9%	0.2%	0.3%	2.0%
Coronary artery disease	0.1%	0.1%	3.7%	0.2%	0.3%	3.7%
Coumarin derivatives	14.0%	12.5%	-	0.5%	0.9%	-
Davies cerebrovascular disease	65.2%	65.9%	-	51.3%	50.2%	-
Davies diabetes mellitus	0.0%	0.0%	-	0.0%	0.0%	-
Davies heart failure	65.2%	65.9%	-	51.3%	50.2%	-
Davies ischemic heart disease	65.2%	65.9%	-	51.3%	50.2%	-
Davies malignancy	65.2%	65.9%	-	51.3%	50.2%	-
Davies other	65.2%	65.9%	-	51.3%	50.2%	-
Davies tissue disorder	0.1%	0.2%	-	0.2%	0.3%	-
Diabetes mellitus	0.0%	0.0%	2.0%	0.0%	0.0%	2.1%
Erythropoietin	0.8%	1.2%	4.6%	0.5%	0.8%	4.3%
Estimated glomerular filtration rate	13.3%	81.5%	28%	19.3%	80.8%	67.5%
Ethnicity	0.6%	7.7%	0.0%	0.3%	3.6%	1.1%
Heart failure	0.1%	0.1%	4.2%	0.2%	0.3%	3.1%
Height	-	-	6.5%	-	-	6.0%
Haematocrit	-	-	15.2%	-	-	11.0%
Haemoglobin	13.8%	14.2%	8.8%	24.1%	19.3%	6.2%
Human immunodeficiency virus	-	-	1.9%	-	-	2.0%
Hypertension	8.3%	22.0%	4.1%	1.2%	14.7%	3.2%
Iron medication	14.0%	12.5%	4.6%	0.5%	0.9%	4.3%
Karnofsky score	27.7%	25.9%	-	24.9%	19.7%	-
Left ventricular hypertrophy[a]	0.8%	1.0%	12.2%	0.4%	1.1%	8.8%
Lipid lowering agents	14.0%	12.5%	0.0%	0.5%	0.9%	1.1%
Liver cirrhosis	0.1%	0.1%	1.9%	0.2%	0.3%	2.0%
Malignancy	1.7%	1.0%	3.0%	1.5%	1.5%	3.2%

Mean corpuscular volume	-	-	14.0%	-	-	10.7%
Mineral supplements	-	-	0.0%	-	-	1.1%
Myocardial infarction	0.1%	0.1%	1.9%	0.2%	0.3%	2.0%
Other liver disease	-	-	1.9%	-	-	2.0%
Parathyroid hormone	-	-	34.9%	-	-	32.3%
Peripheral vascular disease	0.1%	0.1%	3.1%	0.1%	0.3%	2.6%
Phosphate	13.7%	14.1%	17.1%	24.1%	19.2%	14.1%
Potassium	-	-	8.8%	-	-	6.4%
Psychiatric disorder[a]	0.9%	1.0%	1.9%	0.5%	1.1%	2.0%
Renin-angiotensin-system inhibitors	0.0%	0.0%	0.0%	0.0%	0.0%	1.1%
Sex	0.0%	0.0%	0.0%	0.0%	0.0%	0.4%
Sodium	-	-	11.7%	-	-	8.2%
Surgery admitting service	-	-	-	0.0%	0.0%	0.4%
Systemic corticosteroids	44.5%	36.4%	0.0%	37.5%	31.0%	1.1%
Systolic blood pressure	4.6%	68.8%	11.5%	-	-	-
Systolic blood pressure at admission	-	-	-	42.3%	77%	94%
Therapy switch	13.4%	11.7%	-	0.0%	0.1%	-
Therapy switch within first year	0.4%	0.4%	-	-	-	-
Tissue disorder	0.1%	0.2%	1.9%	0.2%	0.3%	2.0%
Ulcer	-	-	1.9%	-	-	2.0%
Uric acid	-	-	43.6%	-	-	42.1%
Vitamin D supplements	-	-	4.6%	-	-	4.3%
Vitamin supplements	14.0%	12.5%	0.0%	0.5%	0.9%	1.1%
Weight	-	-	1.7%	-	-	2.6%

[a] Only available at baseline

Supplemental figures

Figure S1. Calibration plots for the Charlson comorbidity index model from Fried *et al.*, validated in haemodialysis patients (**A**), peritoneal dialysis patients (**B**), and advanced chronic kidney disease not on dialysis patients (**C**). Circles indicate individual predicted rates and observed rates, while squares represent deciles of predicted rates and the mean observed rate in the decile.

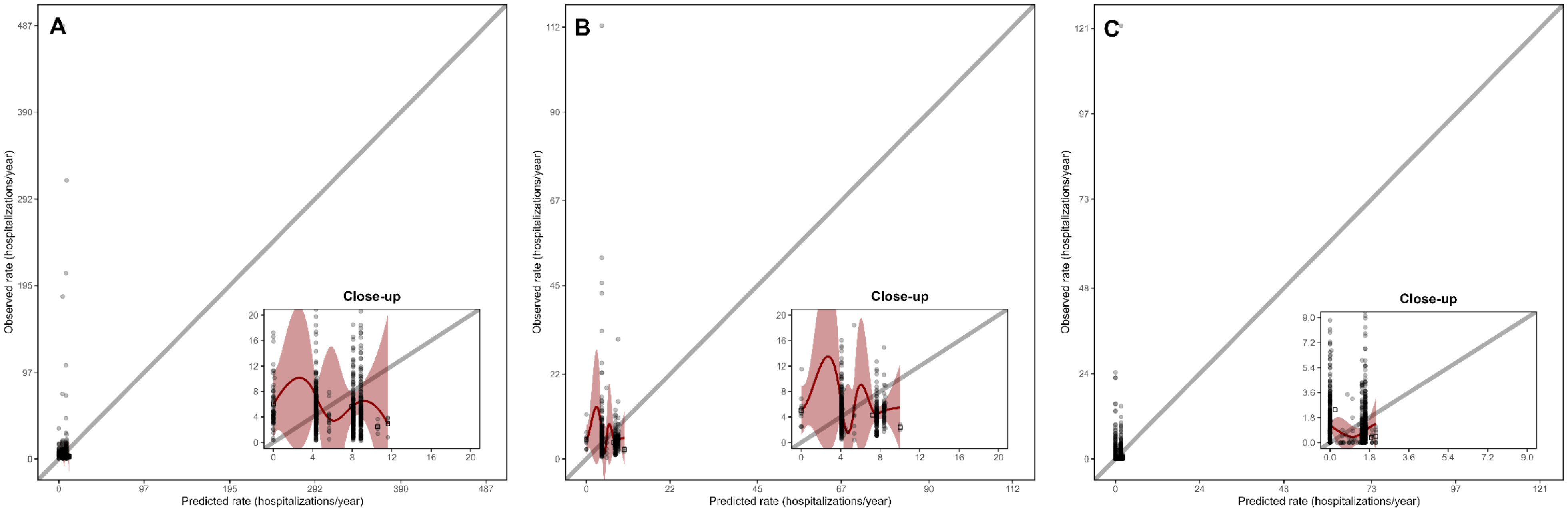

Figure S2. Calibration plots for the Davies model from Fried *et al.*, validated in haemodialysis patients (**A**) and peritoneal dialysis patients (**B**). Circles indicate individual predicted rates and observed rates, while squares represent deciles of predicted rates and the mean observed rate in the decile.

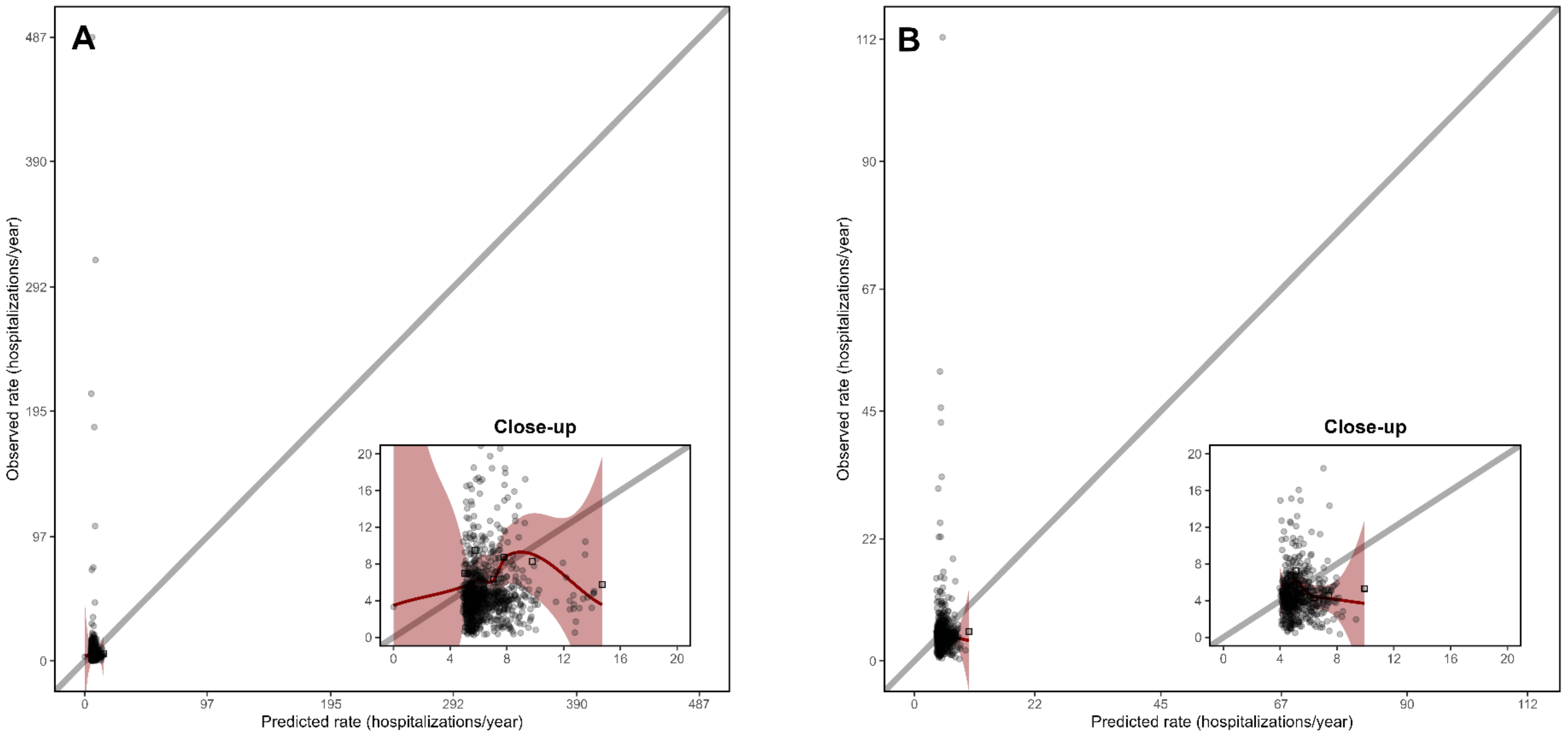

Figure S3. Calibration plots for the admission model from Flythe *et al.*, validated in haemodialysis patients (**A**), peritoneal dialysis patients (**B**), and advanced chronic kidney disease not on dialysis patients (**C**). Circles indicate individual predicted rates and observed rates, while squares represent deciles of predicted rates and the mean observed rate in the decile.

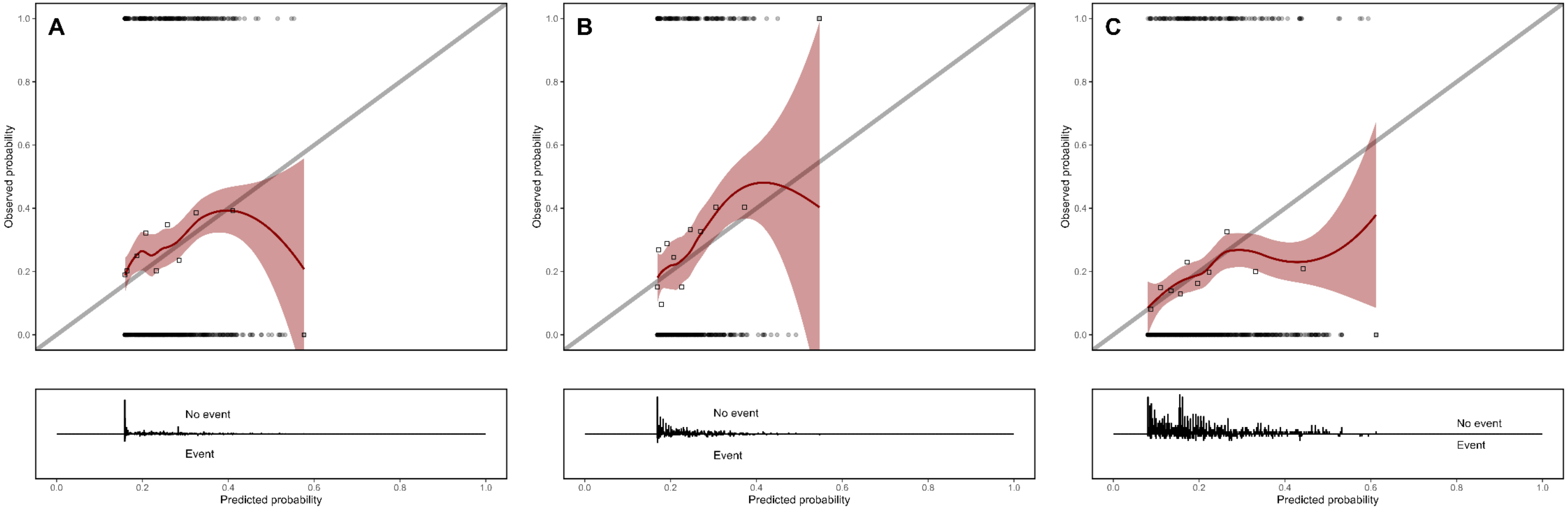

Figure S4. Flowchart of patient inclusion from NECOSAD for the development of the prediction model.

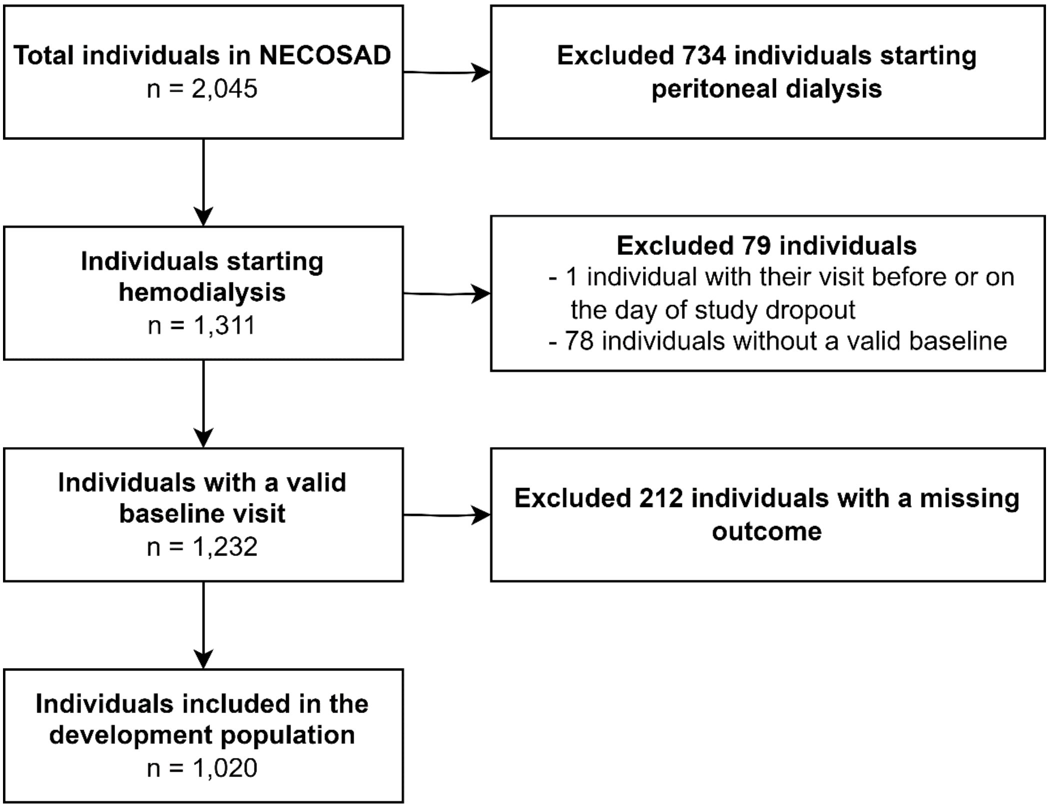

Figure S5. Linearity assumption for the Fine-Gray model assessed with restricted cubic splines based on quantiles for the continuous predictors in the developed model: age (**A**), estimated glomerular filtration rate (**B**), and body mass index (**C**).

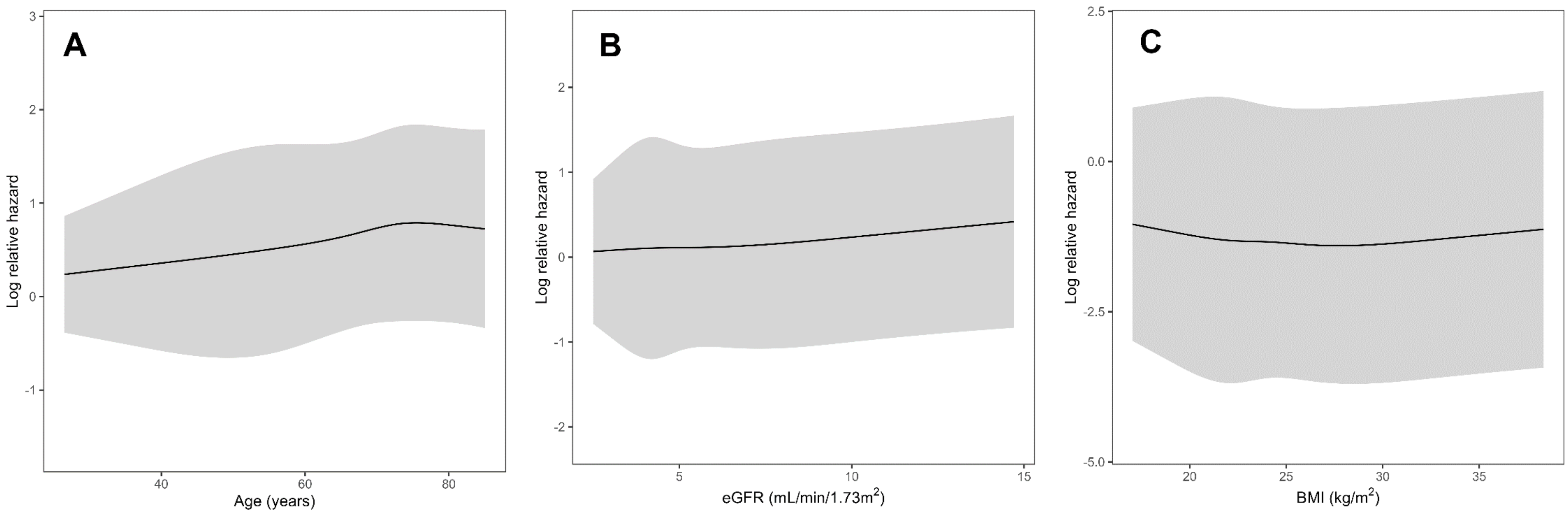

Figure S6. Proportional hazards assumption for the Fine-Gray model for all variables in the developed model: age (**A**), cardiovascular disease (**B**), diabetes mellitus (**C**), malignancy (**D**), estimated glomerular filtration rate (**E**), and body mass index (**F**).

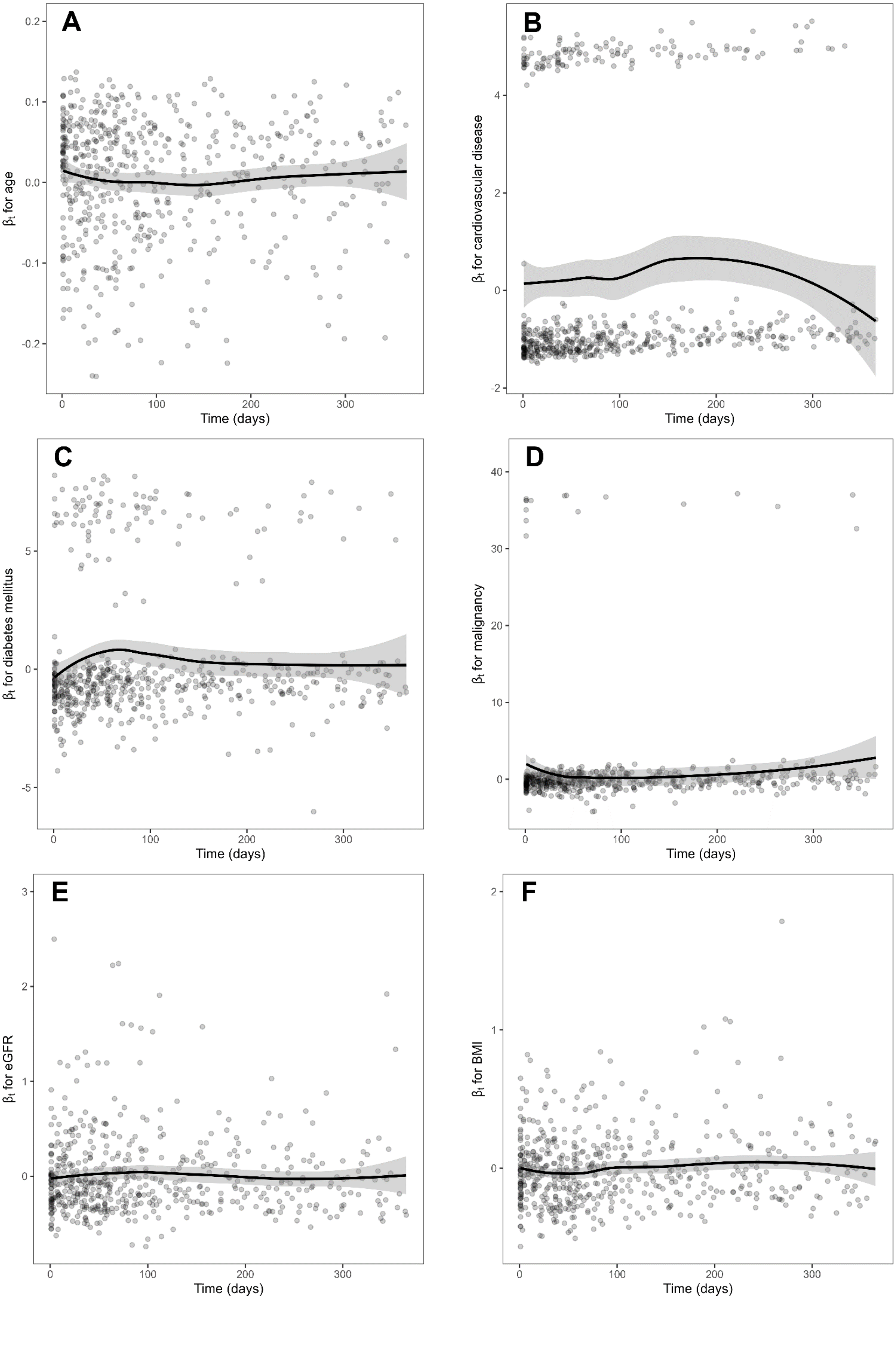

Figure S7. Linearity assumption for the Cox proportional hazards model assessed with restricted cubic splines based on quantiles for the continuous predictors in the developed model: age (**A**), estimated glomerular filtration rate (**B**), and body mass index (**C**).

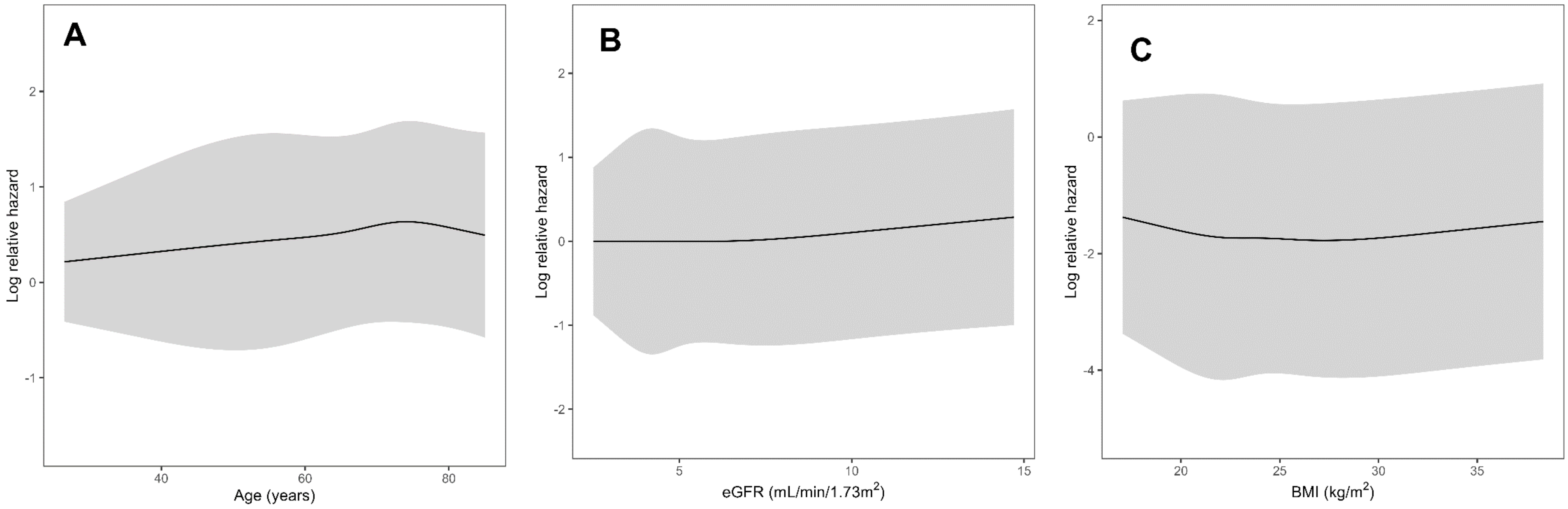

Figure S8. Proportional hazards assumption for the Cox proportional hazards model for all variables in the developed model: age (**A**), cardiovascular disease (**B**), diabetes mellitus (**C**), malignancy (**D**), estimated glomerular filtration rate (**E**), and body mass index (**F**).

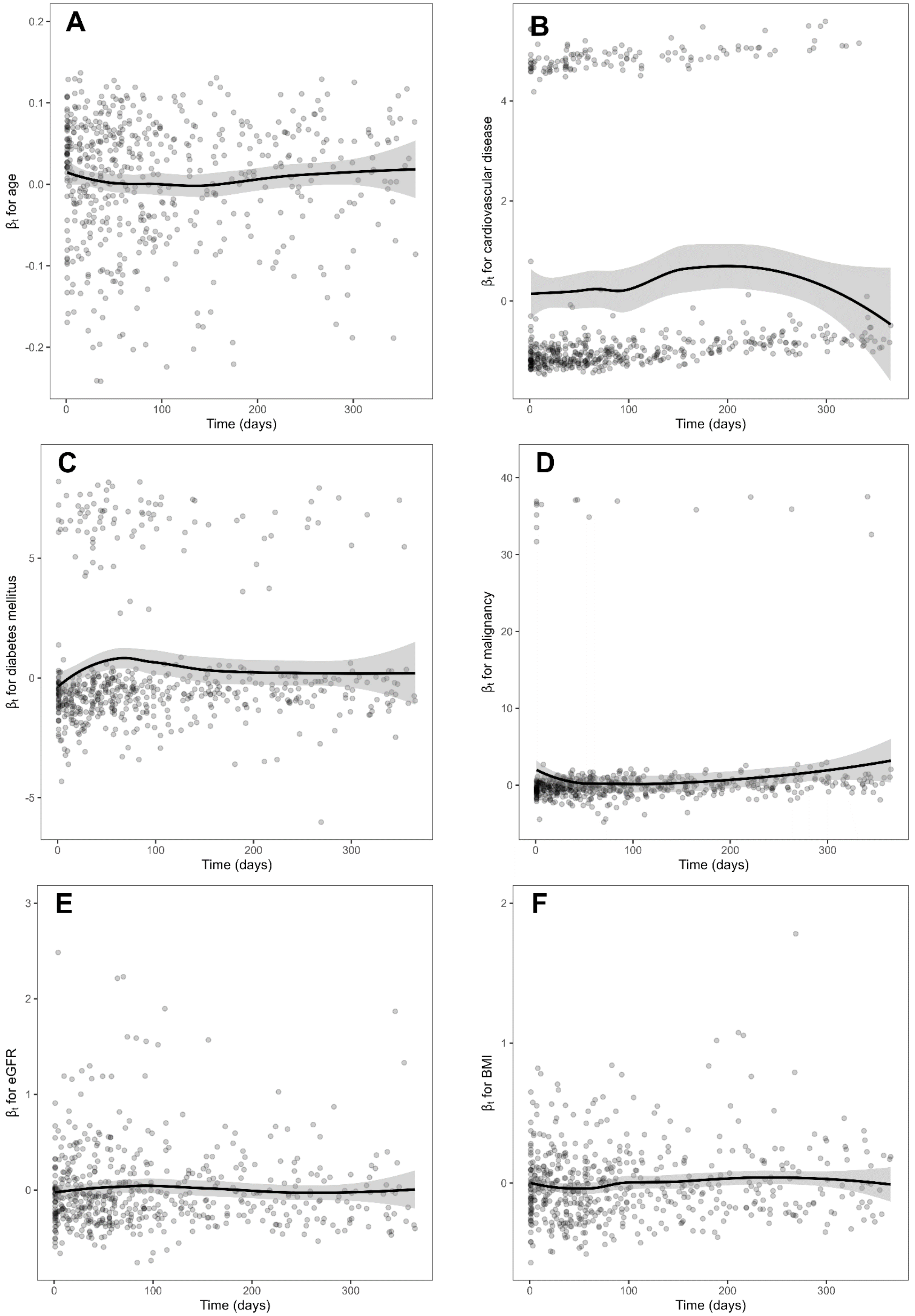

Figure S9. Linearity assumption for the logistic regression model assessed with restricted cubic splines based on quantiles for the continuous predictors in the developed model: age (**A**), estimated glomerular filtration rate (**B**), and body mass index (**C**).

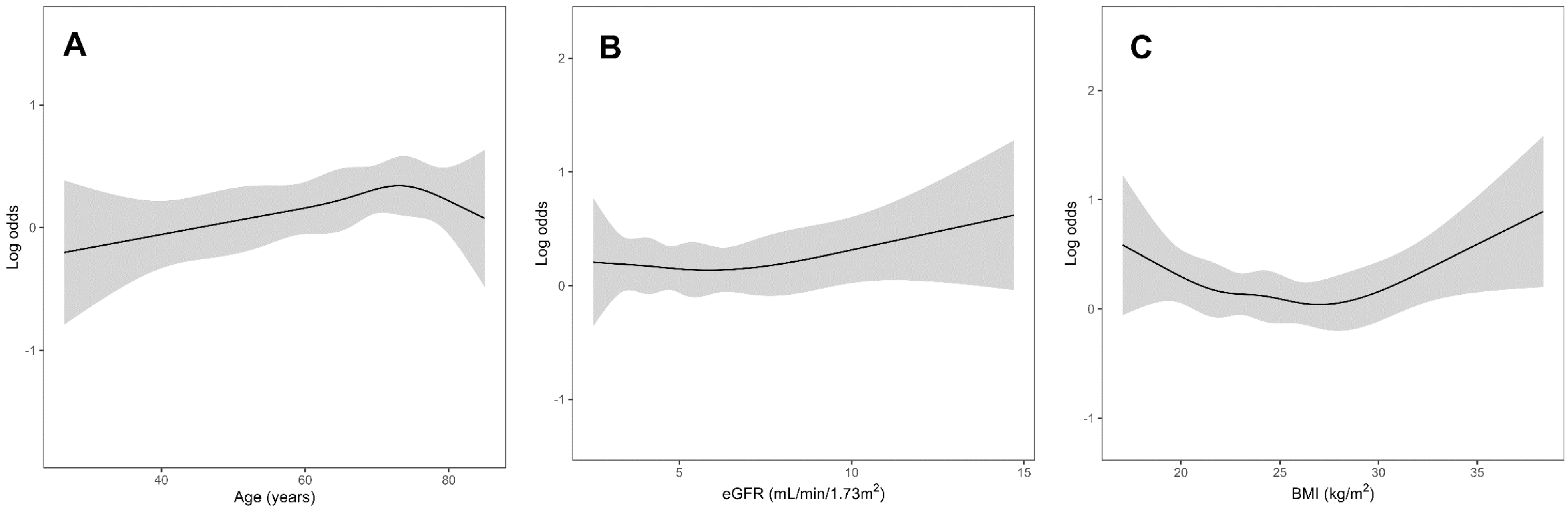

BMI was considered non-linear. Natural cubic splines were fitted in the logistic regression model with a knot at 27 kg/m^2. However, this did not considerably change the coefficient(s) for BMI nor influence model performance. For the sake of model simplicity, the natural cubic spline was therefore left out of the final logistic regression model.

Figure S10. Calibration plots showing the predicted vs. the observed probabilities of being hospitalised within one year for the Cox proportional hazards model internally validated in haemodialysis patients (**A**), externally validated targeting peritoneal dialysis patients (**B**), and externally validated targeting advanced chronic kidney disease not on dialysis patients (**C**).

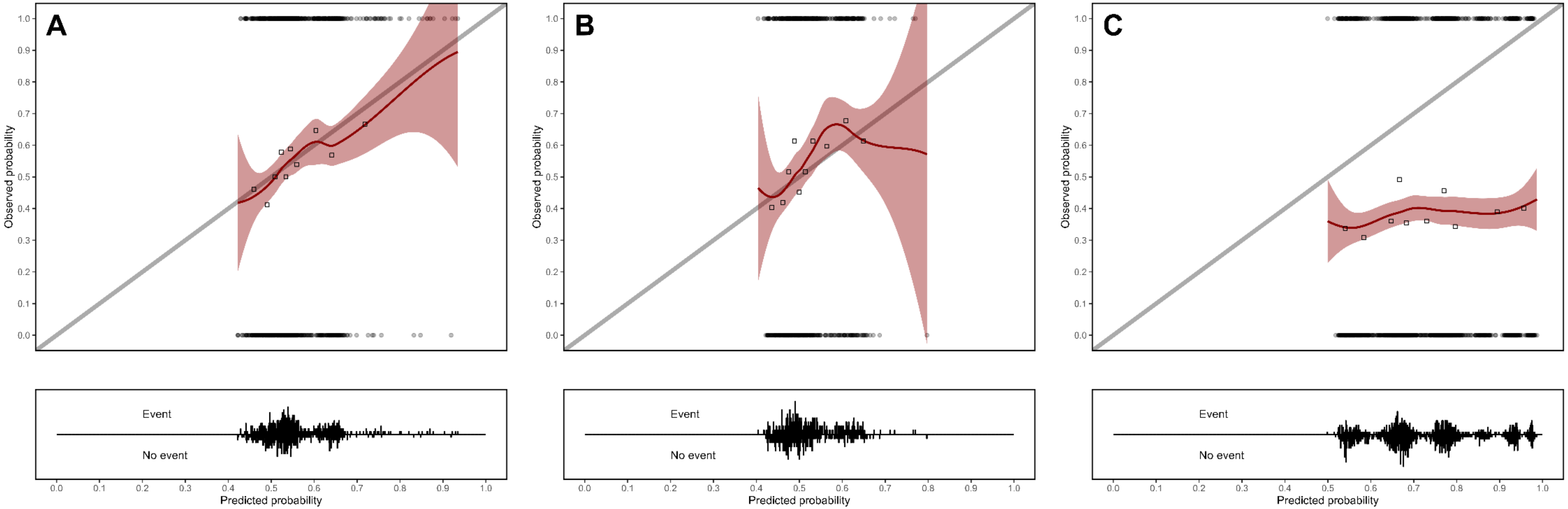

Figure S11. Calibration plots showing the predicted vs. the observed probabilities of being hospitalised within one year for the logistic regression model internally validated in haemodialysis patients (**A**), externally validated targeting peritoneal dialysis patients (**B**), and externally validated targeting advanced chronic kidney disease not on dialysis patients (**C**).

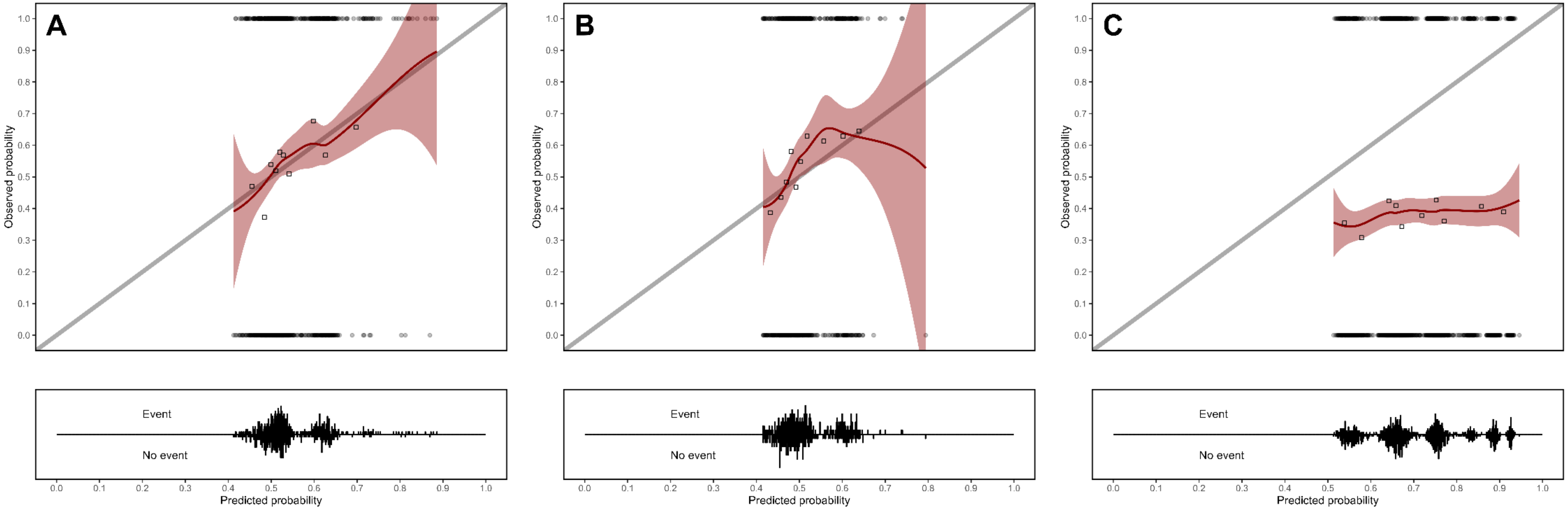

Figure S12. Calibration plots showing the predicted vs. the observed probabilities of being hospitalised within one year for the model in the external validation data targeted at peritoneal dialysis patients (**A**) and the external validation data targeted at advanced chronic kidney disease not on dialysis patients (**B**) after the model was re-estimated in the validation data.

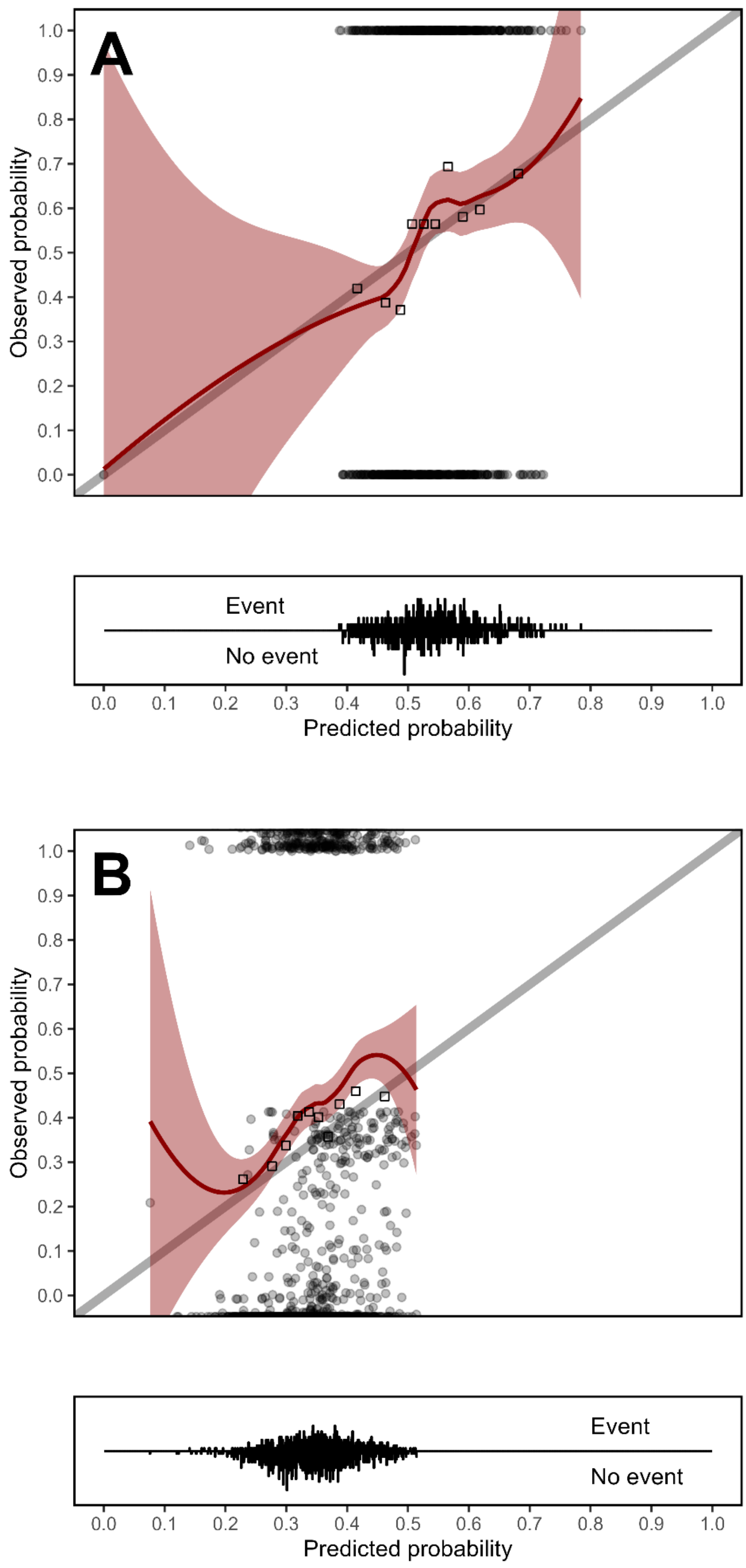

Figure S13. Calibration plots showing the predicted vs. the observed probabilities of being hospitalised for at least three days within one year for the Fine-Gray model internally validated in haemodialysis patients (**A**), externally validated targeting peritoneal dialysis patients (**B**), and externally validated targeting advanced chronic kidney disease not on dialysis patients (**C**).

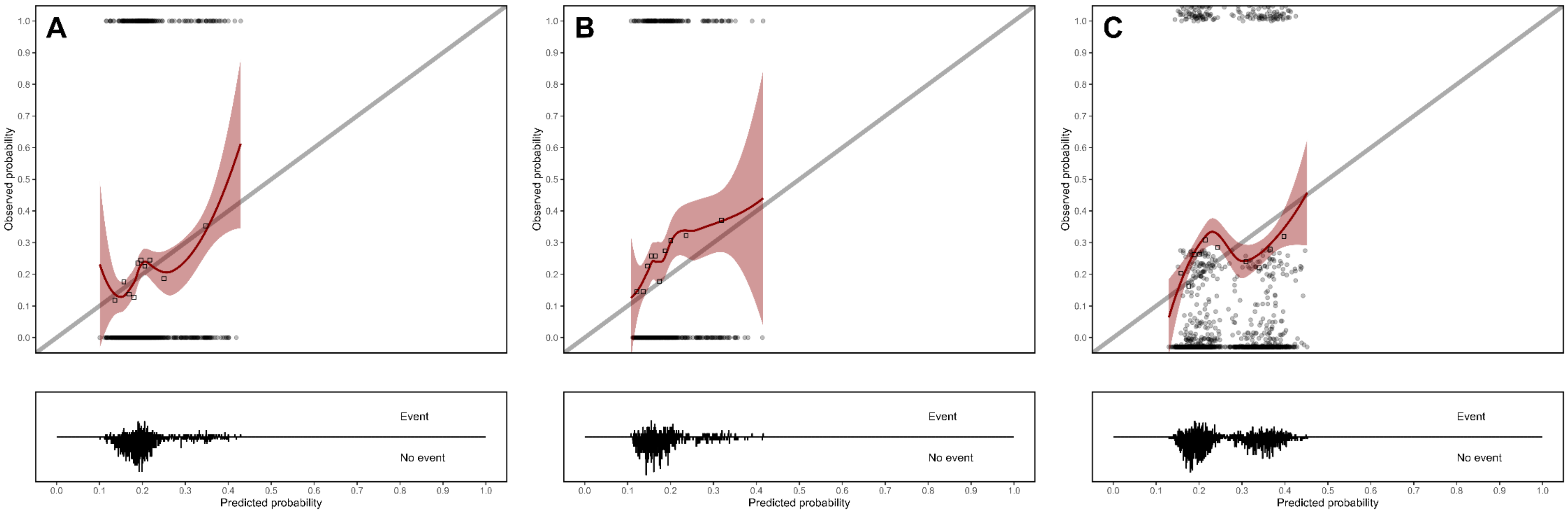

Figure S14. Calibration plots showing the predicted vs. the observed probabilities of being hospitalised for at least three days within one year for the Cox proportional hazards model internally validated in haemodialysis patients (**A**), externally validated targeting peritoneal dialysis patients (**B**), and externally validated targeting advanced chronic kidney disease not on dialysis patients (**C**).

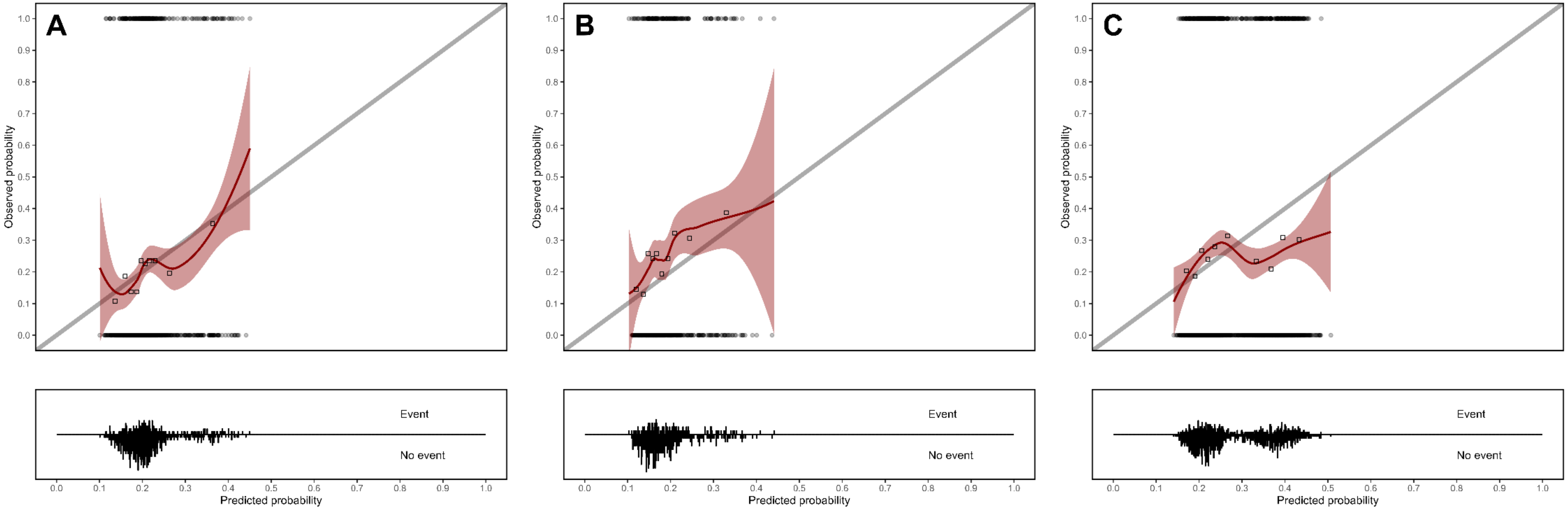

Figure S15. Calibration plots showing the predicted vs. the observed probabilities of being hospitalised for at least three days within one year for the logistic regression model internally validated in haemodialysis patients (**A**), externally validated targeting peritoneal dialysis patients (**B**), and externally validated targeting advanced chronic kidney disease not on dialysis patients (**C**).

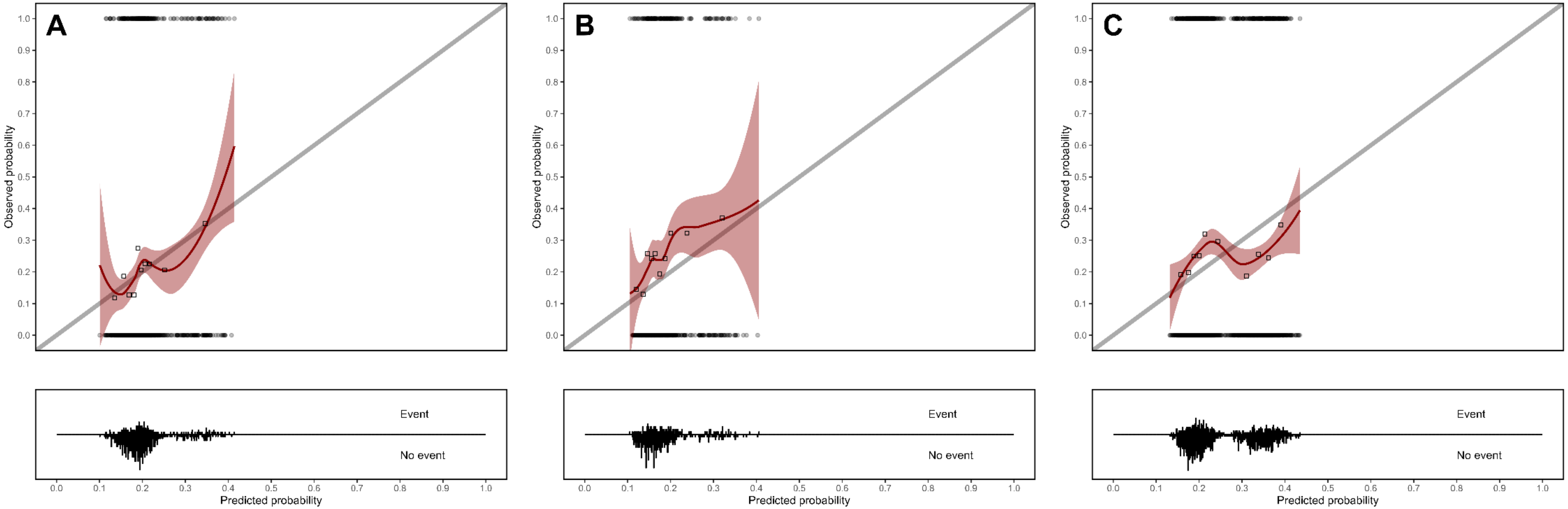

Supplemental tables

Table S1. Overview of general characteristics of the models in the identified studies that were not the primary focus of the study.

Study	Model	Country	Inclusion	Design	Population	Outcome	Follow-up time	Prediction horizon	Assistive or Decisive[a]
2020 Wu	CART	China	2013 to 2015	Not-for-purpose cohort[b]	PD patients	Prolonged length of stay	-	To discharge	Assistive
2020 Wu	GBDT	China	2013 to 2015	Not-for-purpose cohort	PD patients	Prolonged length of stay	-	To discharge	Assistive
2020 Wu	LR	China	2013 to 2015	Not-for-purpose cohort	PD patients	Prolonged length of stay	-	To discharge	Assistive
2020 Wu	RF	China	2013 to 2015	Not-for-purpose cohort	PD patients	Prolonged length of stay	-	To discharge	Assistive
2021 Kong	KNN	China	2013 to 2015	Not-for-purpose cohort	Admitted PD patients	Prolonged length of stay	-	To discharge	Assistive
2021 Kong	LR	China	2013 to 2015	Not-for-purpose cohort	Admitted PD patients	Prolonged length of stay	-	To discharge	Assistive
2021 Kong	RF	China	2013 to 2015	Not-for-purpose cohort	Admitted PD patients	Prolonged length of stay	-	To discharge	Assistive
2021 Kong	SVM	China	2013 to 2015	Not-for-purpose cohort	Admitted PD patients	Prolonged length of stay	-	To discharge	Assistive
2022 Zhou	wFMFP with 3 wFMs	China	2008 to 2017	Not-for-purpose cohort	CKD patients	Readmission within 30 days	30 days	30 days	Assistive
2022 Zhou	wFMFP with 4 wFMs	China	2008 to 2017	Not-for-purpose cohort	CKD patients	Readmission within 30 days	30 days	30 days	Assistive
2022 Zhou	wFMFP with 6 wFMs	China	2008 to 2017	Not-for-purpose cohort	CKD patients	Readmission within 30 days	30 days	30 days	Assistive
2022 Zhou	wFMFP with 7 wFMs	China	2008 to 2017	Not-for-purpose cohort	CKD patients	Readmission within 30 days	30 days	30 days	Assistive
2022 Zhou	wFMFP with 8 wFMs	China	2008 to 2017	Not-for-purpose cohort	CKD patients	Readmission within 30 days	30 days	30 days	Assistive
2022 Zhou	wFMFP with 9 wFMs	China	2008 to 2017	Not-for-purpose cohort	CKD patients	Readmission within 30 days	30 days	30 days	Assistive
2022 Zhou	wFMFP with 10 wFMs	China	2008 to 2017	Not-for-purpose cohort	CKD patients	Readmission within 30 days	30 days	30 days	Assistive
2022 Zhou	FMFP with 3 FMs	China	2008 to 2017	Not-for-purpose cohort	CKD patients	Readmission within 30 days	30 days	30 days	Assistive
2022 Zhou	FMFP with 4 FMs	China	2008 to 2017	Not-for-purpose cohort	CKD patients	Readmission within 30 days	30 days	30 days	Assistive
2022 Zhou	FMFP with 5 FMs	China	2008 to 2017	Not-for-purpose cohort	CKD patients	Readmission within 30 days	30 days	30 days	Assistive
2022 Zhou	FMFP with 6 FMs	China	2008 to 2017	Not-for-purpose cohort	CKD patients	Readmission within 30 days	30 days	30 days	Assistive
2022 Zhou	FMFP with 7 FMs	China	2008 to 2017	Not-for-purpose cohort	CKD patients	Readmission within 30 days	30 days	30 days	Assistive
2022 Zhou	FMFP with 8 FMs	China	2008 to 2017	Not-for-purpose cohort	CKD patients	Readmission within 30 days	30 days	30 days	Assistive
2022 Zhou	FMFP with 9 FMs	China	2008 to 2017	Not-for-purpose cohort	CKD patients	Readmission within 30 days	30 days	30 days	Assistive
2022 Zhou	FMFP with 10 FMs	China	2008 to 2017	Not-for-purpose cohort	CKD patients	Readmission within 30 days	30 days	30 days	Assistive
2022 Zhou	wFM	China	2008 to 2017	Not-for-purpose cohort	CKD patients	Readmission within 30 days	30 days	30 days	Assistive
2022 Zhou	FM	China	2008 to 2017	Not-for-purpose cohort	CKD patients	Readmission within 30 days	30 days	30 days	Assistive
2022 Zhou	SVM-Poly	China	2008 to 2017	Not-for-purpose cohort	CKD patients	Readmission within 30 days	30 days	30 days	Assistive
2022 Zhou	SVM-Sigmoid	China	2008 to 2017	Not-for-purpose cohort	CKD patients	Readmission within 30 days	30 days	30 days	Assistive
2022 Zhou	SVM-RBF	China	2008 to 2017	Not-for-purpose cohort	CKD patients	Readmission within 30 days	30 days	30 days	Assistive
2022 Zhou	MLP	China	2008 to 2017	Not-for-purpose cohort	CKD patients	Readmission within 30 days	30 days	30 days	Assistive
2022 Zhou	XGBoost	China	2008 to 2017	Not-for-purpose cohort	CKD patients	Readmission within 30 days	30 days	30 days	Assistive
2022 Zhou	Lightgbm	China	2008 to 2017	Not-for-purpose cohort	CKD patients	Readmission within 30 days	30 days	30 days	Assistive
2022 Zhou	Catboost	China	2008 to 2017	Not-for-purpose cohort	CKD patients	Readmission within 30 days	30 days	30 days	Assistive
2022 Zhou	GBM	China	2008 to 2017	Not-for-purpose cohort	CKD patients	Readmission within 30 days	30 days	30 days	Assistive
2022 Zhou	RF	China	2008 to 2017	Not-for-purpose cohort	CKD patients	Readmission within 30 days	30 days	30 days	Assistive

PD, peritoneal dialysis; CKD, chronic kidney disease; CART, classification and regression trees; GBDT, gradient boosted decision trees; LR, logistic regression; RF, random forest; KNN, K-nearest neighbour; SVM, support vector machine; wFMFP, weighted factorisation machine with fuzzy partition; wFM, weighted factorisation machine; FMFP, factorisation machine with fuzzy partition; FM, factorisation machine; RBF, radial basis function; GBM, gradient boosting machine.

[a] A decisive model directs treatment based on a pre-specified (and often protocolized) cut-off, whilst an informative model means to only provide information to the healthcare provider and/or patient.
[b] Not-for-purpose cohort indicates that data was used that was not specifically collected for developing the prediction model.

Table S2. Information on the outcome of all identified models.

Study	Model	Definition	Measurement[a]	Consis-tent[b]	Type[c]	Blinded assessment[d]	Incorpo-ration bias[e]	EPV
2003 Fried	CCI	Hospitalisations on PD divided by time on PD	Register	Yes	Single	No	No	*NA*
2003 Fried	Davies	*Idem ditto*	Register	Yes	Single	No	No	*NA*
2013 Perkins	-	Readmission to study medical centres within 30 days of index date	Register	Yes	Single	No	No	5
2016 Flythe	Admission	Rehospitalisation at UNC hospitals within 30 days of index discharge, excluding scheduled readmissions for vascular access or other planned procedures.	Not reported	Yes	Single	No	No	3.9
2016 Flythe	Discharge	*Idem ditto*	Not reported	Yes	Single	No	No	2.2
2020 Wong	1 hospitalisation	Hospitalisation	Register	Yes	Single	No	No	129.6
2020 Wong	6 hospitalisations	Six or more hospitalisations	Register	Yes	Single	No	No	4.2
2020 Wu	CART	Consecutive stay in hospital >16 days	Register	Yes	Single	No	No	169.2
2020 Wu	GBDT	*Idem ditto*	Register	Yes	Single	No	No	169.2
2020 Wu	LR	*Idem ditto*	Register	Yes	Single	No	No	169.2
2020 Wu	RF	*Idem ditto*	Register	Yes	Single	No	No	169.2
2020 Wu	Score	*Idem ditto*	Register	Yes	Single	No	No	169.2
2021 Kong	KNN	Consecutive stay in hospital (counting discharge and admission on same day as continuous admission) >16 days	Register	Yes	Single	No	No	484.7
2021 Kong	LR	*Idem ditto*	Register	Yes	Single	No	No	484.7
2021 Kong	RF	*Idem ditto*	Register	Yes	Single	No	No	484.7
2021 Kong	SVM	*Idem ditto*	Register	Yes	Single	No	No	484.7
2021 Kong	Stacking	*Idem ditto*	Register	Yes	Single	No	No	484.7
2022 Zhou	wFMFP with 3 wFMs	Admission record within 30 days of previous discharge to home	EHR data	Yes	Single	No	No	39,286.3
2022 Zhou	wFMFP with 4 wFMs	*Idem ditto*	EHR data	Yes	Single	No	No	39,286.3
2022 Zhou	wFMFP with 5 wFMs	*Idem ditto*	EHR data	Yes	Single	No	No	39,286.3
2022 Zhou	wFMFP with 6 wFMs	*Idem ditto*	EHR data	Yes	Single	No	No	39,286.3
2022 Zhou	wFMFP with 7 wFMs	*Idem ditto*	EHR data	Yes	Single	No	No	39,286.3
2022 Zhou	wFMFP with 8 wFMs	*Idem ditto*	EHR data	Yes	Single	No	No	39,286.3
2022 Zhou	wFMFP with 9 wFMs	*Idem ditto*	EHR data	Yes	Single	No	No	39,286.3
2022 Zhou	wFMFP with 10 wFMs	*Idem ditto*	EHR data	Yes	Single	No	No	39,286.3
2022 Zhou	FMFP with 3 FMs	*Idem ditto*	EHR data	Yes	Single	No	No	39,286.3
2022 Zhou	FMFP with 4 FMs	*Idem ditto*	EHR data	Yes	Single	No	No	39,286.3
2022 Zhou	FMFP with 5 FMs	*Idem ditto*	EHR data	Yes	Single	No	No	39,286.3
2022 Zhou	FMFP with 6 FMs	*Idem ditto*	EHR data	Yes	Single	No	No	39,286.3
2022 Zhou	FMFP with 7 FMs	*Idem ditto*	EHR data	Yes	Single	No	No	39,286.3
2022 Zhou	FMFP with 8 FMs	*Idem ditto*	EHR data	Yes	Single	No	No	39,286.3

2022 Zhou	FMFP with 9 FMs	*Idem ditto*	EHR data	Yes	Single	No	No	39,286.3
2022 Zhou	FMFP with 10 FMs	*Idem ditto*	EHR data	Yes	Single	No	No	39,286.3
2022 Zhou	wFM	*Idem ditto*	EHR data	Yes	Single	No	No	39,286.3
2022 Zhou	FM	*Idem ditto*	EHR data	Yes	Single	No	No	39,286.3
2022 Zhou	SVM-Poly	*Idem ditto*	EHR data	Yes	Single	No	No	39,286.3
2022 Zhou	SVM-Sigmoid	*Idem ditto*	EHR data	Yes	Single	No	No	39,286.3
2022 Zhou	SVM-RBF	*Idem ditto*	EHR data	Yes	Single	No	No	39,286.3
2022 Zhou	MLP	*Idem ditto*	EHR data	Yes	Single	No	No	39,286.3
2022 Zhou	XGBoost	*Idem ditto*	EHR data	Yes	Single	No	No	39,286.3
2022 Zhou	Lightgbm	*Idem ditto*	EHR data	Yes	Single	No	No	39,286.3
2022 Zhou	Catboost	*Idem ditto*	EHR data	Yes	Single	No	No	39,286.3
2022 Zhou	GBM	*Idem ditto*	EHR data	Yes	Single	No	No	39,286.3
2022 Zhou	RF	*Idem ditto*	EHR data	Yes	Single	No	No	39,286.3
2023 Karpinski	-	Hospitalization for any cause	Claims data	Yes	Single	No	No	10.5

EPV, events per variable; CCI, Charlson comorbidity index; NA, not applicable; CART, classification and regression trees; GBDT, gradient boosted decision trees; LR, logistic regression; RF, random forest; KNN, K-nearest neighbour; SVM, support vector machine; wFMFP, weighted factorisation machine with fuzzy partition; wFM, weighted factorisation machine; EHR, electronic health record; FMFP, factorisation machine with fuzzy partition; FM, factorisation machine; RBF, radial basis function; MLP, multilayer perceptron; GBM, gradient boosting machine.

[a] How was the outcome measured.

[b] Was the outcome measured consistently.

[c] Was the outcome a single outcome or a composite outcome.

[d] Was the outcome assessed with blinding for the predictors.

[e] Were predictors part of the outcome definition.

Table S3. Overview of general characteristics of the models in the identified studies that were not the primary focus of the study.

Study	Model	N	Events	Modelling method	Candidate predictors[a]	Predictor selection method	Penali-zation	Validation method	CITL	Slope[b]	C-statistic
2020 Wu	CART	22,859	5,754	CART	34	Model-inherent	None	5-fold cross-validation	*NR*	*NR*	0.73
2020 Wu	GBDT	22,859	5,754	GBDT	34	Model-inherent	None	5-fold cross-validation	*NR*	*NR*	0.76
2020 Wu	LR	22,859	5,754	LR	34	Best 10 predictors in RF	None	5-fold cross-validation	*NR*	*NR*	0.74
2020 Wu	RF	22,859	5,754	RF	34	Model-inherent	None	5-fold cross-validation	*NR*	*NR*	0.76
2020 Wu	Score	22,859	5,754	LR	34	Predictors with p <0.05 in LR model	None	Random split (80% development, 20% validation)	*NR*	*NR*	*NR*
2021 Kong	KNN	23,992	7,270	KNN	15+	Model-inherent	None	5-fold cross-validation	*NR*	*NR*	0.72 (0.70-0.74)
2021 Kong	LR	23,992	7,270	LR	15+	No selection	None	5-fold cross-validation	*NR*	*NR*	0.74 (0.73-0.75)
2021 Kong	RF	23,992	7,270	RF	15+	Model-inherent	None	5-fold cross-validation	*NR*	*NR*	0.76 (0.75-0.77)
2021 Kong	SVM	23,992	7,270	SVM	15+	Model-inherent	None	5-fold cross-validation	*NR*	*NR*	0.73 (0.72-0.74)
2022 Zhou	wFMFP with 3 wFMs	418,305	392,863	wFMFP with 3 wFMs	10+	No selection	None	None	*NR*	*NR*	*NR*
2022 Zhou	wFMFP with 4 wFMs	418,305	392,863	wFMFP with 4 wFMs	10+	No selection	None	None	*NR*	*NR*	*NR*
2022 Zhou	wFMFP with 6 wFMs	418,305	392,863	wFMFP with 6 wFMs	10+	No selection	None	None	*NR*	*NR*	*NR*
2022 Zhou	wFMFP with 7 wFMs	418,305	392,863	wFMFP with 7 wFMs	10+	No selection	None	None	*NR*	*NR*	*NR*
2022 Zhou	wFMFP with 8 wFMs	418,305	392,863	wFMFP with 8 wFMs	10+	No selection	None	None	*NR*	*NR*	*NR*
2022 Zhou	wFMFP with 9 wFMs	418,305	392,863	wFMFP with 9 wFMs	10+	No selection	None	None	*NR*	*NR*	*NR*
2022 Zhou	wFMFP with 10 wFMs	418,305	392,863	wFMFP with 10 wFMs	10+	No selection	None	None	*NR*	*NR*	*NR*
2022 Zhou	FMFP with 3 FMs	418,305	392,863	FMFP with 3 FMs	10+	No selection	None	None	*NR*	*NR*	*NR*
2022 Zhou	FMFP with 4 FMs	418,305	392,863	FMFP with 4 FMs	10+	No selection	None	None	*NR*	*NR*	*NR*
2022 Zhou	FMFP with 5 FMs	418,305	392,863	FMFP with 5 FMs	10+	No selection	None	None	*NR*	*NR*	*NR*
2022 Zhou	FMFP with 6 FMs	418,305	392,863	FMFP with 6 FMs	10+	No selection	None	None	*NR*	*NR*	*NR*
2022 Zhou	FMFP with 7 FMs	418,305	392,863	FMFP with 7 FMs	10+	No selection	None	None	*NR*	*NR*	*NR*
2022 Zhou	FMFP with 8 FMs	418,305	392,863	FMFP with 8 FMs	10+	No selection	None	None	*NR*	*NR*	*NR*
2022 Zhou	FMFP with 9 FMs	418,305	392,863	FMFP with 9 FMs	10+	No selection	None	None	*NR*	*NR*	*NR*
2022 Zhou	FMFP with 10 FMs	418,305	392,863	FMFP with 10 FMs	10+	No selection	None	None	*NR*	*NR*	*NR*
2022 Zhou	wFM	418,305	392,863	wFM	10+	No selection	None	None	*NR*	*NR*	*NR*
2022 Zhou	FM	418,305	392,863	FM	10+	No selection	None	None	*NR*	*NR*	*NR*
2022 Zhou	SVM-Poly	418,305	392,863	Polynomial kernel-based SVM	10+	No selection	None	None	*NR*	*NR*	*NR*
2022 Zhou	SVM-Sigmoid	418,305	392,863	Sigmoid kernel-based SVM	10+	No selection	None	None	*NR*	*NR*	*NR*
2022 Zhou	SVM-RBF	418,305	392,863	RBF kernel-based SVM	10+	No selection	None	None	*NR*	*NR*	*NR*
2022 Zhou	MLP	418,305	392,863	Multilayer perceptron	10+	No selection	None	None	*NR*	*NR*	*NR*
2022 Zhou	XGBoost	418,305	392,863	Extreme boosting	10+	No selection	None	None	*NR*	*NR*	*NR*
2022 Zhou	Lightgbm	418,305	392,863	GBDT	10+	No selection	None	None	*NR*	*NR*	*NR*
2022 Zhou	Catboost	418,305	392,863	GBDT	10+	No selection	None	None	*NR*	*NR*	*NR*
2022 Zhou	GBM	418,305	392,863	GBDT	10+	No selection	None	None	*NR*	*NR*	*NR*
2022 Zhou	RF	418,305	392,863	RF	10+	No selection	None	None	*NR*	*NR*	*NR*

CITL, calibration-in-the-large; NR, not reported; NA, not applicable; LR, logistic regression; CART, classification and regression trees; GBDT, gradient boosted decision trees; RF, random forest; KNN, K-nearest neighbour; SVM, support vector machine; wFMFP, weighted factorisation machine with fuzzy partition; wFM, weighted factorisation machine; FMFP, factorisation machine with fuzzy partition; FM, factorisation machine; RBF, radial basis function.

[a] A plus sign indicates that only the lower bound was known. For instance, 23+ means at least 23 candidate predictors, but possibly more.

[b] Calibration slope

Table S4. Additional information on all identified models.

Study	Model	Eligibility criteria	Patient recruitment method	Missing data	Missing data handling	Model -ling[a]	Candidate predictor selection	Other presen-tation	Data comparison[b]	Comparison with other studies[c]
2003 Fried	CCI	Inclusion criteria: *comorbidity at start of PD*<br>Exclusion criteria: *missing comorbidity data, failing PD training, history of HD >1 month or transplant*	Register	0.0%	Participants with missing comorbiditiy were excluded	*NR*	*NR*	No	*NA*	*NR*
2003 Fried	Davies	*Idem ditto*	Register	0.0%	Participants with missing comorbiditiy were excluded	*NR*	*NR*	No	*NA*	*NR*
2013 Perkins	-	Inclusion criteria: *age 18-88 years, CKD stage 3-5, no history of KRT, hospitalised in study medical centres, primary discharge for HF*<br>Exclusion criteria: *death during primary hospitalisation, death within 30 days of discharge, discharge to hospice or acute care facility, prior history of ESKD or solid-organ transplantation*	Register	*NR*	Multiple imputation	*NR*	*NR*	No	*NA*	Fills a gap in the literature (no other models on readmission after HF hospitalisation in CKD patients)
2016 Flythe	Admission	Inclusion criteria: *age >18 years, ESKD before index hospitalisation, inpatient HD during index admission*<br>Exclusion criteria: *PD, observation hospital stay, discharge from psychiatric or inpatient rehabilitation services, receipt of kidney transplant during hospitalisation, declaration of ESKD during hospitalisation, recovery of kidney function during hospitalisation, missing data in medication or some laboratory values*	*NR*	0.0%	Participants with missing medication data were excluded and missing laboratory data were not considered as predictors	*NR*	Literature and clinical knowledge	No	*NA*	*NR*
2016 Flythe	Discharge	*Idem ditto*	*NR*	0.0%	Exclusion of missing medication, missing laboratory not considered as predictors	*NR*	Literature and clinical knowledge	No	*NA*	*NR*
2020 Wong	1 hosp-italization	Inclusion criteria: *dialysis at study facilities at baseline and in the two weeks one year before baseline*	Register	0.0%	Residual kidney function imputed with 0 and not considered in modelling, individuals	*NR*	Association with clinical outcomes	No	*NA*	Compared to other malnutrition scores, not

Study	Model	Eligibility criteria	Patient recruitment method	Missing data	Missing data handling	Model -ling[a]	Candidate predictor selection	Other presen-tation	Data comparison[b]	Comparison with other studies[c]
		Exclusion criteria: *missing data except residual kidney function, <6 months follow-up*			with other missing data excluded					directly to hospitalisation prediction models.
2020 Wong	6 hospi-talizations	*Idem ditto*	Register	0.0%	*Idem ditto*	*NR*	Association with clinical outcomes	No	*NA*	*Idem ditto*
2020 Wu	CART	Inclusion criteria: *age 18-100 years and treated with PD* Exclusion criteria: *diagnosed with AKI or kidney transplantation, or died in the hospital, length of stay >30 days or readmission within a day after the previous hospital discharge*	Register	<15%	Analysed with 'missing' category	*NR*	Based on literature and clinical expertise	No	*NA*	Fills a gap in the literature (no other models on pLOS in PD)
2020 Wu	GBDT	*Idem ditto*	Register	<15%	*Idem ditto*	*NR*	*Idem ditto*	No	*NA*	*Idem ditto*
2020 Wu	LR	*Idem ditto*	Register	<15%	*Idem ditto*	*NR*	*Idem ditto*	No	*NA*	*Idem ditto*
2020 Wu	RF	*Idem ditto*	Register	<15%	*Idem ditto*	*NR*	*Idem ditto*	No	*NA*	*Idem ditto*
2020 Wu	Score	*Idem ditto*	Register	<15%	*Idem ditto*	*NR*	*Idem ditto*	Yes	No	*Idem ditto*
2021 Kong	KNN	Inclusion criteria: *age 18-100 years and treated with PD* Exclusion criteria: *diagnosed with AKI or kidney transplantation, or died in the hospital*	Register	<15%	Analysed with 'missing' category	*NR*	Based on literature and availability	No	*NA*	Fills a gap in the literature (no other models on pLOS in PD)
2021 Kong	LR	*Idem ditto*	Register	<15%	*Idem ditto*	*NR*	*Idem ditto*	No	*NA*	*Idem ditto*
2021 Kong	RF	*Idem ditto*	Register	<15%	*Idem ditto*	*NR*	*Idem ditto*	No	*NA*	*Idem ditto*
2021 Kong	SVM	*Idem ditto*	Register	<15%	*Idem ditto*	*NR*	*Idem ditto*	No	*NA*	*Idem ditto*
2021 Kong	Stacking	*Idem ditto*	Register	<15%	*Idem ditto*	*NR*	*Idem ditto*	No	*NA*	*Idem ditto*
2022 Zhou	wFMFP with 3 wFMs	Inclusion criteria: *65 years or older, CKD as primary hospital admission diagnosis*	EHR data	*NR*	*NR*	Multi-collin-earity	*NR*	No	*NA*	Unclear
2022 Zhou	wFMFP with 4 wFMs	*Idem ditto*	EHR data	*NR*	*NR*	*Idem ditto*	*NR*	No	*NA*	Unclear
2022 Zhou	wFMFP with 5 wFMs	*Idem ditto*	EHR data	*NR*	*NR*	*Idem ditto*	*NR*	No	*NA*	Unclear
2022 Zhou	wFMFP with 6 wFMs	*Idem ditto*	EHR data	*NR*	*NR*	*Idem ditto*	*NR*	No	*NA*	Unclear

Study	Model	Eligibility criteria	Patient recruitment method	Missing data	Missing data handling	Model -ling[a]	Candidate predictor selection	Other presen-tation	Data comparison[b]	Comparison with other studies[c]
2022 Zhou	wFMFP with 7 wFMs	*Idem ditto*	EHR data	*NR*	*NR*	*Idem ditto*	*NR*	No	*NA*	Unclear
2022 Zhou	wFMFP with 8 wFMs	*Idem ditto*	EHR data	*NR*	*NR*	*Idem ditto*	*NR*	No	*NA*	Unclear
2022 Zhou	wFMFP with 9 wFMs	*Idem ditto*	EHR data	*NR*	*NR*	*Idem ditto*	*NR*	No	*NA*	Unclear
2022 Zhou	wFMFP with 10 wFMs	*Idem ditto*	EHR data	*NR*	*NR*	*Idem ditto*	*NR*	No	*NA*	Unclear
2022 Zhou	FMFP with 3 FMs	*Idem ditto*	EHR data	*NR*	*NR*	*Idem ditto*	*NR*	No	*NA*	Unclear
2022 Zhou	FMFP with 4 FMs	*Idem ditto*	EHR data	*NR*	*NR*	*Idem ditto*	*NR*	No	*NA*	Unclear
2022 Zhou	FMFP with 5 FMs	*Idem ditto*	EHR data	*NR*	*NR*	*Idem ditto*	*NR*	No	*NA*	Unclear
2022 Zhou	FMFP with 6 FMs	*Idem ditto*	EHR data	*NR*	*NR*	*Idem ditto*	*NR*	No	*NA*	Unclear
2022 Zhou	FMFP with 7 FMs	*Idem ditto*	EHR data	*NR*	*NR*	*Idem ditto*	*NR*	No	*NA*	Unclear
2022 Zhou	FMFP with 8 FMs	*Idem ditto*	EHR data	*NR*	*NR*	*Idem ditto*	*NR*	No	*NA*	Unclear
2022 Zhou	FMFP with 9 FMs	*Idem ditto*	EHR data	*NR*	*NR*	*Idem ditto*	*NR*	No	*NA*	Unclear
2022 Zhou	FMFP with 10 FMs	*Idem ditto*	EHR data	*NR*	*NR*	*Idem ditto*	*NR*	No	*NA*	Unclear

Study	Model	Eligibility criteria	Patient recruitment method	Missing data	Missing data handling	Model -ling[a]	Candidate predictor selection	Other presen-tation	Data comparison[b]	Comparison with other studies[c]
2022 Zhou	wFM	*Idem ditto*	EHR data	*NR*	*NR*	*Idem ditto*	*NR*	No	*NA*	Unclear
2022 Zhou	FM	*Idem ditto*	EHR data	*NR*	*NR*	*Idem ditto*	*NR*	No	*NA*	Unclear
2022 Zhou	SVM-Poly	*Idem ditto*	EHR data	*NR*	*NR*	*Idem ditto*	*NR*	No	*NA*	Unclear
2022 Zhou	SVM-Sigmoid	*Idem ditto*	EHR data	*NR*	*NR*	*Idem ditto*	*NR*	No	*NA*	Unclear
2022 Zhou	SVM-RBF	*Idem ditto*	EHR data	*NR*	*NR*	*Idem ditto*	*NR*	No	*NA*	Unclear
2022 Zhou	MLP	*Idem ditto*	EHR data	*NR*	*NR*	*Idem ditto*	*NR*	No	*NA*	Unclear
2022 Zhou	XGBoost	*Idem ditto*	EHR data	*NR*	*NR*	*Idem ditto*	*NR*	No	*NA*	Unclear
2022 Zhou	Lightgbm	*Idem ditto*	EHR data	*NR*	*NR*	*Idem ditto*	*NR*	No	*NA*	Unclear
2022 Zhou	Catboost	*Idem ditto*	EHR data	*NR*	*NR*	*Idem ditto*	*NR*	No	*NA*	Unclear
2022 Zhou	GBM	*Idem ditto*	EHR data	*NR*	*NR*	*Idem ditto*	*NR*	No	*NA*	Unclear
2022 Zhou	RF	*Idem ditto*	EHR data	*NR*	*NR*	*Idem ditto*	*NR*	No	*NA*	Unclear
2023 Karpinski	-	Inclusion criteria: *enrolled in Medicare Part A and B on July 1 2018, diagnosis of CKD stage 3 to 5 between July 1 2017 to March 30 2018, no prior claims for dialysis, 18 years or older*	Claims data	0%	*NA*	*NR*	*NR*	No	Yes	Fills a gap in the literature (no other models for hospitalisation in CKD stages 3-5)

CCI, Charlson comorbidity index; NR, not reported; NA, not applicable; CKD, chronic kidney disease; ESKD, end-stage kidney disease; HF, heart failure; HD, haemodialysis; PD, peritoneal dialysis; CART, classification and regression trees; AKI, acute kidney injury; pLOS, prolonged length of stay; GBDT, gradient boosted decision trees; LR, logistic regression; RF, random forest; KNN, K-nearest neighbour; SVM, support vector machine; wFMFP, weighted factorisation machine with fuzzy partition; wFM, weighted factorisation machine; EHR, electronic health record; FMFP, factorisation machine with fuzzy partition; FM, factorisation machine; RBF, radial basis function; MLP, multilayer perceptron; GBM, gradient boosting machine.

[a] Were the modelling assumptions satisfied.

[b] Compared development and validation dataset.

[c] As reported in the study.

Table S5. Additional model performance measures reported for all identified models.

Study	Model	AIC	Brier score	ECI	Sensitivity	Specificity	PPV	NPV	GM	HL test	R^2	MSE	MAE	MAPE	MeAPE	Re-estimation due to performance
2003 Fried	CCI	850.2	-	-	-	-	-	-	-	-	-	-	-	-	-	No
2003 Fried	Davies	799.2	-	-	-	-	-	-	-	-	-	-	-	-	-	No
2013 Perkins	-	-	-	-	0.690	0.734	-	-	-	0.11	-	-	-	-	-	No
2016 Flythe	Admission	-	-	-	-	-	-	-	-	-	-	-	-	-	-	No
2016 Flythe	Discharge	-	-	-	-	-	-	-	-	-	-	-	-	-	-	No
2020 Wong	1 hospitalisation	-	-	-	0.41 (0.26-0.55)	0.88 (0.87-0.89)	-	-	-	-	-	-	-	-	-	No
2020 Wong	6 hospitalisations	-	-	-	0.52 (0.37-0.67)	0.88 (0.86-0.89)	-	-	-	-	-	-	-	-	-	No
2020 Wu	CART	-	0.163	8.173	-	-	-	-	-	-	-	-	-	-	-	No
2020 Wu	GBDT	-	0.158	7.891	-	-	-	-	-	-	-	-	-	-	-	No
2020 Wu	LR	-	0.161	8.036	-	-	-	-	-	-	-	-	-	-	-	No
2020 Wu	RF	-	0.158	7.883	-	-	-	-	-	-	-	-	-	-	-	No
2020 Wu	Score	-	-	-	-	-	-	-	-	-	-	-	-	-	-	No
2021 Kong	KNN	-	0.188	9.386	0.666	0.657	-	-	0.661	-	-	-	-	-	-	No
2021 Kong	LR	-	0.178	8.911	0.683	0.671	-	-	0.677	-	-	-	-	-	-	No
2021 Kong	RF	-	0.174	8.722	0.686	0.693	-	-	0.689	-	-	-	-	-	-	No
2021 Kong	SVM	-	0.187	9.342	0.656	0.690	-	-	0.673	-	-	-	-	-	-	No
2021 Kong	Stacking	-	0.174	8.691	0.680	0.701	-	-	0.690	-	-	-	-	-	-	No
2022 Zhou	wFMFP with 3 wFMs	-	-	-	-	-	-	-	-	-	0.8267	0.2764	0.2692	0.2458	0.2521	No
2022 Zhou	wFMFP with 4 wFMs	-	-	-	-	-	-	-	-	-	0.8518	0.2256	0.2263	0.2390	0.2338	No
2022 Zhou	wFMFP with 5 wFMs	-	-	-	-	-	-	-	-	-	0.8865	0.2181	0.2411	0.2311	0.2130	No
2022 Zhou	wFMFP with 6 wFMs	-	-	-	-	-	-	-	-	-	0.8762	0.2512	0.2142	0.2265	0.2286	No
2022 Zhou	wFMFP with 7 wFMs	-	-	-	-	-	-	-	-	-	0.8336	0.2292	0.2295	0.2389	0.2421	No
2022 Zhou	wFMFP with 8 wFMs	-	-	-	-	-	-	-	-	-	0.8349	0.2277	0.2221	0.2459	0.2333	No
2022 Zhou	wFMFP with 9 wFMs	-	-	-	-	-	-	-	-	-	0.8101	0.2305	0.2322	0.2474	0.2605	No
2022 Zhou	wFMFP with 10 wFMs	-	-	-	-	-	-	-	-	-	0.7758	0.2471	0.2302	0.2528	0.2747	No
2022 Zhou	FMFP with 3 FMs	-	-	-	-	-	-	-	-	-	0.7947	0.2633	0.2593	0.2836	0.2994	No
2022 Zhou	FMFP with 4 FMs	-	-	-	-	-	-	-	-	-	0.8019	0.2773	0.2667	0.2787	0.2856	No
2022 Zhou	FMFP with 5 FMs	-	-	-	-	-	-	-	-	-	0.8249	0.2628	0.2584	0.2924	0.3172	No
2022 Zhou	FMFP with 6 FMs	-	-	-	-	-	-	-	-	-	0.8383	0.2649	0.2854	0.2833	0.3174	No
2022 Zhou	FMFP with 7 FMs	-	-	-	-	-	-	-	-	-	0.8052	0.2734	0.2621	0.2881	0.3130	No
2022 Zhou	FMFP with 8 FMs	-	-	-	-	-	-	-	-	-	0.8495	0.2624	0.2973	0.2856	0.3127	No
2022 Zhou	FMFP with 9 FMs	-	-	-	-	-	-	-	-	-	0.7942	0.2735	0.3133	0.3000	0.3245	No
2022 Zhou	FMFP with 10 FMs	-	-	-	-	-	-	-	-	-	0.7538	0.2863	0.2973	0.3158	0.3182	No
2022 Zhou	wFM	-	-	-	-	-	-	-	-	-	0.8318	0.2368	0.2656	0.2916	0.3276	No

Study	Model	AIC	Brier score	ECI	Sensitivity	Specificity	PPV	NPV	GM	HL test	R^2	MSE	MAE	MAPE	MeAPE	Re-estimation due to performance
2022 Zhou	FM	-	-	-	-	-	-	-	-	-	0.7966	0.2490	0.2931	0.2984	0.3105	No
2022 Zhou	SVM-Poly	-	-	-	-	-	-	-	-	-	0.7718	0.2948	0.3172	0.2934	0.3123	No
2022 Zhou	SVM-Sigmoid	-	-	-	-	-	-	-	-	-	0.7348	0.2817	0.3291	0.2942	0.3027	No
2022 Zhou	SVM-RBF	-	-	-	-	-	-	-	-	-	0.7943	0.2771	0.3012	0.2987	0.3120	No
2022 Zhou	MLP	-	-	-	-	-	-	-	-	-	0.7943	0.2771	0.3012	0.2987	0.3220	No
2022 Zhou	XGBoost	-	-	-	-	-	-	-	-	-	0.8215	0.2406	0.2573	0.2761	0.3012	No
2022 Zhou	Lightgbm	-	-	-	-	-	-	-	-	-	0.8105	0.2309	0.2581	0.2789	0.3114	No
2022 Zhou	Catboost	-	-	-	-	-	-	-	-	-	0.8094	0.2314	0.2678	0.2749	0.3152	No
2022 Zhou	GBM	-	-	-	-	-	-	-	-	-	0.7109	0.2546	0.2832	0.2931	0.3125	No
2022 Zhou	RF	-	-	-	-	-	-	-	-	-	0.7724	0.3008	0.3165	0.3101	0.3159	No
2023 Karpinski	-	-	-	-	0.613	0.735	0.212	0.942	-	-	-	-	-	-	-	No

AIC, Akaike information criterion; ECI, estimated calibration index; PPV, positive predictive value; NPV, negative predictive value; GM, geometric mean; HL, Hosmer-Lemeshow; MSE, mean squared error; MAE, mean absolute error; MAPE, median absolute percentage error; MeAPE, median absolute percentage error; CCI, Charlson comorbidity index; CART, classification and regression trees; GBDT, gradient boosted decision trees; LR, logistic regression; RF, random forests; KNN, K-nearest neighbour; SVM, support vector machine; wFMFP, weighted factorisation machine with fuzzy partition; wFM, weighted factorisation machine; FMFP, factorisation machine with fuzzy partition; FM, factorisation machine; RBF, radial basis function; MLP, multilayer perceptron; GBM, gradient boosting machine.

Table S6. Subjective interpretation of all identified models.

Study	Model	Generalizability	Strengths	Limitations	Interpretation
2003 Fried	CCI	Only incident PD patients	-	- No model performance shown<br>- Inadequate handling of missing data<br>- Data driven predictor selection methods<br>- Categorization of continuous measures	The limitations of the model invalidate its use in practice.
2003 Fried	Davies	Only incident PD patients	-	*Idem ditto*	*Idem ditto*
2013 Perkins	-	CKD patients without KRT discharged after HF hospitalisation	- Missing data was imputed with multiple imputation<br>- Continuous variables were modelled according to association with outcome	- Small sample size<br>- Individuals excluded based on future information	The limitations of the model invalidate its use in practice.
2016 Flythe	Admission	Only HD patients	- Candidate predictors selected based on literature and clinical knowledge	- Inadequate handling of missing data<br>- Data driven predictor selection methods<br>- Small sample size<br>- Individuals excluded based on future information	The limitations of the model invalidate its use in practice.
2016 Flythe	Discharge	Only HD patients	*Idem ditto*	- Inadequate handling of missing data<br>- Data driven predictor selection methods<br>- Small sample size	*Idem ditto*
2020 Wong	1 hospi-talization	HD patients with dialysis vintage >1 year	- Large sample size<br>- Compared multiple modelling strategies to choose best model<br>- Took into account non-linearity of continuous variables<br>- Intermediate validation with randomly selected validation cohort	- Inadequate handling of missing data<br>- Individuals excluded based on future information	The limitations of the model invalidate its use in practice.
2020 Wong	6 hospi-talizations	HD patients with dialysis vintage >1 year	- Compared multiple modelling strategies to choose best model<br>- Took into account non-linearity of continuous variables<br>- Intermediate validation with randomly selected validation cohort	- Small sample size<br>- Inadequate handling of missing data<br>- Individuals excluded based on future information	*Idem ditto*
2020 Wu	CART	PD patients admitted to hospital living in China	- Candidate predictors selected based on literature and clinical knowledge<br>- Large sample size	- Inadequate handling of missing data<br>- Individuals excluded based on future information	The limitations of the model invalidate its use in practice. Due to predictors specific to China, this model cannot be used outside of China.
2020 Wu	GBDT	*Idem ditto*	*Idem ditto*	*Idem ditto*	*Idem ditto*
2020 Wu	LR	*Idem ditto*	*Idem ditto*	*Idem ditto*	*Idem ditto*
2020 Wu	RF	*Idem ditto*	*Idem ditto*	*Idem ditto*	*Idem ditto*
2020 Wu	Score	*Idem ditto*	- Candidate predictors selected based on literature and clinical knowledge<br>- Large sample size<br>- Combined multiple modelling techniques to derive final model	*Idem ditto*	*Idem ditto*
2021 Kong	KNN	PD patients admitted to	- Candidate predictors selected based on literature and clinical knowledge<br>- Large sample size	- Inadequate handling of missing data<br>- Individuals excluded based on future information	The limitations of the model invalidate its use in practice. Due to predictors specific to

Study	Model	Generalizability	Strengths	Limitations	Interpretation
		hospital living in China			China, this model cannot be used outside of China.
2021 Kong	LR	*Idem ditto*	*Idem ditto*	*Idem ditto*	*Idem ditto*
2021 Kong	RF	*Idem ditto*	*Idem ditto*	*Idem ditto*	*Idem ditto*
2021 Kong	SVM	*Idem ditto*	*Idem ditto*	*Idem ditto*	*Idem ditto*
2021 Kong	Stacking	*Idem ditto*	- Candidate predictors selected based on literature and clinical knowledge<br>- Large sample size<br>- Combined multiple modelling techniques to derive final model	*Idem ditto*	*Idem ditto*
2022 Zhou	wFMFP with 3 wFMs	Patients admitted to the hospital for CKD	- Large sample size<br>- Used multiple modelling strategies	- No useful model performance shown<br>- Unclear handling of missing data<br>- Unclear what predictors were included	The study is not clear enough to determine whether the model is of use.
2022 Zhou	wFMFP with 4 wFMs	*Idem ditto*	*Idem ditto*	*Idem ditto*	*Idem ditto*
2022 Zhou	wFMFP with 5 wFMs	*Idem ditto*	*Idem ditto*	*Idem ditto*	*Idem ditto*
2022 Zhou	wFMFP with 6 wFMs	*Idem ditto*	*Idem ditto*	*Idem ditto*	*Idem ditto*
2022 Zhou	wFMFP with 7 wFMs	*Idem ditto*	*Idem ditto*	*Idem ditto*	*Idem ditto*
2022 Zhou	wFMFP with 8 wFMs	*Idem ditto*	*Idem ditto*	*Idem ditto*	*Idem ditto*
2022 Zhou	wFMFP with 9 wFMs	*Idem ditto*	*Idem ditto*	*Idem ditto*	*Idem ditto*
2022 Zhou	wFMFP with 10 wFMs	*Idem ditto*	*Idem ditto*	*Idem ditto*	*Idem ditto*
2022 Zhou	FMFP with 3 FMs	*Idem ditto*	*Idem ditto*	*Idem ditto*	*Idem ditto*
2022 Zhou	FMFP with 4 FMs	*Idem ditto*	*Idem ditto*	*Idem ditto*	*Idem ditto*
2022 Zhou	FMFP with 5 FMs	*Idem ditto*	*Idem ditto*	*Idem ditto*	*Idem ditto*

Study	Model	Generalizability	Strengths	Limitations	Interpretation
2022 Zhou	FMFP with 6 FMs	*Idem ditto*	*Idem ditto*	*Idem ditto*	*Idem ditto*
2022 Zhou	FMFP with 7 FMs	*Idem ditto*	*Idem ditto*	*Idem ditto*	*Idem ditto*
2022 Zhou	FMFP with 8 FMs	*Idem ditto*	*Idem ditto*	*Idem ditto*	*Idem ditto*
2022 Zhou	FMFP with 9 FMs	*Idem ditto*	*Idem ditto*	*Idem ditto*	*Idem ditto*
2022 Zhou	FMFP with 10 FMs	*Idem ditto*	*Idem ditto*	*Idem ditto*	*Idem ditto*
2022 Zhou	wFM	*Idem ditto*	*Idem ditto*	*Idem ditto*	*Idem ditto*
2022 Zhou	FM	*Idem ditto*	*Idem ditto*	*Idem ditto*	*Idem ditto*
2022 Zhou	SVM-Poly	*Idem ditto*	*Idem ditto*	*Idem ditto*	*Idem ditto*
2022 Zhou	SVM-Sigmoid	*Idem ditto*	*Idem ditto*	*Idem ditto*	*Idem ditto*
2022 Zhou	SVM-RBF	*Idem ditto*	*Idem ditto*	*Idem ditto*	*Idem ditto*
2022 Zhou	MLP	*Idem ditto*	*Idem ditto*	*Idem ditto*	*Idem ditto*
2022 Zhou	XGBoost	*Idem ditto*	*Idem ditto*	*Idem ditto*	*Idem ditto*
2022 Zhou	Lightgbm	*Idem ditto*	*Idem ditto*	*Idem ditto*	*Idem ditto*
2022 Zhou	Catboost	*Idem ditto*	*Idem ditto*	*Idem ditto*	*Idem ditto*
2022 Zhou	GBM	*Idem ditto*	*Idem ditto*	*Idem ditto*	*Idem ditto*
2022 Zhou	RF	*Idem ditto*	*Idem ditto*	*Idem ditto*	*Idem ditto*
2023 Karpinski	-	CKD patients stage 3-5 not on dialysis	- Large sample size<br>- Chose the best model out of multiple strategies	- Unclear moment of use (random calendar date)<br>- Model performance unclear<br>- Data driven predictor selection methods	The model performance cannot be adequately assessed to determine its use in practice. The model is presented for use.

CCI, Charlson comorbidity index; PD, peritoneal dialysis patients; CKD, chronic kidney disease; KRT, kidney replacement therapy; HF, heart failure; HD, haemodialysis patients; CART, classification and regression trees; GBDT, gradient boosted decision trees; LR, logistic regression; RF, random forests; KNN, K-nearest neighbour; SVM, support vector machine; wFMFP, weighted factorisation machine with fuzzy partition; wFM, weighted factorisation machine; FMFP, factorisation machine with fuzzy partition; FM, factorisation machine; RBF, radial basis function; MLP, multilayer perceptron; GBM, gradient boosting machine.

Table S7. Risk of bias and applicability according to the PROBAST (complete).

Study	Model	Participants: Appropriate data	Participants: Appropriate eligibility	Participants: Overall	Participants: Applicability	Predictors: Consistent definition/assessment	Predictors: Blinded assessment	Predictors: Available at intended model use	Predictors: Overall	Predictors: Applicability	Outcome: Appropriate definition	Outcome: Prespecified/standard definition	Outcome: No predictors in definition	Outcome: Consistent definition/assessment	Outcome: Blinded assessment	Outcome: Appropriate time interval	Outcome: Overall	Outcome: Applicability	Analysis: Reasonable number of events	Analysis: Handling of predictors	Analysis: All enrolled participants used	Analysis: Missing data handling	Analysis: No univariable predictor selection	Analysis: Handling of complexities	Analysis: Performance assessment	Analysis: Accounting for overfitting	Analysis: Corresponding predictor weights	Analysis: Overall	Analysis: Applicability	Overall: Risk of bias	Overall: Applicability
2003 Fried	CCI	Y	N	L	L	PY	PY	Y	L	L	Y	Y	Y	Y	PY	Y	L	L	PY	N	Y	N	Y	N	N	N	NR	H	L	H	L
	Davies	Y	N	L	L	PY	PY	Y	L	L	Y	Y	Y	Y	PY	Y	L	L	PY	N	Y	N	Y	N	N	N	NR	H	L	H	L
2013 Perkins	-	Y	N	H	L	PY	PY	Y	L	L	Y	Y	Y	Y	PY	Y	L	L	N	PY	Y	Y	N	N	N	N	NR	H	L	H	L
2016 Flythe	Admission	Y	N	H	L	PY	PY	Y	L	L	Y	Y	Y	Y	PY	Y	L	L	N	PN	Y	N	N	N	N	N	NR	H	L	H	L
	Discharge	Y	Y	L	L	PY	PY	Y	L	L	Y	Y	Y	Y	PY	Y	L	L	N	PN	Y	N	N	N	N	N	NR	H	L	H	L
2020 Wong	1 hospitalization	Y	N	H	L	PY	PY	Y	L	L	Y	PY	Y	Y	PY	Y	L	L	Y	Y	Y	N	Y	N	Y	Y	NR	H	L	H	L
	6 hospitalizations	Y	N	H	L	PY	PY	Y	L	L	Y	N	Y	Y	PY	Y	L	L	N	Y	Y	N	Y	N	Y	N	NR	H	L	H	L
2020 Wu	CART	Y	N	H	L	PY	PY	Y	L	L	Y	PY	Y	Y	PY	Y	L	L	Y	PN	Y	N	Y	N	N	Y	NR	H	L	H	L
	GBDT	Y	N	H	L	PY	PY	Y	L	L	Y	PY	Y	Y	PY	Y	L	L	Y	PN	Y	N	Y	N	N	Y	NR	H	L	H	L
	LR	Y	N	H	L	PY	PY	Y	L	L	Y	PY	Y	Y	PY	Y	L	L	Y	PN	Y	N	Y	N	N	Y	NR	H	L	H	L
	RF	Y	N	H	L	PY	PY	Y	L	L	Y	PY	Y	Y	PY	Y	L	L	Y	PN	Y	N	Y	N	N	Y	NR	H	L	H	L
	Stacking	Y	N	H	L	PY	PY	Y	L	L	Y	PY	Y	Y	PY	Y	L	L	Y	PN	Y	N	Y	N	N	Y	NR	H	L	H	L
2021 Kong	KNN	Y	N	H	L	PY	PY	Y	L	L	Y	PY	Y	Y	PY	Y	L	L	Y	PN	Y	N	Y	N	Y	Y	NR	H	L	H	L
	LR	Y	N	H	L	PY	PY	Y	L	L	Y	PY	Y	Y	PY	Y	L	L	Y	PN	Y	N	Y	N	Y	Y	NR	H	L	H	L
	RF	Y	N	H	L	PY	PY	Y	L	L	Y	PY	Y	Y	PY	Y	L	L	Y	PN	Y	N	Y	N	Y	Y	NR	H	L	H	L
	SVM	Y	N	H	L	PY	PY	Y	L	L	Y	PY	Y	Y	PY	Y	L	L	Y	PN	Y	N	Y	N	Y	Y	NR	H	L	H	L
	Stacking	Y	N	H	L	PY	PY	Y	L	L	Y	PY	Y	Y	PY	Y	L	L	Y	PN	Y	N	Y	N	Y	Y	NR	H	L	H	L
2022 Zhou	wFMFP with 3 wFMs	Y	Y	L	L	PY	PY	Y	L	L	Y	Y	Y	Y	PY	Y	L	L	Y	PY	Y	NR	Y	N	N	Y	NR	U	L	U	L
	wFMFP with 4 wFMs	Y	Y	L	L	PY	PY	Y	L	L	Y	Y	Y	Y	PY	Y	L	L	Y	PY	Y	NR	Y	N	N	Y	NR	U	L	U	L
	wFMFP with 5 wFMs	Y	Y	L	L	PY	PY	Y	L	L	Y	Y	Y	Y	PY	Y	L	L	Y	PY	Y	NR	Y	N	N	Y	NR	U	L	U	L
	wFMFP with 6 wFMs	Y	Y	L	L	PY	PY	Y	L	L	Y	Y	Y	Y	PY	Y	L	L	Y	PY	Y	NR	Y	N	N	Y	NR	U	L	U	L
	wFMFP with 7 wFMs	Y	Y	L	L	PY	PY	Y	L	L	Y	Y	Y	Y	PY	Y	L	L	Y	PY	Y	NR	Y	N	N	Y	NR	U	L	U	L
	wFMFP with 8 wFMs	Y	Y	L	L	PY	PY	Y	L	L	Y	Y	Y	Y	PY	Y	L	L	Y	PY	Y	NR	Y	N	N	Y	NR	U	L	U	L
	wFMFP with 9 wFMs	Y	Y	L	L	PY	PY	Y	L	L	Y	Y	Y	Y	PY	Y	L	L	Y	PY	Y	NR	Y	N	N	Y	NR	U	L	U	L
	wFMFP with 10 wFMs	Y	Y	L	L	PY	PY	Y	L	L	Y	Y	Y	Y	PY	Y	L	L	Y	PY	Y	NR	Y	N	N	Y	NR	U	L	U	L
	FMFP with 3 FMs	Y	Y	L	L	PY	PY	Y	L	L	Y	Y	Y	Y	PY	Y	L	L	Y	PY	Y	NR	Y	N	N	Y	NR	U	L	U	L

		Participants				Predictors					Outcome								Analysis											Overall	
Study	Model	Appropriate data	Appropriate eligibility	Overall	Applicability	Consistent definition/assessment	Blinded assessment	Available at intended model use	Overall	Applicability	Appropriate definition	Prespecified/standard definition	No predictors in definition	Consistent definition/assessment	Blinded assessment	Appropriate time interval	Overall	Applicability	Reasonable number of events	Handling of predictors	All enrolled participants used	Missing data handling	No univariable predictor selection	Handling of complexities	Performance assessment	Accounting for overfitting	Corresponding predictor weights	Overall	Applicability	Risk of bias	Applicability
	FMFP with 4 FMs	Y	Y	L	L	PY	PY	Y	L	L	Y	Y	Y	Y	PY	Y	L	L	Y	PY	Y	NR	Y	N	N	Y	NR	U	L	U	L
	FMFP with 5 FMs	Y	Y	L	L	PY	PY	Y	L	L	Y	Y	Y	Y	PY	Y	L	L	Y	PY	Y	NR	Y	N	N	Y	NR	U	L	U	L
	FMFP with 6 FMs	Y	Y	L	L	PY	PY	Y	L	L	Y	Y	Y	Y	PY	Y	L	L	Y	PY	Y	NR	Y	N	N	Y	NR	U	L	U	L
	FMFP with 7 FMs	Y	Y	L	L	PY	PY	Y	L	L	Y	Y	Y	Y	PY	Y	L	L	Y	PY	Y	NR	Y	N	N	Y	NR	U	L	U	L
	FMFP with 8 FMs	Y	Y	L	L	PY	PY	Y	L	L	Y	Y	Y	Y	PY	Y	L	L	Y	PY	Y	NR	Y	N	N	Y	NR	U	L	U	L
	FMFP with 9 FMs	Y	Y	L	L	PY	PY	Y	L	L	Y	Y	Y	Y	PY	Y	L	L	Y	PY	Y	NR	Y	N	N	Y	NR	U	L	U	L
	FMFP with 10 FMs	Y	Y	L	L	PY	PY	Y	L	L	Y	Y	Y	Y	PY	Y	L	L	Y	PY	Y	NR	Y	N	N	Y	NR	U	L	U	L
	wFM	Y	Y	L	L	PY	PY	Y	L	L	Y	Y	Y	Y	PY	Y	L	L	Y	PY	Y	NR	Y	N	N	Y	NR	U	L	U	L
	FM	Y	Y	L	L	PY	PY	Y	L	L	Y	Y	Y	Y	PY	Y	L	L	Y	PY	Y	NR	Y	N	N	Y	NR	U	L	U	L
	SVM-Poly	Y	Y	L	L	PY	PY	Y	L	L	Y	Y	Y	Y	PY	Y	L	L	Y	PY	Y	NR	Y	N	N	Y	NR	U	L	U	L
	SVM-Sigmoid	Y	Y	L	L	PY	PY	Y	L	L	Y	Y	Y	Y	PY	Y	L	L	Y	PY	Y	NR	Y	N	N	Y	NR	U	L	U	L
	SVM-RBF	Y	Y	L	L	PY	PY	Y	L	L	Y	Y	Y	Y	PY	Y	L	L	Y	PY	Y	NR	Y	N	N	Y	NR	U	L	U	L
	MLP	Y	Y	L	L	PY	PY	Y	L	L	Y	Y	Y	Y	PY	Y	L	L	Y	PY	Y	NR	Y	N	N	Y	NR	U	L	U	L
	XGBoost	Y	Y	L	L	PY	PY	Y	L	L	Y	Y	Y	Y	PY	Y	L	L	Y	PY	Y	NR	Y	N	N	Y	NR	U	L	U	L
	Lightgbm	Y	Y	L	L	PY	PY	Y	L	L	Y	Y	Y	Y	PY	Y	L	L	Y	PY	Y	NR	Y	N	N	Y	NR	U	L	U	L
	Catboost	Y	Y	L	L	PY	PY	Y	L	L	Y	Y	Y	Y	PY	Y	L	L	Y	PY	Y	NR	Y	N	N	Y	NR	U	L	U	L
	GBM	Y	Y	L	L	PY	PY	Y	L	L	Y	Y	Y	Y	PY	Y	L	L	Y	PY	Y	NR	Y	N	N	Y	NR	U	L	U	L
	RF	Y	Y	L	L	PY	PY	Y	L	L	Y	Y	Y	Y	PY	Y	L	L	Y	PY	Y	NR	Y	N	N	Y	NR	U	L	U	L
2023 Karpinski	-	Y	Y	L	L	PY	PY	Y	L	L	Y	Y	PY	Y	PY	Y	L	L	Y	Y	Y	Y	Y	N	N	Y	NR	L	L	L	L

CCI, Charlson comorbidity index; Y, yes; N, no; L, low; PY, probably yes; NR, not reported; H, high; PN, probably no; CART, classification and regression trees; GBDT, gradient boosted decision trees; LR, logistic regression; RF, random forest; KNN, K-nearest neighbour; SVM, support vector machine; wFMFP, weighted factorisation machine with fuzzy partition; wFM, weighted factorisation machine; FMFP, factorisation machine with fuzzy partition; FM, factorisation machine; RBF, radial basis function; MLP, multilayer perceptron; GBM, gradient boosting machine.

Table S8. Overview of predictors included in the identified prediction models.

Study	Model	Predictor	Type	Definition	Measurement	Blinded assess-ment	Handling in model
2003 Fried	CCI	CCI	Comorbidity	*NR*	Calculated from comorbidities in register	*NA*	Categorized
		Albumin	Laboratory value	*NR*	Register	*NA*	*NR*
		Race	Demographic	*NR*	Register	*NA*	Binary
2003 Fried	Davies	Davies score	Comorbidity	*NR*	Calculated from comorbidities in hospital records	*NA*	Categorized
		Albumin	Laboratory value	*NR*	Register	*NA*	*NR*
		Race	Demographic	*NR*	Register	*NA*	Binary
		Age	Demographic	*NR*	Register	*NA*	*NR*
2013 Perkins		Admitted through emergency department	Hospitalisation characteristic	*NA*	Register	*NA*	Binary
		Admitted in transfer from other hospital	Hospitalisation characteristic	*NA*	Register	*NA*	Binary
		Number of clinic visits (primary care or other) in 3 months prior to index admission	Healthcare access	*NA*	Register	*NA*	Linear
		Discharged to facility other than home	Hospitalisation characteristic	*NA*	Register	*NA*	Binary
		Length of stay (index hospitalisation)	Hospitalisation characteristic	*NA*	Register	*NA*	Linear
		Fluid and electrolyte disorder	Comorbidity	*NR*	Register	*NA*	Binary
		Other nervous system disorder	Comorbidity	*NR*	Register	*NA*	Binary
		Other circulatory disease	Comorbidity	*NR*	Register	*NA*	Binary
		COPD and/or bronchiectasis	Comorbidity	*NR*	Register	*NA*	Binary
		Asthma	Comorbidity	*NR*	Register	*NA*	Binary
		Abdominal hernia	Comorbidity	*NR*	Register	*NA*	Binary
		Gastrointestinal haemorrhage	Comorbidity	*NR*	Register	*NA*	Binary
		Other disease of kidney/ureter	Comorbidity	*NR*	Register	*NA*	Binary
		Calcitriol at admission	Medication	*NR*	Register	*NA*	Binary
		Narcotic at admission	Medication	*NR*	Register	*NA*	Binary
		Last outpatient systolic blood pressure prior to hospital admission	Vital	*NR*	Register	*NA*	Linear
		Last outpatient diastolic blood pressure prior to admission	Vital	*NR*	Register	*NA*	Linear
		Last outpatient diastolic blood pressure	Vital	*NR*	Register	*NA*	Quadratic
		Admission white blood cell count	Laboratory value	*NR*	Register	*NA*	Linear
		Admission haemoglobin	Laboratory value	*NR*	Register	*NA*	Linear
		Admission serum sodium	Laboratory value	*NR*	Register	*NA*	Linear
		Negative outpatient proteinuria testing (vs. test not ordered)	Laboratory value	*NR*	Register	*NA*	Binary
		Positive outpatient proteinuria testing (vs. test not ordered)	Laboratory value	*NR*	Register	*NA*	Binary
2016 Flythe	Admission	Malignancy	Comorbidity	*NR*	Register	*NA*	Binary
		Hospitalisations in the prior year >= 3	Healthcare access	*NR*	Register	*NA*	Binary
		Outpatient use of warfarin	Medication use	*NR*	Register	*NA*	Binary
		Surgery admitting service	Healthcare access	*NA*	Register	*NA*	Binary
		Access	Dialysis characterstic	*NA*	Register	*NA*	Categorized
		Non-UNC-affiliated outpatient dialysis unit	Dialysis characteristic	*NA*	Register	*NA*	Binary
		Admission systolic blood pressure	Vital	*NR*	Register	*NA*	*NR*
	Discharge	Malignancy	Comorbidity	*NR*	Register	*NA*	Binary

Study	Model	Predictor	Type	Definition	Measurement	Blinded assess-ment	Handling in model
2016 Flythe		Hospitalisations in the prior year >= 3	Healthcare access	*NR*	Register	*NA*	Binary
		Outpatient medications >= 10	Medication use	*NR*	Register	*NA*	Binary
		Surgery admitting service	Healthcare access	*NA*	Register	*NA*	Binary
		Access	Dialysis characterstic	*NA*	Register	*NA*	Categorized
		Non-UNC-affiliated outpatient dialysis unit	Dialysis characteristic	*NA*	Register	*NA*	Binary
		Nadir intradialytic systolic BP <90 mmHg	Laboratory value	*NR*	Register	*NA*	*NR*
		Weekend discharge day	Index event characteristic	*NA*	Register	*NA*	Binary
		Albumin <3.3 g/dL	Laboratory value	*NR*	Register	*NA*	Binary
		Medication no. change	Medication use	*NR*	Register	*NA*	Categorized
2020 Wong	1 hospita-lization	Neutrophil-to-lymphocyte ratio	Laboratory value	*NR*	Register	*NA*	Smoothing splines (non-linear)
		Albumin	Laboratory value	*NR*	Register	*NA*	Smoothing splines (linear)
		Congestive heart failure	Comorbidity	*NR*	Register	*NA*	Binary
		Serum bicarbonate slope	Laboratory value	*NR*	Register	*NA*	Smoothing splines (non-linear)
		Serum creatinine	Laboratory value	*NR*	Register	*NA*	Smoothing splines (linear)
		Serum phosphate slope	Laboratory value	*NR*	Register	*NA*	Smoothing splines (non-linear)
		Dialysis vintage	Dialysis characteristic	*NR*	Register	*NA*	Smoothing splines (non-linear)
		Diabetes	Comorbidity	*NR*	Register	*NA*	Binary
		Serum phosphate	Laboratory value	*NR*	Register	*NA*	Smoothing splines (linear)
		Volume of urea distribution	Laboratory value	*NR*	Register	*NA*	Smoothing splines (non-linear)
		Age	Demographic	*NR*	Register	*NA*	Smoothing splines (linear)
		enPCR	Laboratory value	*NR*	Register	*NA*	Smoothing splines (linear)
2020 Wong	6 hospita-lizations	Neutrophil-to-lymphocyte ratio	Laboratory value	*NR*	Register	*NA*	Smoothing splines (non-linear)
		Bicarbonate slope	Laboratory value	*NR*	Register	*NA*	Smoothing splines (non-linear)
		Bicarbonate	Laboratory value	*NR*	Register	*NA*	Smoothing splines (linear)
		Diabetes	Comorbidity	*NR*	Register	*NA*	Binary
		Gastrointestinal bleeding	Comorbidity	*NR*	Register	*NA*	Binary
		Congestive heart failure	Comorbidity	*NR*	Register	*NA*	Binary
		Sex	Demographic	*NA*	Register	*NA*	Binary
		post-HD weight	Vital	*NR*	Register	*NA*	Smoothing splines (non-linear)
2020 Wu	CART, GBDT, LR, RF, and score	Admission reason	Hospitalisation characteristic	*NA*	Register	*NA*	Categorized
		Admission to the same hospital as the last or not	Hospitalisation characteristic	*NA*	Register	*NA*	Binary
		Number of hospitalisations within 6 months	Hospitalisation characteristic	*NA*	Register	*NA*	Dichotomized

Study	Model	Predictor	Type	Definition	Measurement	Blinded assess-ment	Handling in model
		Pulmonary infections	Comorbidity	*NR*	Register	*NA*	Binary
		Admission department	Hospitalisation characteristic	*NA*	Register	*NA*	Binary
		Place of residence	Demographic	*NR*	Register	*NA*	Categorized
2021 Kong	KNN, LR, RF, SVM, and stacking	Age	Demographic	*NR*	Register	*NA*	*NR*
		Sex	Demographic	*NA*	Register	*NA*	Binary
		Nationality	Demographic	*NR*	Register	*NA*	Categorized
		Reason for admission	Hospitalisation characteristic	*NA*	Register	*NA*	Categorized
		CKD cause	Medical history	*NR*	Register	*NA*	Categorized
		Comorbidities	Medical history	*NR*	Register	*NA*	*NR*
		Admission type	Hospitalisation characteristic	*NR*	Register	*NA*	Categorized
		Number of hospitalisations within 6 months	Healthcare access	Class 3 hospital admission	Register	*NA*	*NR*
		Number of emergency admissions within 6 months	Healthcare access	Class 3 hospital admission	Register	*NA*	*NR*
		Admission department	Hospitalisation characteristic	*NA*	Register	*NA*	Categorized
		Planned admission or not	Hospitalisation characteristic	*NR*	Register	*NA*	Binary
		Admission day of the week	Hospitalisation category	*NA*	Register	*NA*	Categorized
		Admitted in the same hospital as last admission	Hospitalisation characteristic	*NA*	Register	*NA*	Binary
		Place of residence	Demographic	*NR*	Register	*NA*	Categorized
		Insurance type	Demographic	*NA*	Register	*NA*	Categorized
2022 Zhou	wFMFPs, FMFPs, wFM, FM, SVM-Poly, SVM-Sigmoid, SVM-RFB MLP, XGBoost, Lightgbm, Catboost, GBM, RF	Age	Demographic	*NA*	EHR data	*NA*	Not reported
		District of residence	Demographic	Kwun Tong, Southern, Kwai Tsing, Eastern, etc. (total 18)	EHR data	*NA*	Categorical
		Admission specialty	Hospitalisation characteristic	Medicine or clinical oncology	EHR data	*NA*	Binary
		Length of stay	Hospitalisation characteristic	Hospitali-sation days between the admis-sion date to home discharge	EHR data	*NA*	*NR*
		Source of admission	Hospitalisation characteristic	Emergen-cy depart-ment, other hospital, or others	EHR data	*NA*	Categorical
		Triage category at the emergency department	Hospitalisation characteristic	Critical, emergen-cy, urgent, semi-urgent, non-urgent	EHR data	*NA*	Categorized

Study	Model	Predictor	Type	Definition	Measurement	Blinded assess-ment	Handling in model
		Subacute care	Hospitalisation characteristic	*NR*	EHR data	*NA*	Binary
		Length of stay of the subacute care	Hospitalisation characteristic	*NR*	EHR data	*NA*	*NR*
		Total quantity of drug dispensed	Hospitalisation characteristic	*NR*	EHR data	*NA*	*NR*
		Total prescribed dispensing duration	Hospitalisation characteristic	*NR*	EHR data	*NA*	*NR*
2023 Karpinski		*NR*	*NR*	*NR*	*NR*	*NR*	*NR*

CCI, Charlson comorbidity index; NR, not reported; NA, not applicable; COPD, chronic obstructive pulmonary disease; UNC, University of North Carolina; enPCR, equilibrated normalized protein catabolic rate; HD, haemodialysis; CART, classification and regression trees; GBDT, gradient boosted decision trees; LR, logistic regression; RF, random forest; KNN, K-nearest neighbour; CKD, chronic kidney disease; SVM, support vector machine; wFMFP, weighted factorisation machine with fuzzy partition; wFM, weighted factorisation machine; FMFP, factorisation machine with fuzzy partition; FM, factorisation machine; RBF, radial basis function; MLP, multilayer perceptron; GBM, gradient boosting machine.

Table S9. Performance metrics for models validated in haemodialysis patients (NECOSAD), peritoneal dialysis patients (NECOSAD) and advanced chronic kidney disease not on dialysis patients (EQUAL).

Population	N[a]	Events	CITL	Calibration slope	C-statistic (95% CI)
**Charlson comorbidity index model - Fried *et al.***					
HD	949	-	0.000	-0.017	-
PD	529	-	0.000	-0.037	-
aCKD-ND	1,561	-	0.000	-0.233	-
**Davies model - Fried *et al.***					
HD	949	-	0.000	0.062	-
PD	529	-	0.000	-0.062	-
**Admission model - Flythe *et al.***					
HD	3,914	242	0.032	3.441	0.59 (0.55-0.63)
PD	2,316	140	0.033	6.046	0.62 (0.56-0.68)
aCKD-ND	2,247	157	-0.030	2.108	0.59 (0.54-0.63)

CITL, calibration-in-the-large; CI, confidence interval; HD, haemodialysis; PD, peritoneal dialysis; aCKD-ND, advanced chronic kidney disease not on dialysis.
[a] For the admission model of Flythe *et al.*, N indicates the number of hospitalisations.

Table S10. Comparison of baseline characteristics between the development population of the Charlson comorbidity index model of Fried *et al.* and the external validation populations.

	Development	NECOSAD HD	NECOSAD PD	EQUAL
**Number of individuals**	415	949	529	1,561
**Age, *yrs*, mean (SD)**	55.9 (14.8)	63.6 (13.4)	53.0 (14.0)	76.9 (6.7)
**Women, n (%)**	157 (37.8)	385 (40.6)	176 (33.3)	549 (35.2)
**Black, n (%)**	64 (15.4)	23 (2.4)	12 (2.3)	22 (1.4)
**DM, n (%)**	183 (44.1)	140 (14.8)	88 (16.6)	650 (41.6)
**Albumin, *g/dL*, mean (SD)**	3.6 (0.5)	36.2 (5.0)	36.1 (5.1)	37.8 (5.2)
**BMI, *kg/m²*, mean (SD)**	27.2 (15.7)	24.7 (4.3)	24.9 (3.9)	28.3 (5.5)
**CCI, median [range]**	6 (2-15)	4 [2, 10]	3 [2, 9]	7 [4, 14]
2-3, n (%)	83 (20.0)	335 (35.3)	343 (64.8)	0 (0.0)
4-5	127 (30.6)	345 (36.4)	124 (23.4)	306 (19.6)
6-7	117 (28.2)	204 (21.5)	51 (9.6)	664 (42.5)
>7	88 (21.2)	65 (6.8)	11 (2.1)	591 (37.9)

Data shown is derived from the first imputation for each data source.
HD, haemodialysis; PD, peritoneal dialysis; yrs, years; SD, standard deviation; DM, diabetes mellitus; BMI, body mass index; CCI, Charlson comorbidity index.

Table S11. Comparison of baseline characteristics between the development population of the Davies model of Fried *et al.* and the external validation populations.

	Development	NECOSAD HD	NECOSAD PD
**Number of individuals**	415	949	529
**Age, *yrs*, mean (SD)**	55.9 (14.8)	63.6 (13.4)	53.0 (14.0)
**Women, n (%)**	157 (37.8)	385 (40.6)	176 (33.3)
**Black, n (%)**	64 (15.4)	23 (2.4)	12 (2.3)
**DM, n (%)**	183 (44.1)	140 (14.8)	88 (16.6)
**Albumin, *g/dL*, mean (SD)**	3.6 (0.5)	36.2 (5.0)	36.1 (5.1)
**BMI, *kg/m²*, mean (SD)**	27.2 (15.7)	24.8 (4.3)	24.9 (3.9)
**Davies, median [range]**	2 (0-6)	0 [0, 3]	0 [0, 2]
0, n (%)	81 (19.5)	739 (77.9)	423 (80.0)
1	93 (22.4)	188 (19.8)	105 (19.8)
2	117 (28.2)	21 (2.2)	1 (0.2)
>2	124 (29.9)	1 (0.1)	0 (0.0)

Data shown is derived from the first imputation for each data source.
HD, haemodialysis; PD, peritoneal dialysis; yrs, years; SD, standard deviation; DM, diabetes mellitus; BMI, body mass index.

Table S12. Comparison of baseline characteristics between the development population of the admission model of Flythe *et al.* and the external validation populations.

	Development	NECOSAD HD	NECOSAD PD	EQUAL
**Number of hospitalisations**	349	3,914	2,316	2,247
**Number of individuals**	349	891	523	861
**Administrative data, n (%)**				
Age, *yrs*				
≤49	99 (28.4)	501 (12.8)	782 (33.9)	0 (0.0)
50-59	86 (24.6)	638 (16.3)	619 (26.8)	0 (0.0)
60-69	94 (26.9)	1,089 (27.9)	558 (24.2)	338 (15.0)
≥70	70 (20.1)	1,678 (43.0)	347 (15.0)	1,909 (85.0)
Women	163 (46.7)	1,672 (42.7)	848 (36.6)	807 (35.9)
Black people	219 (62.7)	86 (2.2)	47 (2.0)	24 (1.1)
DM	132 (37.8)	724 (18.5)	483 (20.9)	1,026 (45.7)
Heart failure	166 (47.6)	214 (5.5)	54 (2.3)	499 (22.2)
Hypertension	249 (71.3)	3,385 (86.5)	1,934 (83.5)	1,940 (86.3)
Malignancy	25 (7.2)	166 (4.2)	52 (2.2)	567 (25.2)
**Hospital admission data, n (%)**				
Hospitalisations in the prior year ≥3	80 (22.9)	1022 (26.1)	620 (26.8)	801 (35.6)
Outpatient use of warfarin	28 (8.0)	-	-	-
Outpatient use of coumarin derivatives	-	1064 (27.2)	291 (12.6)	-
Outpatient use of antithrombotics	-	-	-	1105 (49.2)
Surgery admitting service	86 (24.6)	469 (12.0)	256 (11.1)	98 (4.4)
Systolic BP, *mmHg*				
≤110	54 (15.5)	247 (6.3)	254 (11.0)	829 (36.9)
111-175	236 (67.6)	3,340 (85.3)	1,920 (82.9)	1,243 (55.3)
≥176	59 (16.9)	327 (8.4)	142 (6.1)	175 (7.8)

Data shown is derived from the first imputation for each data source.
HD, haemodialysis; PD, peritoneal dialysis; yrs, years; DM, diabetes mellitus; BP, blood pressure.

Table S13. Overview of the reported prediction formulas of identified studies, including intercepts re-estimated in our data.

Study	Model	Re-estimated intercept	Regression coefficients
2003 Fried	CCI	HD patients: 1.465<br>PD patients: 1.409<br>aCKD-ND patients: -0.244	CCI 2-3: 0<br>CCI 4-5: 0.626<br>CCI 6-7: 0.718<br>CCI >7: 0.784<br>Albumin (g/dL): -0.223<br>Race (African American): 0.270
2003 Fried	Davies	HD patients: 2.191<br>PD patients: 1.944	Davies 0: 0<br>Davies 1: 0.351<br>Davies 2: 0.875<br>Davies >2: 1.037<br>Albumin (g/dL): -0.210<br>Race (African American): 0.307<br>Age (year): -0.007
2013 Perkins	-	-	Admitted through emergency department: 0.803<br>Admitted in transfer from other hospital: -0.455<br>Number of clinic visits (primary care or other) in 3 months prior to index admission, per completed clinic encounter: 0.098<br>Discharged to facility other than home: -1.068<br>Length of stay (index hospitalisation), per day: 0.068<br>Fluid and electrolyte disorder: 0.804<br>Other nervous system disorder: 0.872<br>Other circulatory disease: 0.913<br>COPD and/or bronchiectasis: 0.645<br>Asthma: -1.415<br>Abdominal hernia: 2.424<br>Gastrointestinal haemorrhage: 1.237<br>Other disease of kidney/ureter: 0.785<br>Calcitriol at admission: 1.543<br>Narcotic at admission: 0.564<br>Last outpatient systolic blood pressure prior to hospital admission, per 10mmHg: -0.146<br>Last outpatient diastolic blood pressure prior to admission, per 10 mmHg: 4.678<br>Last outpatient diastolic blood pressure, per 10 mmHg (quadratic term): -0.327<br>Admission white blood cell count, per 1x10^9: 0.078<br>Admission haemoglobin, per g/dL: -0.112<br>Admission serum sodium, per mmol/L: -0.084<br>Negative outpatient proteinuria testing: 0.722<br>Positive outpatient proteinuria testing: 0.640
2016 Flythe	Admission	HD patients: -1.633<br>PD patients: -1.589<br>aCKD-ND patients: -2.433	Malignancy comorbid condition: 1.012<br>Hospitalisations in the prior year >=3: 0.806<br>Outpatient use of warfarin: 0.732<br>Surgery admitting service: 0.560<br>Fistula access: 0<br>Graft access: 0.010<br>Catheter access: 0.577<br>Non-UNC-affiliated outpatient dialysis unit: 0.747<br>Admission SBP <= 110: 0.756<br>Admission systolic SBP >= 176: 0.030
2016 Flythe	Discharge	-	Malignancy comorbid condition: 0.732<br>Hospitalisations in the prior year >=3: 0.678<br>Outpatient medications >= 10: 0.525<br>Surgery admitting service: 0.554<br>Fistula access: 0<br>Graft access: 0.086<br>Catheter access: 0.599<br>Non-UNC-affiliated outpatient dialysis unit: 1.278<br>Nadir intradialytic systolic BP <90: 1.131<br>Weekend discharge day: 0.599<br>Albumin <3.3 g/dL: 1.454<br>No medication change: 0

Study	Model	Re-estimated intercept	Regression coefficients
			Medication decrease: -1.609<br>Medication increase: 0.068
2020 Wu	LR	-	ESKD admission: -1.506<br>Dialysis complications admission: -0.861<br>Admission to the same hospital as the last: -0.552<br>1 hospitalisation within 6 months: 0.096<br>2 hospitalisations within 6 months: -0.117<br>3 hospitalisations within 6 months: -0.174<br>>=4 hospitalisations within 6 months: -0.324<br>Pulmonary infections comorbidity: 0.619<br>Admitted to nephrology department: -0.155<br>Residing in northern China: 0.126<br>Residing in central China: 0.433<br>Residing in southern China: 0.357<br>Insurance type NRCMS: -0.038
2020 Wu	Stacking	-	Admission ESKD: 2<br>Admission dialysis complications: 5<br>Admission others: 7<br>Admission to different hospital than last: 0<br><4 hospitalisations within 6 months: 1<br>Pulmonary infections: 2<br>Admitted to nephrology department: 1<br>Place of residence northern China: 1<br>Place of residence central China: 2<br>Place of residence southern China: 1

A dash in the re-estimated intercept columns means that an intercept was not re-estimated because predictors were not available in our data.

CCI, Charlson comorbidity index; HD, haemodialysis; PD, peritoneal dialysis; aCKD-ND, advanced chronic kidney disease not on dialysis; COPD, chronic obstructive pulmonary disease; UNC, University of North Carolina; SBP, systolic blood pressure; BP, blood pressure; CART, classification and regression trees; GBDT, gradient boosted decision trees; LR, logistic regression; RF, random forest; ESKD, end-stage kidney disease; NRCMS, New Rural Cooperative Medical System; KNN, K-nearest neighbour; CKD, chronic kidney disease; SVM, support vector machine.

Table S14. Baseline characteristics of individuals in the development and external validation data.

	**NECOSAD HD**	**NECOSAD PD**	**EQUAL**
**Number of individuals**	1020	620	1718
**Age, *yrs*, mean (SD)**	63.6 (13.6)	52.7 (14.5)	76.3 (6.7)
**Women, n (%)**	419 (41.1)	213 (34.4)	590 (34.4)
**Cardiovascular disease, n (%)**	194 (19.0)	81 (13.1)	1056 (63.2)
**Diabetes mellitus, n (%)**	140 (13.7)	88 (14.2)	705 (42.2)
**Malignancy, n (%)**	16 (1.6)	1 (0.2)	350 (21.2)
**eGFR, *mL/min/1.73m²*, median [IQR]**	5.1 [4.1, 6.7]	5.0 [4.1, 6.0]	17.1 [13.9, 20.2]
**BMI, *kg/m²*, median [IQR]**	24.1 [22.0, 26.9]	24.2 [22.1, 26.8]	27.9 [24.6, 31.4]

Table S15. Overview of predictors included in the developed model for the risk of hospitalisation within one year after initiating dialysis.

**Predictor**	**Type**	**Definition**	**Measurement**	**Handling in model**
Age	Demographic	Age in years at dialysis initiation	Calculated from date of birth	Transformation not deemed necessary
Cardiovascular disease	Comorbidity	From CRF	Clinical diagnosis	Binary
Diabetes mellitus	Comorbidity	From CRF	Clinical diagnosis	Binary
Malignancy	Comorbidity	From CRF, excluding BCC and SCC	Clinical diagnosis	Binary
eGFR	Laboratory value	Based on creatinine using 2009 CKD-EPI equation	Just before dialyzing	Transformation not deemed necessary
BMI	Vital	From CRF enriched with height and weight	Full height and weight at study visit	Transformation not deemed necessary

NA, not applicable; CRF, case report form; BCC, basal cell carcinoma; SCC, squamous cell carcinoma; eGFR, estimated glomerular filtration rate; BMI, body mass index.

Table S16. Correlation between predictors as assessed with Spearman's rank correlation coefficient to assess collinearity in the developed model.

	Age	CVD	DM	Malignancy	eGFR	BMI
**Age**	1	0.069	-0.059	0.056	0.084	0.000
**CVD**	0.069	1	0.003	-0.011	0.016	-0.030
**DM**	-0.059	0.003	1	0.008	0.143	0.184
**Malignancy**	0.056	-0.011	0.008	1	0.020	-0.064
**eGFR**	0.082	0.016	0.143	0.020	1	-0.023
**BMI**	0.000	-0.030	0.184	-0.064	-0.023	1

Table S17. Regression coefficients for all developed prediction models. To get individual predicted risks, see the explanation in Calculating individual risks.

	Original model			Sensitivity analysis: hospitalisation of ≥3 days		
	Fine-Gray	Cox PH	Logistic regression	Fine-Gray	Cox PH	Logistic regression
Baseline hazard/intercept	0.705	0.728	-0.590	0.195	0.203	-2.110
Age	0.005	0.007	0.008	0.012	0.013	0.013
CVD	0.264	0.283	0.410	0.162	0.174	0.171
DM	0.328	0.333	0.492	0.711	0.721	0.822
Malignancy	0.841	0.885	1.387	-0.030	0.053	-0.100
eGFR	0.003	0.004	0.008	-0.013	-0.011	-0.013
BMI	-0.004	-0.004	0.003	-0.005	-0.007	-0.006
LP of sample	0.261	0.329	-	0.538	0.560	-

The reported baseline hazard is the baseline hazard at one year follow-up.
PH, proportional hazards, CVD, cardiovascular disease; DM, diabetes mellitus; eGFR, estimated glomerular filtration rate; BMI, body mass index; LP, linear predictor.

Supplemental results

Re-estimating models

We wrote a function, `reestimate()`, in R that allows the re-estimation of our developed model in other data. The assumptions for collinearity, linearity of continuous predictors, and proportional hazards are checked. Subsequently, the model is developed, either on imputed data or non-imputed data, and apparent validation is performed. This consists of calibration-in-the-large (perfect at 1), the calibration slope (perfect at 1), Wolbers' adaptation of Harrell's C-statistic (3), and a calibration plot. The calibration plot uses jack-knife pseudo-values, with a locally estimated scatterplot smoother (LOESS) with a span of 0.75. Additionally, deciles of observed and predicted risk are plotted. Five arguments can be supplied.

Argument	Details
data	A data frame or tibble with at least the following variables:<br>- studynr: unique identifier for an individual<br>- age: individual's age at baseline in years<br>- cardiovasc/dm/hf/mal: whether an individual has cardiovascular disease/diabetes mellitus/heart failure/malignancy, respectively, at baseline [0 = no, 1 = yes]<br>- tte: time to event for an individual in days<br>- event: event the individual experiences; a factor with levels 'censoring', 'hospitalization', and 'death'
imputed	Whether the data is imputed (`TRUE`) or not (`FALSE`). If data is imputed, the data supplied should contain a variable .imp with the imputation number. If data is not imputed, no values should be missing.
event_label	This argument indicates where the labels should be placed on the x-axis of the histogram with predicted probabilities, to avoid overlap with the histogram bars. If "`dynamic`", the labels are placed at the least predicted probability that was predicted at least once. Otherwise, a number between 0 and 1 can be supplied specifying the location on the x-axis.
annotation	Whether an annotation should be added to the calibration plot. If "", no annotation is added. Otherwise, supply the annotation. The annotation is placed in the upper left corner of the plot and is optimized for a single letter (e.g. to denote *figure A*).
save_location	Location on the device where figures should be saved.
save_name_prefix	What prefix should be added before the name of each figure (e.g. "pd_").

To run the function, the following packages should be installed and loaded:

```
# Load packages
library("dplyr",              # Data manipulation
        "magrittr",           # Efficient piping
        "broom",              # Converting statistical objects
        "stringr",            # Working with strings
        "rms",                # Checking assumptions
        "splines",            # Splines
        "cowplot",            # Data viz. add-ons
        "survival",           # Modelling
        "ggplot2",            # Data visualization
        "intsurv"             # Package for calculating C-statistic
)
```

The function can then be loaded and run as follows:

```
# Load function
source("https://raw.githubusercontent.com/rjjanse/hosps/main/reestimate.R")

# Re-estimate
reestimate(dat_necosad_pd, save_location = "C:/users/", save_name_prefix = "pd_", event_label =
0.8, annotation = "A")
```

The output of the function, for instance for our peritoneal dialysis data, is as follows:

```
The plots for linearity and proportional hazards are available in C:/users/
The correlation matrix for collinearity is:
               age cardiovasc     dm    mal   egfr    bmi
age          1.000      0.172  0.024 -0.015 -0.157  0.199
cardiovasc   0.172      1.000 -0.075 -0.016 -0.136 -0.005
dm           0.024     -0.075  1.000 -0.016  0.130  0.147
mal         -0.015     -0.016 -0.016  1.000  0.067  0.055
egfr        -0.157     -0.136  0.130  0.067  1.000 -0.001
bmi          0.199     -0.005  0.147  0.055 -0.001  1.000

The baseline hazard and coefficients of the re-estimated model are:

Baseline hazard            0.723100
Age                        0.007877
Cardiovascular disease     0.176000
Diabetes mellitus          0.318100
Malignancy               -13.910000
eGFR                      -0.031740
BMI                        0.018900

To calculate individual risks of hospitalization, do the following:
1. Calculate each individual's linear predictor:
LP = age (years) * 0.008 + presence of cardiovascular disease * 0.176 + presence of diabetes
mellitus * 0.318 + presence of malignancy * -13.910 + eGFR (mL/min/1.73m2) * -0.032 + BMI
(kg/m2) * 0.019

2. Transform the linear predictor into the risk:
1 - exp(0.723 ^ exp(LP - 0.202))

The validation metrics are:
Calibration-in-the-large        Calibration slope              C statistic
                   1.011                    1.929         0.55 (0.52-0.58)
```

Calculating individual risks

Fine-Gray model

To calculate individual risks for the Fine-Gray model, first calculate the linear predictor (LP) for each individual:

$$LP = age * 0.005 + CVD * 0.264 + DM * 0.328 + mal * 0.841 + eGFR * 0.003 + BMI * \ -0.004$$

Age is the age at baseline in years, CVD is the presence of cardiovascular disease at baseline, DM is the presence of diabetes mellitus at baseline, mal is the presence of malignancy at baseline, eGFR is the estimated glomerular filtration rate calculated by the 2009 CKD-EPI formula at baseline in mL/min/1.73m^2, and BMI is the body mass index at baseline in kg/m^2. The LP can then be transformed into individual risks (pr) with the following formula:

$$pr = 1 - \exp(-0.705) \wedge \exp\,(LP - 0.261)$$

For instance, a 60 year old individual with cardiovascular disease and diabetes, with an eGFR of 4.3 mL/min/1.73m^2 and a BMI of 27 kg/m^2 would have a LP of:

$$LP = 60 * 0.005 + 1 * 0.264 + 1 * 0.328 + 0 * 0.841 + 4.3 * 0.003 + 27 * \ -0.004 = 0.797$$

This gives an individual risk of:

$$pr = 1 - \exp(-0.705) \wedge \exp(0.762 - 0.272) = 0.689 = 68.9\%$$

Cox proportional hazards model

To calculate individual risks for the Cox proportional hazards model, first calculate the linear predictor (LP) for each individual:

$$LP = age * 0.007 + CVD * 0.283 + DM * 0.333 + mal * 0.885 + eGFR * 0.004 + BMI * -0.004$$

Age is the age at baseline in years, CVD is the presence of cardiovascular disease at baseline, DM is the presence of diabetes mellitus at baseline, mal is the presence of malignancy at baseline, eGFR is the estimated glomerular filtration rate calculated by the 2009 CKD-EPI formula at baseline in mL/min/1.73m^2, and BMI is the body mass index at baseline in kg/m^2. The LP can then be transformed into individual risks (pr) with the following formula:

$$pr = 1 - \exp(-0.728) \wedge \exp(LP - 0.329)$$

For instance, a 60 year old individual with cardiovascular disease and diabetes, with an eGFR of 4.3 mL/min/1.73m^2 and a BMI of 27 kg/m^2 would have a LP of:

$$LP = 60 * 0.007 + 1 * 0.283 + 1 * 0.333 + 0 * 0.885 + 4.3 * 0.004 + 27 * -0.004 = 0.945$$

This gives an individual risk of:

$$pr = 1 - \exp(-0.728) \wedge \exp(0.945 - 0.329) = 0.740 = 74.0\%$$

Logistic regression model

To calculate individual risks for the Cox proportional hazards model, first calculate the linear predictor (LP) for each individual:

$$LP = age * 0.008 + CVD * 0.410 + DM * 0.492 + mal * 1.387 + eGFR * 0.008 + BMI * 0.003$$

Age is the age at baseline in years, CVD is the presence of cardiovascular disease at baseline, DM is the presence of diabetes mellitus at baseline, mal is the presence of malignancy at baseline, eGFR is the estimated glomerular filtration rate calculated by the 2009 CKD-EPI formula at baseline in mL/min/1.73m^2, and BMI is the body mass index at baseline in kg/m^2. The LP can then be transformed into individual risks (pr) with the following formula:

$$pr = \frac{1}{1 + e^{-(-0.590 + LP)}}$$

For instance, a 60 year old individual with cardiovascular disease and diabetes, with an eGFR of 4.3 mL/min/1.73m^2 and a BMI of 27 kg/m^2 would have a LP of:

$$LP = 60 * 0.008 + 1 * 0.410 + 1 * 0.492 + 0 * 1.387 + 4.3 * 0.008 + 27 * 0.003 = 1.497$$

This gives an individual risk of:

$$pr = \frac{1}{1 + e^{-(-0.590 + 1.497)}} = 0.712 = 71.2\%$$

Checklists

Preferred Reporting Items for Systematic Reviews and Meta-Analyses (PRISMA)

Section and Topic	Item #	Checklist item	Location where item is reported
**TITLE**			
Title	1	Identify the report as a systematic review.	Title
**ABSTRACT**			
Abstract	2	See the PRISMA 2020 for Abstracts checklist.	Abstract
**INTRODUCTION**			
Rationale	3	Describe the rationale for the review in the context of existing knowledge.	Background, 3rd paragraph
Objectives	4	Provide an explicit statement of the objective(s) or question(s) the review addresses.	Background, 4th paragraph
**METHODS**			
Eligibility criteria	5	Specify the inclusion and exclusion criteria for the review and how studies were grouped for the syntheses.	Methods, 2nd paragraph
Information sources	6	Specify all databases, registers, websites, organisations, reference lists and other sources searched or consulted to identify studies. Specify the date when each source was last searched or consulted.	Methods, 2nd paragraph
Search strategy	7	Present the full search strategies for all databases, registers and websites, including any filters and limits used.	Supplemental methods – search string
Selection process	8	Specify the methods used to decide whether a study met the inclusion criteria of the review, including how many reviewers screened each record and each report retrieved, whether they worked independently, and if applicable, details of automation tools used in the process.	Methods, 2nd paragraph
Data collection process	9	Specify the methods used to collect data from reports, including how many reviewers collected data from each report, whether they worked independently, any processes for obtaining or confirming data from study investigators, and if applicable, details of automation tools used in the process.	Methods, 3rd paragraph
Data items	10a	List and define all outcomes for which data were sought. Specify whether all results that were compatible with each outcome domain in each study were sought (e.g. for all measures, time points, analyses), and if not, the methods used to decide which results to collect.	Methods, 3rd paragraph
	10b	List and define all other variables for which data were sought (e.g. participant and intervention characteristics, funding sources). Describe any assumptions made about any missing or unclear information.	Methods, 3rd paragraph
Study risk of bias assessment	11	Specify the methods used to assess risk of bias in the included studies, including details of the tool(s) used, how many reviewers assessed each study and whether they worked independently, and if applicable, details of automation tools used in the process.	Methods, 3rd paragraph
Effect measures	12	Specify for each outcome the effect measure(s) (e.g. risk ratio, mean difference) used in the synthesis or presentation of results.	Methods, 3rd paragraph
Synthesis methods	13a	Describe the processes used to decide which studies were eligible for each synthesis (e.g. tabulating the study intervention characteristics and comparing against the planned groups for each synthesis (item #5)).	*NA*
	13b	Describe any methods required to prepare the data for presentation or synthesis, such as handling of missing summary statistics, or data conversions.	*NA*
	13c	Describe any methods used to tabulate or visually display results of individual studies and syntheses.	*NA*
	13d	Describe any methods used to synthesize results and provide a rationale for the choice(s). If meta-analysis was performed, describe the model(s), method(s) to identify the presence and extent of statistical heterogeneity, and software package(s) used.	*NA*
	13e	Describe any methods used to explore possible causes of heterogeneity among study results (e.g. subgroup analysis, meta-regression).	*NA*
	13f	Describe any sensitivity analyses conducted to assess robustness of the synthesized results.	*NA*
Reporting bias assessment	14	Describe any methods used to assess risk of bias due to missing results in a synthesis (arising from reporting biases).	*NA*

Section and Topic	Item #	Checklist item	Location where item is reported
Certainty assessment	15	Describe any methods used to assess certainty (or confidence) in the body of evidence for an outcome.	Methods - *External validation*
**RESULTS**			
Study selection	16a	Describe the results of the search and selection process, from the number of records identified in the search to the number of studies included in the review, ideally using a flow diagram.	Figure 1
	16b	Cite studies that might appear to meet the inclusion criteria, but which were excluded, and explain why they were excluded.	*NA*
Study characteristics	17	Cite each included study and present its characteristics.	Results - *Identified models*
Risk of bias in studies	18	Present assessments of risk of bias for each included study.	Results - *Quality of models*
Results of individual studies	19	For all outcomes, present, for each study: (a) summary statistics for each group (where appropriate) and (b) an effect estimate and its precision (e.g. confidence/credible interval), ideally using structured tables or plots.	Results - *External validation*
Results of syntheses	20a	For each synthesis, briefly summarise the characteristics and risk of bias among contributing studies.	*NA*
	20b	Present results of all statistical syntheses conducted. If meta-analysis was done, present for each the summary estimate and its precision (e.g. confidence/credible interval) and measures of statistical heterogeneity. If comparing groups, describe the direction of the effect.	Results - *External validation*
	20c	Present results of all investigations of possible causes of heterogeneity among study results.	*NA*
	20d	Present results of all sensitivity analyses conducted to assess the robustness of the synthesized results.	*NA*
Reporting biases	21	Present assessments of risk of bias due to missing results (arising from reporting biases) for each synthesis assessed.	*NA*
Certainty of evidence	22	Present assessments of certainty (or confidence) in the body of evidence for each outcome assessed.	*NA*
**DISCUSSION**			
Discussion	23a	Provide a general interpretation of the results in the context of other evidence.	Discussion, 1st paragraph
	23b	Discuss any limitations of the evidence included in the review.	Discussion, 3rd paragraph
	23c	Discuss any limitations of the review processes used.	*NA*
	23d	Discuss implications of the results for practice, policy, and future research.	Discussion
**OTHER INFORMATION**			
Registration and protocol	24a	Provide registration information for the review, including register name and registration number, or state that the review was not registered.	Methods, 1st paragraph
	24b	Indicate where the review protocol can be accessed, or state that a protocol was not prepared.	Methods, 1st paragraph
	24c	Describe and explain any amendments to information provided at registration or in the protocol.	Supplemental materials - *Protocol deviations*
Support	25	Describe sources of financial or non-financial support for the review, and the role of the funders or sponsors in the review.	Funding statement
Competing interests	26	Declare any competing interests of review authors.	Conflict of interest statement
Availability of data, code and other materials	27	Report which of the following are publicly available and where they can be found: template data collection forms; data extracted from included studies; data used for all analyses; analytic code; any other materials used in the review.	Data availability statement

NA, not applicable.

Transparent reporting of a multivariable prediction model for individual prognosis or diagnosis (TRIPOD) statement

Section/Topic	Item	Checklist Item	Location
**Title and abstract**			
Title	1	Identify the study as developing and/or validating a multivariable prediction model, the target population, and the outcome to be predicted.	Title
Abstract	2	Provide a summary of objectives, study design, setting, participants, sample size, predictors, outcome, statistical analysis, results, and conclusions.	Abstract
**Introduction**			
Background and objectives	3a	Explain the medical context (including whether diagnostic or prognostic) and rationale for developing or validating the multivariable prediction model, including references to existing models.	Background, 1st through 3rd paragraph
	3b	Specify the objectives, including whether the study describes the development or validation of the model or both.	Background, 4th paragraph
**Methods**			
Source of data	4a	Describe the study design or source of data (e.g. randomized trial, cohort, or registry data), separately for the development and validation data sets, if applicable.	Methods - Data sources
	4b	Specify the key study dates, including start of accrual; end of accrual; and, if applicable, end of follow-up.	Methods - Data sources
Participants	5a	Specify key elements of the study setting (e.g. primary care, secondary care, general population) including number and location of centres.	Methods - Data sources
	5b	Describe eligibility criteria for participants.	Methods - Model development
	5c	Give details of treatments received, if relevant.	*NA*
Outcome	6a	Clearly define the outcome that is predicted by the prediction model, including how and when assessed.	Methods - Model development
	6b	Report any actions to blind assessment of the outcome to be predicted.	Methods - *Model development*
Predictors	7a	Clearly define all predictors used in developing or validating the multivariable prediction model, including how and when they were measured.	Table S13
	7b	Report any actions to blind assessment of predictors for the outcome and other predictors.	Methods - *Model development*
Sample size	8	Explain how the study size was arrived at.	Methods - *Statistical analysis*, Protocol
Missing data	9	Describe how missing data were handled (e.g. complete-case analysis, single imputation, multiple imputation) with details of any imputation method.	Methods - *Statistical analysis*
Statistical analysis methods	10a	Describe how predictors were handled in the analyses.	Table S13
	10b	Specify type of model, all model-building procedures (including any predictor selection), and method for internal validation.	Methods - *Model development* and Methods - *Statistical analysis*
	10c	For validation, describe how the predictions were calculated.	Methods - *External validation*
	10d	Specify all measures used to assess model performance and, if relevant, to compare multiple models.	Methods - *External validation*
	10e	Describe any model updating (e.g. recalibration) arising from the validation, if done.	Methods - *External validation*
Risk groups	11	Provide details on how risk groups were created, if done.	*NA*
Development vs. validation	12	For validation, identify any differences from the development data in setting, eligibility criteria, outcome, and predictors.	Supplemental tables 8-11
**Results**			
Participants	13a	Describe the flow of participants through the study, including the number of participants with and without the outcome and, if applicable, a summary of the follow-up time. A diagram may be helpful.	Figure S4
	13b	Describe the characteristics of the participants (basic demographics, clinical features, available predictors), including the number of participants with missing data for predictors and outcome.	Table S12
	13c	For validation, show a comparison with the development data of the distribution of important variables (demographics, predictors and outcome).	Supplemental tables 8-11
Model development	14a	Specify the number of participants and outcome events in each analysis.	Table 4, Table S7
	14b	If done, report the unadjusted association between each candidate predictor and outcome.	*NA*

Section/Topic	Item	Checklist Item	Location
Model specification	15a	Present the full prediction model to allow predictions for individuals (i.e. all regression coefficients, and model intercept or baseline survival at a given time point).	Supplemental results - *Calculating individual risks*
	15b	Explain how to the use the prediction model.	Supplemental results - *Calculating individual risks*
Model performance	16	Report performance measures (with CIs) for the prediction model.	Table 4, Table S7
Model-updating	17	If done, report the results from any model updating (i.e. model specification, model performance).	*NA*
**Discussion**			
Limitations	18	Discuss any limitations of the study (such as nonrepresentative sample, few events per predictor, missing data).	Discussion, 7th paragraph
Interpretation	19a	For validation, discuss the results with reference to performance in the development data, and any other validation data.	Discussion, 3rd paragraph
	19b	Give an overall interpretation of the results, considering objectives, limitations, results from similar studies, and other relevant evidence.	Discussion
Implications	20	Discuss the potential clinical use of the model and implications for future research.	Discussion
**Other information**			
Supplementary information	21	Provide information about the availability of supplementary resources, such as study protocol, Web calculator, and data sets.	Throughout manuscript
Funding	22	Give the source of funding and the role of the funders for the present study.	Funding statement

NA, not applicable.

Checklist for critical Appraisal and data extraction for systematic Reviews of prediction Modelling Studies (CHARMS)

Key items	Reported
**Source of data**	
Source of data (e.g. cohort, case-control, randomized trial participants, or registry data)	Table 1
Participant eligibility and recruitment method (e.g. consecutive participants, location, number of centres, setting, inclusion and exclusion criteria)	Table S2
Participant description	Table 1
Details of treatments received, if relevant	*Not applicable*
Study dates	Table 1
**Outcome(s) to be predicted**	
Definition and method for measurement of outcome	Table 1, Table S1
Was the same outcome definition (and method for measurement) used in all patients?	Table S1
Type of outcome (e.g. single or combined endpoints)	Table S1
Was the outcome assessed without knowledge of the candidate predictors (i.e. blinded)?	Table S1
Were candidate predictors part of the outcome (e.g. in panel or consensus diagnosis)?	Table S1
Time of outcome occurrence or summary of duration of follow-up	Table 1
**Candidate predictors (or index tests)**	
Number and type of predictors (e.g. demographics, patient history, physical examination, additional testing, disease characteristics)	Table S6
Definition and method for measurement of candidate predictors	Table S6
Timing of predictor measurement (e.g. at patient presentation, at diagnosis, at treatment initiation)	Table S6
Were predictors assessed blinded for outcome, and for each other (if relevant)?	Table S6
Handling of predictors in the modelling (e.g. continuous, linear, non-linear transformations or categorised)	Table S6
**Sample size**	
Number of participants and number of outcomes/events	Table 2
Number of outcomes/events in relation to the number of candidate predictors (Events Per Variable)	Table S1
**Missing data**	
Number of participants with any missing value (include predictors and outcomes)	Table S2
Number of participants with missing data for each predictor	Table S2
Handling of missing data (e.g. complete-case analysis, imputation, or other methods)	Table S2
**Model development**	
Modelling method (e.g. logistic, survival, neural network, or machine learning techniques)	Table 2
Modelling assumptions satisfied	Table S2
Method for selection of predictors for inclusion in multivariable modelling (e.g. all candidate predictors, pre-selection based on unadjusted association with the outcome)	Table S2
Method for selection of predictors during multivariable modelling (e.g. full model approach, backward or forward selection) and criteria used (e.g. p-value, Akaike Information Criterion)	Table 2
Shrinkage of predictor weights or regression coefficients (e.g. no shrinkage, uniform shrinkage, penalized estimation)	Table 2
**Model performance**	
Calibration (calibration plot, calibration slope, Hosmer-Lemeshow test) and Discrimination (C-statistic, D-statistic, log-rank) measures with confidence intervals	Table 2, Table S3
Classification measures (e.g. sensitivity, specificity, predictive values, net reclassification improvement) and whether a-priori cut points were used	Table 2, Table S3
**Model evaluation**	
Method used for testing model performance: development dataset only (random split of data, resampling methods e.g. bootstrap or cross-validation, none) or separate external validation (e.g. temporal, geographical, different setting, different investigators)	Table 2
In case of poor validation, whether model was adjusted or updated (e.g. intercept recalibrated, predictor effects adjusted, or new predictors added)	Table S3
**Results**	
Final and other multivariable models (e.g. basic, extended, simplified) presented, including predictor weights or regression coefficients, intercept, baseline survival, model performance measures (with standard errors or confidence intervals)	Table S11
Any alternative presentation of the final prediction models, e.g. sum score, nomogram, score chart, predictions for specific risk subgroups with performance	Table S2
Comparison of the distribution of predictors (including missing data) for development and validation datasets	Table S2
**Interpretation and discussion**	
Interpretation of presented models (confirmatory, i.e. model useful for practice versus exploratory, i.e. more research needed)	Table S4
Comparison with other studies, discussion of generalizability, strengths and limitations.	Table S2

Supplemental references

1. Aalen OO, Johansen S. An Empirical Transition Matrix for Non-Homogeneous Markov Chains Based on Censored Observations. Scandinavian Journal of Statistics. 1978;5(3):141-50.

2. Gerds TA, Andersen PK, Kattan MW. Calibration plots for risk prediction models in the presence of competing risks. Stat Med. 2014;33(18):3191-203.

3. Wolbers M, Blanche P, Koller MT, Witteman JC, Gerds TA. Concordance for prognostic models with competing risks. Biostatistics. 2014;15(3):526-39.
